# *NGMASTER: in silico* multi-antigen sequence typing for *Neisseria gonorrhoeae*

**DOI:** 10.1099/mgen.0.000076

**Published:** 2016-08-25

**Authors:** Jason C. Kwong, Anders Gonçalves da Silva, Kristin Dyet, Deborah A. Williamson, Timothy P. Stinear, Benjamin P. Howden, Torsten Seemann

**Affiliations:** ^1^​ Doherty Applied Microbial Genomics, Department of Microbiology & Immunology, University of Melbourne, at the Peter Doherty Institute for Infection & Immunity, Melbourne, Australia; ^2^​ Department of Microbiology & Immunology, University of Melbourne, at the Peter Doherty Institute for Infection & Immunity, Melbourne, Australia; ^3^​ Microbiological Diagnostic Unit Public Health Laboratory, University of Melbourne, at the Peter Doherty Institute for Infection & Immunity, Melbourne, Australia; ^4^​ Institute of Environmental Science and Research, Wellington, New Zealand; ^5^​ Victorian Life Sciences Computation Initiative, University of Melbourne, Carlton, Australia

**Keywords:** Neisseria gonorrhoeae, Multi-antigen sequence typing, NG-MAST, Whole-genome sequencing, In silico typing

## Abstract

Whole-genome sequencing (WGS) provides the highest resolution analysis for comparison of bacterial isolates in public health microbiology. However, although increasingly being used routinely for some pathogens such as *Listeria monocytogenes* and *Salmonella enterica*, the use of WGS is still limited for other organisms, such as *Neisseria gonorrhoeae*. Multi-antigen sequence typing (NG-MAST) is the most widely performed typing method for epidemiological surveillance of gonorrhoea. Here, we present *NGMASTER,* a command-line software tool for performing *in silico* NG-MAST on assembled genome data. *NGMASTER* rapidly and accurately determined the NG-MAST of 630 assembled genomes, facilitating comparisons between WGS and previously published gonorrhoea epidemiological studies. The source code and user documentation are available at https://github.com/MDU-PHL/ngmaster.

## Data Summary

1. The Python source code for *NGMASTER* is available from GitHub under GNU GPL v2. (URL: https://github.com/MDU-PHL/ngmaster)
2. The software is installable via the Python ‘pip’ package management system. Install using ‘pip install – user git https://github.com/MDU-PHL/ngmaster.git’
3. Sequencing data used are available for download from the EBI European Nucleotide Archive under BioProject accessions PRJEB2999, PRJNA29335, PRJNA266539, PRJNA298332, and PRJEB14168.

## Impact Statement

Whole-genome sequencing (WGS) offers the potential for high-resolution comparative analyses of microbial pathogens. However, there remains a need for backward compatibility with previous molecular typing methods to place genomic studies in context. NG-MAST is currently the most widely used method for epidemiological surveillance of *Neisseria gonorrhoeae*. We present *NGMASTER*, a command-line software tool for performing multi-antigen sequence typing (NG-MAST) of *Neisseria gonorrhoeae* from WGS data. This tool is targeted at clinical and research microbiology laboratories that have performed WGS of *N. gonorrhoeae* isolates and wish to understand the molecular context of their data in comparison to previously published epidemiological studies. As WGS becomes more routinely performed, *NGMASTER* has been developed to completely replace PCR-based NG-MAST, reducing time and labour costs.

## Introduction

*Neisseria gonorrhoeae* is one of the most common sexually transmitted bacterial infections worldwide. There is growing concern about the global spread of resistant epidemic clones, with extensively drug-resistant gonorrhoea being listed as an urgent antimicrobial resistance threat (CDC, 2013; WHO, 2014).

Multi-antigen sequence typing of *N. gonorrhoeae* (NG-MAST) has been important in tracking these resistant clones, such as the NG-MAST 1407 clone associated with decreased susceptibility to third-generation cephalosporins (Unemo & Dillon, 2011). It involves sequence-based typing using established PCR primers of two highly variable and polymorphic outer membrane protein genes, *porB* and *tbpB* by comparing the sequences to an open-access database (http://www.ng-mast.net/) (Martin* et al.*, 2004). Although NG-MAST is the most frequently performed molecular typing method for *N. gonorrhoeae*, it requires multiple PCR amplification and sequencing reactions, making it more laborious than other gonococcal typing methods such as single *porB* gene sequencing, or fragment analysis methods such as multiple locus variable-number tandem repeat analysis (MLVA) (Heymans* et al.*, 2012).

Whole-genome sequencing (WGS) is increasingly being used for molecular typing and epidemiological investigation of microbial pathogens as it provides considerably higher resolution. A number of studies using genomic data to understand the epidemiology of *N. gonorrhoeae* have already been published (Grad* et al.*, 2014) (Demczuk* et al.*, 2015) (Ezewudo* et al.*, 2015) (Demczuk* et al.*, 2016). However, the ability to perform retrospective comparisons with previous epidemiological studies is reliant on conducting both traditional typing (such as NG-MAST) as well as more modern WGS analyses on the same isolates.

*NGMASTER* is a command-line software tool for rapidly determining NG-MAST types *in silico* from genome assemblies of *N. gonorrhoeae*.

## Description

*NGMASTER* is an open source tool written in Python and released under a GPLv2 Licence. The source code can be downloaded from Github (https://github.com/MDU-PHL/ngmaster). It has two software dependencies: *isPcr* (http://hgwdev.cse.ucsc.edu/~kent/src/) and BioPython (Cock* et al.*, 2009), and uses the allele databases publicly available at http://www.ng-mast.net/, which *NGMASTER *can automatically download and update locally for running.

*NGMASTER* is based on the laboratory method published by Martin* et al.* (2004), and uses *isPcr* to retrieve allele sequences from a user-specified genome assembly in FASTA format by locating the flanking primers. These allele sequences are trimmed to a set length from starting key motifs in conserved gene regions, and then checked against the allele databases. Results are printed in machine readable tab- or comma-separated format.

## Methods

*NGMASTER* was validated against 630 publicly available *N. gonorrhoeae* genome sequences derived from published studies (Table 1). A PubMed search for published studies with *N. gonorrhoea* whole-genome sequencing data was conducted (on 4 May, 2016) using the search terms ‘Neisseria gonorrhoeae’ and ‘whole-genome sequencing’. We excluded studies with less than 20 isolates, and those that did not publish NG-MAST results or make their raw sequencing data available. Our search identified three studies, contributing 572 sequences for testing that had undergone manual *in silico* NG-MAST from WGS data (Demczuk* et al.*, 2015; Demczuk* et al.*, 2016; Grad* et al.*, 2014), including the fully assembled reference genome NCCP11945 (Chung *et al.*, 2008). The panel of isolates also included the genome sequencing data for eight well characterised WHO reference genomes with published NG-MAST results. Raw WGS data for these sequences were retrieved from the European Nucleotide Archive (ENA). Average sequencing depth was >30× for all ENA sequences, with a combination of 100 bp, 250 bp and 300 bp paired-end Illumina reads. In addition, we tested an additional 50 local isolates that had undergone ‘traditional’ NG-MAST by PCR and Sanger sequencing (Martin* et al.*, 2004). These isolates underwent WGS on the Illumina MiSeq/NextSeq using Nextera libraries and manufacturer protocols, each with an average sequencing depth >50×. The raw sequencing reads for these local isolates have been uploaded to the ENA (BioProject accession PRJEB14168).

**Table 1.** Concordance between *NGMASTER* results from draft genome assemblies using *MEGAHIT* and *SPAdes*, and previously published NG-MAST results

	*MEGAHIT*	*SPAdes*	Two-stage¶	Total
PRJEB2999*	176 (95 %)	184 (99 %)	184 (99 %)	186
PRJNA29335†	–	–	–	1
PRJNA266539‡	162 (91 %)	169 (94 %)	178 (99 %)	179
PRJNA298332§	199 (93 %)	207 (97 %)	208 (97 %)	214
PRJEB14168||	50 (100 %)	50 (100 %)	50 (100 %)	50
Total	587 (93 %)	610 (97 %)	620 (98 %)	630

*Grad *et al.* (2014)

†Demczuk *et al.*, (2015)

‡Demczuk *et al.*(2016)

§Closed reference genome NCCP11945 (Genbank accession CP001050.1) – *in silico* NG-MAST results reported by Demczuk* et al.* (2015).

||Local isolates with NG-MAST performed by PCR/Sanger sequencing.

¶Two-stage assembly: 1. NGMASTER run using rapid assembly with *MEGAHIT*; 2. *NGMASTER* also run using *SPAdes* if there was no result or a mixed result using *MEGAHIT* assembly.

Sequencing reads were trimmed to clip Illumina adapters and low-quality sequences (minimum Q20) using *Trimmomatic* v0.35 (Bolger* et al.*, 2014). Draft genomes were assembled *de novo* with *MEGAHIT* v1.0.3 and *SPAdes* v3.7.1 (Li* et al.*, 2015) (Bankevich* et al.*, 2012) to investigate whether the faster, but approximate genome assembler, *MEGAHIT*, would be sufficient for *NGMASTER*. A list of the commands and parameters used is included in Appendix 1.

The *de novo* assembled draft genomes and the fully assembled NCCP11945 reference genome in FASTA format were used as input to *NGMASTER* with the overall results shown in Table 1. Complete *NGMASTER* results with sequencing and assembly metrics are included in Appendix 2. Running *NGMASTER* on 630 genome assemblies using a single Intel(R) Xeon(R) 2.3GHz CPU core was completed in less than two minutes.

Overall, *NGMASTER* assigned NG-MAST types that were concordant with published results for 93 % of the tested *N. gonorrhoeae* genomes using *MEGAHIT* assemblies, and 97 % using *SPAdes* assemblies. Notably, comparisons with results from traditional NG-MAST were 100 % concordant (58/58), including 50 local isolates and the eight well-characterised WHO reference isolates. Reasons for discordant results are shown in Table 2. In general, running *NGMASTER* using *SPAdes* assemblies resolved more NG-MAST types than when using *MEGAHIT* assemblies. However, ten genomes assembled with *SPAdes* v3.7.1 were found to have assembly errors in either *por* or *tbpB* introduced at the repeat resolution stage of the *SPAdes* assembly process, resulting in discordant NG-MAST types for those isolates (major errors). Running *NGMASTER* on preliminary contigs prior to this process (in particular, on the ‘before_rr.fasta’ intermediate file generated by *SPAdes* in the assembly output folder) or disabling repeat resolution using the flag ‘--disable-rr’ when running *SPAdes* alleviated these major errors, and were concordant with *MEGAHIT* results and the published results (Appendix 2). In contrast, minor errors (due to incomplete NG-MAST types or multiple alleles detected) were more frequent using *MEGAHIT* assemblies, particularly those with poor assembly metrics (e.g. >500 contigs, N50<10 kbp). When *MEGAHIT* assemblies successfully produced complete *NGMASTER* results, these NG-MAST types were highly concordant with the published results.

**Table 2.** Reasons for discordant results between *NGMASTER* and published data using *SPAdes* assemblies

Reason for discordant result	*MEGAHIT*	*SPAdes*
Major errors (incorrect result)		
Assembly error	0	10
Minor errors (incomplete/missing result)		
Alternate conserved key motif	1	1
Multiple alleles detected	6	2
Allele not detected	29	0
Errors in published data		
Possible sequence mix-up in published data	4	4
Probable transcription error in published data	1	1
Error in published data	1	1

To overcome this issue, a two-stage assembly approach was also tested, where a draft genome was first assembled using *MEGAHIT* for initial testing. If a complete NG-MAST result was obtained, this was recorded as the final result for that isolate. If the result was incomplete or suggested multiple alleles were present, the genome was also assembled using *SPAdes*. Using this combined approach, 620 out of 630 (98 %) NG-MAST types derived from *NGMASTER* were concordant with the published results, with only 42 genomes requiring additional assembly with the slower *SPAdes* assembler.

For the remaining ten discordant results, seven of these were likely to be due to errors in the published data, including for NCCP11945. A further two isolates were found to have multiple *tbpB* alleles in both *SPAdes* and *MEGAHIT* assemblies, with the dominant allele (indicated by higher read coverage and better flanking assembly) matching the published result. The *tbpB* allele for the final isolate was not able to be determined by *NGMASTER* due to a mutation in the conserved starting key motif required for sequence trimming to a standard size.

## Issues with implementation

**Fig. 1.**
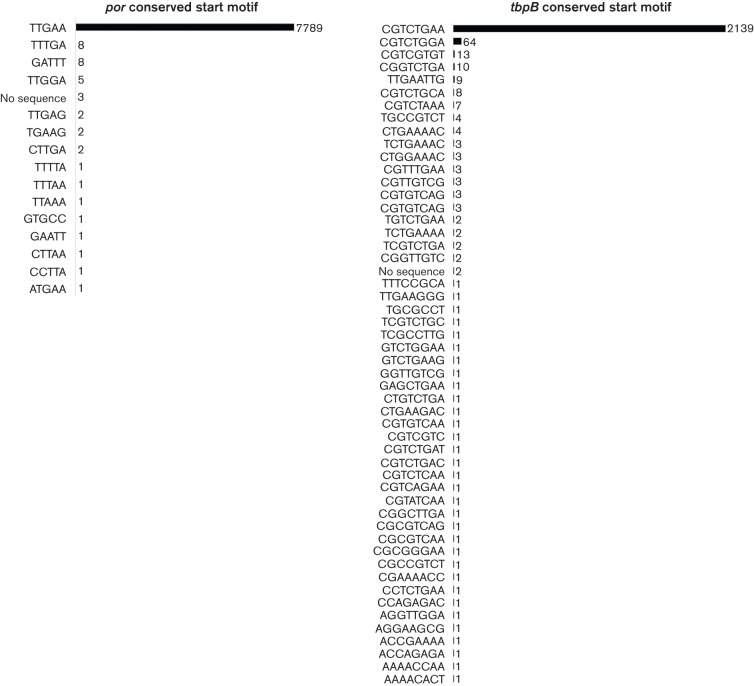
Number and frequency of alternate starting key motifs within ‘conserved’ gene regions for trimming allele sequences.

The NG-MAST procedure involves sequencing the internal regions of *por* and *tbpB* that encode two variable outer membrane proteins. The sequences are trimmed to a standard length from a starting key motif in conserved regions of each gene. However, despite being relatively conserved, a number of variations of this starting motif appear in the NG-MAST database (Fig. 1), causing one discordant result (Table 2). Some sequences appeared to lack a *tbpB* gene due to the presence of non-typeable *tbpB* genes acquired from *N. meningitidis*, though this was also noted in the published data. Another source of discordant results was genomes that appeared to have multiple alleles, suggesting isolate contamination or polyclonal infection.

A number of isolates were found to have novel alleles or allele combinations that were not in the most recent version of the database available at http://www.ng-mast.net. For convenience, *NGMASTER* includes an option to save these allele sequences in FASTA format for manual submission to the database and allele type assignment.

Notably, results were dependent on the accuracy and quality of the *de novo* draft genome assembly. It should be noted that for this study, draft genomes were assembled *de novo* using relatively standard parameters for *MEGAHIT* and *SPAdes* without post-assembly error checking (see Appendix 1). We were alerted to the presence of *SPAdes* assembly errors after finding the corresponding *MEGAHIT* assemblies produced different *NGMASTER* results. Concordant results were obtained for each of these genomes after identifying and correcting assembly errors through re-mapping each isolate’s sequencing reads back to the respective draft *SPAdes* assembly (see Appendix 1). These errors introduced during the *SPAdes* assembly process can also be corrected using an assembly polishing tool such as Pilon (Walker *et al.*, 2014). Assuming accurate closed genome assemblies are used with an accurate and well curated database, based on our testing, we anticipate that *NGMASTER* would produce NG-MAST results that were >99 % if not 100 % accurate.

## Conclusion

*NGMASTER* rapidly and accurately performs *in silico* NG-MAST typing of *N. gonorrhoeae* from assembled WGS data, and may be a useful command-line tool to help contextualise genomic epidemiological studies of *N. gonorrhoeae*.

## Supplementary Data

696 Supplementary File 1
