# Supplementary material for: NGMASTER: in silico multi-antigen sequence typing for Neisseria gonorrhoeae
Source: Microb Genom. 2016 Aug 25;2(8):e000076. doi: 10.1099/mgen.0.000076 (PMC5320595; doi:10.1099/mgen.0.000076)
Supplement: Supplementary file 1 [file mgen-02-76-s001.pdf]

NGMASTER – in silico Multi-Antigen Sequence Typing for *Neisseria gonorrhoeae*

Jason C Kwong[1,2,3], Anders Gonçalves da Silva[1,4], Kristin Dyet[5], Deborah A Williamson[1,4], Timothy P Stinear[1,2], Benjamin P Howden[1,2,3,4], Torsten Seemann[1,6]

[1] Doherty Applied Microbial Genomics, Doherty Institute for Infection & Immunity, Melbourne, Australia
[2] Department of Microbiology & Immunology, University of Melbourne, Parkville, Australia
[3] Department of Infectious Diseases, Austin Health, Heidelberg, Australia
[4] Microbiological Diagnostic Unit Public Health Laboratory, Doherty Institute for Infection & Immunity, Melbourne, Australia
[5] Institute of Environmental Science and Research, Wellington, New Zealand
[6] Victorian Life Sciences Computation Initiative, Carlton, Australia

Correspondence: Jason Kwong (jason.kwong@unimelb.edu.au)

Appendix

Appendix 1: List of commands and parameters used

RAW SEQUENCE TRIMMING AND ADAPTER CLIPPING

For paired-end 150 and 300 bp reads:

```
$ trimmomatic PE -phred33 R1.fastq.gz R2.fastq.gz clipped_R1.fq.gz
/dev/null clipped_R2.fq.gz /dev/null ILLUMINACLIP:NexteraPE-PE.fa:1:30:11
LEADING:20 TRAILING:20
```

For paired-end 100 bp reads:

```
$ trimmomatic PE -phred33 R1.fastq.gz R2.fastq.gz clipped_R1.fq.gz
/dev/null clipped_R2.fq.gz /dev/null ILLUMINACLIP:TruSeq2-PE.fa:1:30:11
LEADING:20 TRAILING:20
```

GENOME ASSEMBLY

MEGAHIT:

```
$ megahit --out-dir megahit -1 clipped_R1.fq.gz -2 clipped_R2.fq.gz
--min-contig-len 500 --presets bulk
```

SPAdes:

```
$ spades.py -o spades --careful -k 21,33,55,77,87,97,107,117,127
--tmp-dir /tmp -1 clipped_R1.fq.gz -2 clipped_R2.fq.gz
```

SPAdes with repeat resolution disabled:

```
$ spades.py -o spades --careful --disable-rr
-k 21,33,55,77,87,97,107,117,127 --tmp-dir /tmp
-1 clipped_R1.fq.gz -2 clipped_R2.fq.gz
```

REMAPPING READS BACK TO DRAFT ASSEMBLY
(see https://github.com/tseemann/snippy)

```
$ snippy --outdir sample --ref sample_spades.fa
--R1 sample_clipped_R1.fq.gz --R2 sample_clipped_R2.fq.gz
```

NGMASTER

```
$ ngmaster *.fa > results.txt
```

Run_Accession	STUDY	NG-MAST	por	tbpB	Megahit_ID	NG-MAST	POR	TBPB	MEGAHIT_Comments	SPAdes_ID	NG-MAST	POR	TBPB	SPAdes_Comments	Spades_before_rr_ID	NG-MAST	POR	TBPB
ERR191730	PRJEB2999	1407	908	110	ERR191730_megahit.fa	1407	908	110		ERR191730_spades.fa	1407	908	110		ERR191730_before_rr.fa	1407	908	110
ERR191731	PRJEB2999	6712	1582	4	ERR191731_megahit.fa	6712	1582	4		ERR191731_spades.fa	6712	1582	4		ERR191731_before_rr.fa	6712	1582	4
ERR191732	PRJEB2999	1407	908	110	ERR191732_megahit.fa	1407	908	110		ERR191732_spades.fa	1407	908	110		ERR191732_before_rr.fa	1407	908	110
ERR191733	PRJEB2999	Unknown	99%_Allele3745	4	ERR191733_megahit.fa	-	new	4	new	ERR191733_spades.fa	-	new	4	new	ERR191733_before_rr.fa	-	new	4
ERR191734	PRJEB2999	Unknown	99%_Allele3013	110	ERR191734_megahit.fa	-	new	110	new	ERR191734_spades.fa	-	new	110	new	ERR191734_before_rr.fa	-	new	110
ERR191735	PRJEB2999	2265	1417	4	ERR191735_megahit.fa	2265	1417	4		ERR191735_spades.fa	2265	1417	4		ERR191735_before_rr.fa	2265	1417	4
ERR191736	PRJEB2999	4986	3025	110	ERR191736_megahit.fa	4986	3025	110		ERR191736_spades.fa	4986	3025	110		ERR191736_before_rr.fa	4986	3025	110
ERR191737	PRJEB2999	New	3403	743	ERR191737_megahit.fa	-	3403	743	unassigned	ERR191737_spades.fa	-	3403	743	unassigned	ERR191737_before_rr.fa	-	3403	743
ERR191738	PRJEB2999	359	301	29	ERR191738_megahit.fa	359	301	29		ERR191738_spades.fa	359	301	29		ERR191738_before_rr.fa	359	301	29
ERR191739	PRJEB2999	Unknown	99%_Allele1986	29	ERR191739_megahit.fa	-	new	29	new	ERR191739_spades.fa	-	new	29	new	ERR191739_before_rr.fa	-	new	29
ERR191740	PRJEB2999	860	586	25	ERR191740_megahit.fa	-	586	-	missing	ERR191740_spades.fa	860	586	25		ERR191740_before_rr.fa	860	586	25
ERR191741	PRJEB2999	Unknown	97%_Allele3680	96%_Allele1456	ERR191741_megahit.fa	-	new	new	new	ERR191741_spades.fa	-	new	new	new	ERR191741_before_rr.fa	-	new	new
ERR191746	PRJEB2999	1407	908	110	ERR191746_megahit.fa	1407	908	110		ERR191746_spades.fa	1407	908	110		ERR191746_before_rr.fa	1407	908	110
ERR191747	PRJEB2999	2992	1808	29	ERR191747_megahit.fa	2992	1808	29		ERR191747_spades.fa	2992	1808	29		ERR191747_before_rr.fa	2992	1808	29
ERR191748	PRJEB2999	1407	908	110	ERR191748_megahit.fa	1407	908	110		ERR191748_spades.fa	1407	908	110		ERR191748_before_rr.fa	1407	908	110
ERR191749	PRJEB2999	286	105	21	ERR191749_megahit.fa	286	105	21		ERR191749_spades.fa	286	105	21		ERR191749_before_rr.fa	286	105	21
ERR191750	PRJEB2999	1407	908	110	ERR191750_megahit.fa	1407	908	110		ERR191750_spades.fa	1407	908	110		ERR191750_before_rr.fa	1407	908	110
ERR191751	PRJEB2999	225	4	4	ERR191751_megahit.fa	multiple	4	4/29	multiple	ERR191751_spades.fa	225	4	4		ERR191751_before_rr.fa	225	4	4
ERR191752	PRJEB2999	1405	543	110	ERR191752_megahit.fa	1405	543	110		ERR191752_spades.fa	1405	543	110		ERR191752_before_rr.fa	1405	543	110
ERR191753	PRJEB2999	1405	543	110	ERR191753_megahit.fa	1405	543	110		ERR191753_spades.fa	1405	543	110		ERR191753_before_rr.fa	1405	543	110
ERR191754	PRJEB2999	1407	908	110	ERR191754_megahit.fa	1407	908	110		ERR191754_spades.fa	1407	908	110		ERR191754_before_rr.fa	1407	908	110
ERR191755	PRJEB2999	Unknown	99%_Allele223	5	ERR191755_megahit.fa	-	new	5	new	ERR191755_spades.fa	-	new	5	new	ERR191755_before_rr.fa	-	new	5
ERR191756	PRJEB2999	1407	908	110	ERR191756_megahit.fa	1407	908	110		ERR191756_spades.fa	1407	908	110		ERR191756_before_rr.fa	1407	908	110
ERR191757	PRJEB2999	1407	908	110	ERR191757_megahit.fa	1407	908	110		ERR191757_spades.fa	1407	908	110		ERR191757_before_rr.fa	1407	908	110
ERR191758	PRJEB2999	1407	908	110	ERR191758_megahit.fa	1407	908	110		ERR191758_spades.fa	1407	908	110		ERR191758_before_rr.fa	1407	908	110
ERR191759	PRJEB2999	1407	908	110	ERR191759_megahit.fa	1407	908	110		ERR191759_spades.fa	1407	908	110		ERR191759_before_rr.fa	1407	908	110
ERR191760	PRJEB2999	1407	908	110	ERR191760_megahit.fa	1407	908	110		ERR191760_spades.fa	1407	908	110		ERR191760_before_rr.fa	1407	908	110
ERR191761	PRJEB2999	New	2329	4	ERR191761_megahit.fa	9164	2329	4	recently assigned	ERR191761_spades.fa	9164	2329	4	recently assigned	ERR191761_before_rr.fa	9164	2329	4
ERR191762	PRJEB2999	2212	1388	110	ERR191762_megahit.fa	2212	1388	110		ERR191762_spades.fa	2212	1388	110		ERR191762_before_rr.fa	2212	1388	110
ERR191763	PRJEB2999	7155	3522	29	ERR191763_megahit.fa	7155	3522	29		ERR191763_spades.fa	7155	3522	29		ERR191763_before_rr.fa	7155	3522	29
ERR191764	PRJEB2999	Unknown	99%_Allele1914	110	ERR191764_megahit.fa	-	new	110	new	ERR191764_spades.fa	-	new	110	new	ERR191764_before_rr.fa	-	new	110
ERR191765	PRJEB2999	New	1678	1169	ERR191765_megahit.fa	-	1678	1169	unassigned	ERR191765_spades.fa	-	1678	1169	unassigned	ERR191765_before_rr.fa	-	1678	1169
ERR191766	PRJEB2999	1407	908	110	ERR191766_megahit.fa	1407	908	110		ERR191766_spades.fa	1407	908	110		ERR191766_before_rr.fa	1407	908	110
ERR191767	PRJEB2999	2992	1808	29	ERR191767_megahit.fa	2992	1808	29		ERR191767_spades.fa	2992	1808	29		ERR191767_before_rr.fa	2992	1808	29
ERR191768	PRJEB2999	Unknown	99%_Allele2147	110	ERR191768_megahit.fa	-	new	110	new	ERR191768_spades.fa	-	new	110	new	ERR191768_before_rr.fa	-	new	110
ERR191769	PRJEB2999	1288	543	29	ERR191769_megahit.fa	1288	543	29		ERR191769_spades.fa	1288	543	29		ERR191769_before_rr.fa	1288	543	29
ERR191770	PRJEB2999	1407	908	110	ERR191770_megahit.fa	1407	908	110		ERR191770_spades.fa	1407	908	110		ERR191770_before_rr.fa	1407	908	110
ERR191771	PRJEB2999	437	14	4	ERR191771_megahit.fa	437	14	4		ERR191771_spades.fa	437	14	4		ERR191771_before_rr.fa	437	14	4
ERR191772	PRJEB2999	Unknown	99%_Allele2147	110	ERR191772_megahit.fa	-	new	110	new	ERR191772_spades.fa	-	new	110	new	ERR191772_before_rr.fa	-	new	110
ERR191773	PRJEB2999	Unknown	99%_Allele866	4	ERR191773_megahit.fa	-	-	4	missing	ERR191773_spades.fa	-	new	4	new	ERR191773_before_rr.fa	-	new	4
ERR191774	PRJEB2999	Unknown	99%_Allele2147	110	ERR191774_megahit.fa	-	new	110	new	ERR191774_spades.fa	-	new	110	new	ERR191774_before_rr.fa	-	new	110
ERR191775	PRJEB2999	Unknown	99%_Allele2147	110	ERR191775_megahit.fa	-	new	110	new	ERR191775_spades.fa	-	new	110	new	ERR191775_before_rr.fa	-	new	110
ERR191776	PRJEB2999	Unknown	99%_Allele2147	110	ERR191776_megahit.fa	-	new	110	new	ERR191776_spades.fa	-	new	110	new	ERR191776_before_rr.fa	-	new	110
ERR191777	PRJEB2999	Unknown	99%_Allele2147	110	ERR191777_megahit.fa	-	new	110	new	ERR191777_spades.fa	-	new	110	new	ERR191777_before_rr.fa	-	new	110
ERR191778	PRJEB2999	Unknown	99%_Allele2147	110	ERR191778_megahit.fa	-	new	110	new	ERR191778_spades.fa	-	new	110	new	ERR191778_before_rr.fa	-	new	110
ERR191779	PRJEB2999	7949	4754	16	ERR191779_megahit.fa	7949	4754	16		ERR191779_spades.fa	7949	4754	16		ERR191779_before_rr.fa	7949	4754	16
ERR191780	PRJEB2999	Unknown	99%_Allele2147	110	ERR191780_megahit.fa	-	new	110	new	ERR191780_spades.fa	-	new	110	new	ERR191780_before_rr.fa	-	new	110
ERR191781	PRJEB2999	437	14	4	ERR191781_megahit.fa	437	14	4		ERR191781_spades.fa	437	14	4		ERR191781_before_rr.fa	437	14	4
ERR191782	PRJEB2999	Unknown	99%_Allele1914	110	ERR191782_megahit.fa	-	new	110	new	ERR191782_spades.fa	-	new	110	new	ERR191782_before_rr.fa	-	new	110
ERR191783	PRJEB2999	437	14	4	ERR191783_megahit.fa	437	14	4		ERR191783_spades.fa	437	14	4		ERR191783_before_rr.fa	437	14	4
ERR191784	PRJEB2999	1407	908	110	ERR191784_megahit.fa	1407	908	110		ERR191784_spades.fa	1407	908	110		ERR191784_before_rr.fa	1407	908	110
ERR191785	PRJEB2999	3169	260	4	ERR191785_megahit.fa	3169	260	4		ERR191785_spades.fa	3169	260	4		ERR191785_before_rr.fa	3169	260	4
ERR191786	PRJEB2999	3158	1914	110	ERR191786_megahit.fa	3158	1914	110		ERR191786_spades.fa	3158	1914	110		ERR191786_before_rr.fa	3158	1914	110
ERR191787	PRJEB2999	8258	14	1514	ERR191787_megahit.fa	-	14	no_key	alternat key not detected	ERR191787_spades.fa	-	14	no_key	alternate key not detected	ERR191787_before_rr.fa	-	14	no_key
ERR191788	PRJEB2999	3709	2237	110	ERR191788_megahit.fa	3709	2237	110		ERR191788_spades.fa	3709	2237	110		ERR191788_before_rr.fa	3709	2237	110
ERR191789	PRJEB2999	New	2329	4	ERR191789_megahit.fa	9164	2329	4	recently assigned	ERR191789_spades.fa	9164	2329	4	recently assigned	ERR191789_before_rr.fa	9164	2329	4
ERR191790	PRJEB2999	3158	1914	110	ERR191790_megahit.fa	3158	1914	110		ERR191790_spades.fa	3158	1914	110		ERR191790_before_rr.fa	3158	1914	110
ERR191791	PRJEB2999	730	59	29	ERR191791_megahit.fa	730	59	29		ERR191791_spades.fa	730	59	29		ERR191791_before_rr.fa	730	59	29
ERR191792	PRJEB2999	3128	1900	110	ERR191792_megahit.fa	3128	1900	110		ERR191792_spades.fa	3128	1900	110		ERR191792_before_rr.fa	3128	1900	110
ERR191793	PRJEB2999	437	14	4	ERR191793_megahit.fa	437	14	4		ERR191793_spades.fa	437	14	4		ERR191793_before_rr.fa	437	14	4
ERR191794	PRJEB2999	3128	1900	110	ERR191794_megahit.fa	3128	1900	110		ERR191794_spades.fa	3128	1900	110		ERR191794_before_rr.fa	3128	1900	110
ERR191795	PRJEB2999	1407	908	110	ERR191795_megahit.fa	1407	908	110		ERR191795_spades.fa	1407	908	110		ERR191795_before_rr.fa	1407	908	110
ERR191796	PRJEB2999	3149	1903	110	ERR191796_megahit.fa	3149	1903	110		ERR191796_spades.fa	3149	1903	110		ERR191796_before_rr.fa	3149	1903	110
ERR191797	PRJEB2999	1407	908	110	ERR191797_megahit.fa	1407	908	110		ERR191797_spades.fa	1407	908	110		ERR191797_before_rr.fa	1407	908	110
ERR191798	PRJEB2999	Unknown	99%_Allele1914	110	ERR191798_megahit.fa	-	new	110	new	ERR191798_spades.fa	-	new	110	new	ERR191798_before_rr.fa	-	new	110
ERR191799	PRJEB2999	6734	4016	33	ERR191799_megahit.fa	6734	4016	33		ERR191799_spades.fa	6734	4016	33		ERR191799_before_rr.fa	6734	4016	33
ERR191800	PRJEB2999	5895	2700	110	ERR191800_megahit.fa	5895	2700	110		ERR191800_spades.fa	5895	2700	110		ERR191800_before_rr.fa	5895	2700	110
ERR191801	PRJEB2999	7468	19	27	ERR191801_megahit.fa	7468	19	27		ERR191801_spades.fa	7468	19	27		ERR191801_before_rr.fa	7468	19	27
ERR191802	PRJEB2999	5895	2700	110	ERR191802_megahit.fa	5895	2700	110		ERR191802_spades.fa	5895	2700	110		ERR191802_before_rr.fa	5895	2700	110
ERR191803	PRJEB2999	766	35	18	ERR191803_megahit.fa	766	35	18		ERR191803_spades.fa	766	35	18		ERR191803_before_rr.fa	766	35	18
ERR191804	PRJEB2999	1407	908	110	ERR191804_megahit.fa	1407	908	110		ERR191804_spades.fa	1407	908	110		ERR191804_before_rr.fa	1407	908	110
ERR191805	PRJEB2999	1407	908	110	ERR191805_megahit.fa	1407	908	110		ERR191805_spades.fa	1407	908	110		ERR191805_before_rr.fa	1407	908	110
ERR191806	PRJEB2999	4378	2684	110	ERR191806_megahit.fa	4378	2684	110		ERR191806_spades.fa	4378	2684	110		ERR191806_before_rr.fa	4378	2684	110
ERR191807	PRJEB2999	New	3022	28	ERR191807_megahit.fa	-	3022	28	unassigned	ERR191807_spades.fa	-	3022	28	unassigned	ERR191807_before_rr.fa	-	3022	28
ERR191808	PRJEB2999	3158	1914	110	ERR191808_megahit.fa	3158	1914	110		ERR191808_spades.fa	3158	1914	110		ERR191808_before_rr.fa	3158	1914	110
ERR191809	PRJEB2999	690	430	4	ERR191809_megahit.fa	multiple		4/110	multiple	ERR191809_spades.fa	690	430	4		ERR191809_before_rr.fa	690	430	4
ERR191810	PRJEB2999	3158	1914	110	ERR191810_megahit.fa	3158	1914	110		ERR191810_spades.fa	3158	1914	110		ERR191810_before_rr.fa	3158	1914	110
ERR191811	PRJEB2999	3449	1053	107	ERR191811_megahit.fa	3449	1053	107		ERR191811_spades.fa	3449	1053	107		ERR191811_before_rr.fa	3449	1053	107
ERR191812	PRJEB2999	3935	908	29	ERR191812_megahit.fa	3935	908	29		ERR191812_spades.fa	3935	908	29		ERR191812_before_rr.fa	3935	908	29
ERR191813	PRJEB2999	3935	908	29	ERR191813_megahit.fa	3935	908	29		ERR191813_spades.fa	3935	908	29		ERR191813_before_rr.fa	3935	908	29
ERR191814	PRJEB2999	4533	2763	110	ERR191814_megahit.fa	4533	2763	110		ERR191814_spades.fa	4533	2763	110		ERR191814_before_rr.fa	4533	2763	110
ERR191815	PRJEB2999	1440	105	4	ERR191815_megahit.fa	multiple		4/29	multiple	ERR191815_spades.fa	1440	105	4		ERR191815_before_rr.fa	1440	105	4
ERR191816	PRJEB2999	3431	2078	110	ERR191816_megahit.fa	3431	2078	110		ERR191816_spades.fa	3431	2078	110		ERR191816_before_rr.fa	3431	2078	110
ERR191817	PRJEB2999	3431	2078	110	ERR191817_megahit.fa	3431	2078	110		ERR191817_spades.fa	3431	2078	110		ERR191817_before_rr.fa	3431	2078	110
ERR191818	PRJEB2999	1407	908	110	ERR191818_megahit.fa	1407	908	110		ERR191818_spades.fa	1407	908	110		ERR191818_before_rr.fa	1407	908	110
ERR191819	PRJEB2999	4205	2583	743	ERR191819_megahit.fa	4205	2583	743		ERR191819_spades.fa	4205	2583	743		ERR191819_before_rr.fa	4205	2583	743
ERR191820	PRJEB2999	Unknown	99%_Allele3013	110	ERR191820_megahit.fa	-	new	110	new	ERR191820_spades.fa	-	new	110	new	ERR191820_before_rr.fa	-	new	110
ERR191821	PRJEB2999	730	59	29	ERR191821_megahit.fa	730	59	29		ERR191821_spades.fa	730	59	29		ERR191821_before_rr.fa	730	59	29
ERR191822	PRJEB2999	Unknown	99%_Allele3013	110	ERR191822_megahit.fa	-	new	110	new	ERR191822_spades.fa	-	new	110	new	ERR191822_before_rr.fa	-	new	110
ERR191823	PRJEB2999	Unknown	99%_Allele1582	4	ERR191823_megahit.fa	-	new	4	new	ERR191823_spades.fa	-	new	4	new	ERR191823_before_rr.fa	-	new	4
ERR191824	PRJEB2999	1407	908	110	ERR191824_megahit.fa	1407	908	110		ERR191824_spades.fa	1407	908	110		ERR191824_before_rr.fa	1407	908	110

ERR191825	PRJEB2999	2992	1808	29	ERR191825_megahit.fa	2992	1808	29		ERR191825_spades.fa	2992	1808	29		ERR191825_before_rr.fa	2992	1808	29
ERR223603	PRJEB2999	1407	908	110	ERR223603_megahit.fa	1407	908	110		ERR223603_spades.fa	1407	908	110		ERR223603_before_rr.fa	1407	908	110
ERR223604	PRJEB2999	2992	1808	29	ERR223604_megahit.fa	2992	1808	29		ERR223604_spades.fa	2992	1808	29		ERR223604_before_rr.fa	2992	1808	29
ERR223605	PRJEB2999	437	14	4	ERR223605_megahit.fa	437	14	4		ERR223605_spades.fa	437	14	4		ERR223605_before_rr.fa	437	14	4
ERR223606	PRJEB2999	Unknown	97%_Allele3680	96%_Allele1456	ERR223606_megahit.fa	-	new	new	new	ERR223606_spades.fa	-	new	new	new	ERR223606_before_rr.fa	-	new	new
ERR223607	PRJEB2999	1407	908	110	ERR223607_megahit.fa	1407	908	110		ERR223607_spades.fa	1407	908	110		ERR223607_before_rr.fa	1407	908	110
ERR223608	PRJEB2999	4951	3003	110	ERR223608_megahit.fa	4951	3003	110		ERR223608_spades.fa	4951	3003	110		ERR223608_before_rr.fa	4951	3003	110
ERR223609	PRJEB2999	1407	908	110	ERR223609_megahit.fa	1407	908	110		ERR223609_spades.fa	1407	908	110		ERR223609_before_rr.fa	1407	908	110
ERR223610	PRJEB2999	1407	908	110	ERR223610_megahit.fa	1407	908	110		ERR223610_spades.fa	1407	908	110		ERR223610_before_rr.fa	1407	908	110
ERR223611	PRJEB2999	4269	2623	110	ERR223611_megahit.fa	4269	2623	110		ERR223611_spades.fa	4269	2623	110		ERR223611_before_rr.fa	4269	2623	110
ERR223612	PRJEB2999	359	301	29	ERR223612_megahit.fa	359	301	29		ERR223612_spades.fa	359	301	29		ERR223612_before_rr.fa	359	301	29
ERR223613	PRJEB2999	1407	908	110	ERR223613_megahit.fa	1407	908	110		ERR223613_spades.fa	1407	908	110		ERR223613_before_rr.fa	1407	908	110
ERR223614	PRJEB2999	1978	1259	4	ERR223614_megahit.fa	1978	1259	4		ERR223614_spades.fa	1978	1259	4		ERR223614_before_rr.fa	1978	1259	4
ERR223615	PRJEB2999	1407	908	110	ERR223615_megahit.fa	multiple		110/4	multiple	ERR223615_spades.fa	multiple	908	4/110	nant allele (higher coverage, assembled in context; allele 4 lo	ERR223615_before_rr.fa	1407	908	110
ERR223616	PRJEB2999	2992	1808	29	ERR223616_megahit.fa	2992	1808	29		ERR223616_spades.fa	2992	1808	29		ERR223616_before_rr.fa	2992	1808	29
ERR223619	PRJEB2999	Unknown	99%_Allele4576	110	ERR223619_megahit.fa	-	new	110	new	ERR223619_spades.fa	-	new	110	new	ERR223619_before_rr.fa	-	new	110
ERR223620	PRJEB2999	1407	908	110	ERR223620_megahit.fa	1407	908	110		ERR223620_spades.fa	1407	908	110		ERR223620_before_rr.fa	1407	908	110
ERR223621	PRJEB2999	1407	908	110	ERR223621_megahit.fa	1407	908	110		ERR223621_spades.fa	1407	908	110		ERR223621_before_rr.fa	1407	908	110
ERR223622	PRJEB2999	225	4	4	ERR223622_megahit.fa	225	4	4		ERR223622_spades.fa	225	4	4		ERR223622_before_rr.fa	225	4	4
ERR223623	PRJEB2999	1407	908	110	ERR223623_megahit.fa	1407	908	110		ERR223623_spades.fa	1407	908	110		ERR223623_before_rr.fa	1407	908	110
ERR223624	PRJEB2999	Unknown	99%_Allele1808	29	ERR223624_megahit.fa	-	new	29	new	ERR223624_spades.fa	-	new	29	new	ERR223624_before_rr.fa	-	new	29
ERR223625	PRJEB2999	1407	908	110	ERR223625_megahit.fa	1407	908	110		ERR223625_spades.fa	1407	908	110		ERR223625_before_rr.fa	1407	908	110
ERR223626	PRJEB2999	4198	2577	29	ERR223626_megahit.fa	4198	2577	29		ERR223626_spades.fa	4198	2577	29		ERR223626_before_rr.fa	4198	2577	29
ERR223627	PRJEB2999	1407	908	110	ERR223627_megahit.fa	1407	908	110		ERR223627_spades.fa	1407	908	110		ERR223627_before_rr.fa	1407	908	110
ERR223628	PRJEB2999	1978	1259	4	ERR223628_megahit.fa	1978	1259	4		ERR223628_spades.fa	1978	1259	4		ERR223628_before_rr.fa	1978	1259	4
ERR223629	PRJEB2999	1407	908	110	ERR223629_megahit.fa	1407	908	110		ERR223629_spades.fa	1407	908	110		ERR223629_before_rr.fa	1407	908	110
ERR223630	PRJEB2999	1407	908	110	ERR223630_megahit.fa	1407	908	110		ERR223630_spades.fa	1407	908	110		ERR223630_before_rr.fa	1407	908	110
ERR223631	PRJEB2999	1407	908	110	ERR223631_megahit.fa	1407	908	110		ERR223631_spades.fa	1407	908	110		ERR223631_before_rr.fa	1407	908	110
ERR223632	PRJEB2999	2992	1808	29	ERR223632_megahit.fa	2992	1808	29		ERR223632_spades.fa	2992	1808	29		ERR223632_before_rr.fa	2992	1808	29
ERR223633	PRJEB2999	Unknown	98%_Allele875	110	ERR223633_megahit.fa	-	new	110	new	ERR223633_spades.fa	-	new	110	new	ERR223633_before_rr.fa	-	new	110
ERR223634	PRJEB2999	2992	1808	29	ERR223634_megahit.fa	2992	1808	29		ERR223634_spades.fa	2992	1808	29		ERR223634_before_rr.fa	2992	1808	29
ERR223635	PRJEB2999	1407	908	110	ERR223635_megahit.fa	1407	908	110		ERR223635_spades.fa	1407	908	110		ERR223635_before_rr.fa	1407	908	110
ERR223636	PRJEB2999	1978	1259	4	ERR223636_megahit.fa	1978	1259	4		ERR223636_spades.fa	1978	1259	4		ERR223636_before_rr.fa	1978	1259	4
ERR223637	PRJEB2999	1407	908	110	ERR223637_megahit.fa	1407	908	110		ERR223637_spades.fa	1407	908	110		ERR223637_before_rr.fa	1407	908	110
ERR223638	PRJEB2999	437	14	4	ERR223638_megahit.fa	437	14	4		ERR223638_spades.fa	437	14	4		ERR223638_before_rr.fa	437	14	4
ERR223639	PRJEB2999	1407	908	110	ERR223639_megahit.fa	1407	908	110		ERR223639_spades.fa	1407	908	110		ERR223639_before_rr.fa	1407	908	110
ERR223640	PRJEB2999	437	14	4	ERR223640_megahit.fa	437	14	4		ERR223640_spades.fa	437	14	4		ERR223640_before_rr.fa	437	14	4
ERR223641	PRJEB2999	1407	908	110	ERR223641_megahit.fa	1407	908	110		ERR223641_spades.fa	1407	908	110		ERR223641_before_rr.fa	1407	908	110
ERR223642	PRJEB2999	Unknown	99%_Allele3523	29	ERR223642_megahit.fa	-	new	29	new	ERR223642_spades.fa	-	new	29	new	ERR223642_before_rr.fa	-	new	29
ERR223643	PRJEB2999	1407	908	110	ERR223643_megahit.fa	1407	908	110		ERR223643_spades.fa	1407	908	110		ERR223643_before_rr.fa	1407	908	110
ERR223644	PRJEB2999	2992	1808	29	ERR223644_megahit.fa	2992	1808	29		ERR223644_spades.fa	2992	1808	29		ERR223644_before_rr.fa	2992	1808	29
ERR223645	PRJEB2999	1407	908	110	ERR223645_megahit.fa	1407	908	110		ERR223645_spades.fa	1407	908	110		ERR223645_before_rr.fa	1407	908	110
ERR223646	PRJEB2999	1978	1259	4	ERR223646_megahit.fa	-	-	4	missing	ERR223646_spades.fa	1978	1259	4		ERR223646_before_rr.fa	1978	1259	4
ERR223647	PRJEB2999	Unknown	99%_Allele2147	110	ERR223647_megahit.fa	-	new	110	new	ERR223647_spades.fa	-	new	110	new	ERR223647_before_rr.fa	-	new	110
ERR223648	PRJEB2999	3485	760	33	ERR223648_megahit.fa	3485	760	33		ERR223648_spades.fa	3485	760	33		ERR223648_before_rr.fa	3485	760	33
ERR223649	PRJEB2999	Unknown	99%_Allele2147	110	ERR223649_megahit.fa	-	new	110	new	ERR223649_spades.fa	-	new	110	new	ERR223649_before_rr.fa	-	new	110
ERR223650	PRJEB2999	Unknown	99%_Allele1986	29	ERR223650_megahit.fa	-	new	29	new	ERR223650_spades.fa	-	new	29	new	ERR223650_before_rr.fa	-	new	29
ERR223651	PRJEB2999	1407	908	110	ERR223651_megahit.fa	1407	908	110		ERR223651_spades.fa	1407	908	110		ERR223651_before_rr.fa	1407	908	110
ERR223652	PRJEB2999	3307	30	743	ERR223652_megahit.fa	3307	30	743		ERR223652_spades.fa	3307	30	743		ERR223652_before_rr.fa	3307	30	743
ERR223653	PRJEB2999	Unknown	99%_Allele2147	110	ERR223653_megahit.fa	-	new	110	new	ERR223653_spades.fa	-	new	110	new	ERR223653_before_rr.fa	-	new	110
ERR223654	PRJEB2999	1440	105	4	ERR223654_megahit.fa	1440	105	4		ERR223654_spades.fa	1440	105	4		ERR223654_before_rr.fa	1440	105	4
ERR223655	PRJEB2999	5895	2700	110	ERR223655_megahit.fa	5895	2700	110		ERR223655_spades.fa	5895	2700	110		ERR223655_before_rr.fa	5895	2700	110
ERR223656	PRJEB2999	7132	4270	129	ERR223656_megahit.fa	7132	4270	129		ERR223656_spades.fa	7132	4270	129		ERR223656_before_rr.fa	7132	4270	129
ERR223657	PRJEB2999	1407	908	110	ERR223657_megahit.fa	1407	908	110		ERR223657_spades.fa	1407	908	110		ERR223657_before_rr.fa	1407	908	110
ERR223658	PRJEB2999	1791	543	186	ERR223658_megahit.fa	1791	543	186		ERR223658_spades.fa	1791	543	186		ERR223658_before_rr.fa	1791	543	186
ERR223659	PRJEB2999	5895	2700	110	ERR223659_megahit.fa	5895	2700	110		ERR223659_spades.fa	5895	2700	110		ERR223659_before_rr.fa	5895	2700	110
ERR223660	PRJEB2999	758	90	29	ERR223660_megahit.fa	758	90	29		ERR223660_spades.fa	758	90	29		ERR223660_before_rr.fa	758	90	29
ERR223661	PRJEB2999	1407	908	110	ERR223661_megahit.fa	1407	908	110		ERR223661_spades.fa	1407	908	110		ERR223661_before_rr.fa	1407	908	110
ERR223662	PRJEB2999	Unknown	99%_Allele4354	29	ERR223662_megahit.fa	9247	5505	29	recently assigned	ERR223662_spades.fa	9247	5505	29	new	ERR223662_before_rr.fa	9247	5505	29
ERR223663	PRJEB2999	1407	908	110	ERR223663_megahit.fa	1407	908	110		ERR223663_spades.fa	1407	908	110		ERR223663_before_rr.fa	1407	908	110
ERR223664	PRJEB2999	437	14	4	ERR223664_megahit.fa	437	14	4		ERR223664_spades.fa	437	14	4		ERR223664_before_rr.fa	437	14	4
ERR223665	PRJEB2999	1407	908	110	ERR223665_megahit.fa	1407	908	110		ERR223665_spades.fa	1407	908	110		ERR223665_before_rr.fa	1407	908	110
ERR223666	PRJEB2999	1407	908	110	ERR223666_megahit.fa	1407	908	110		ERR223666_spades.fa	1407	908	110		ERR223666_before_rr.fa	1407	908	110
ERR223667	PRJEB2999	5895	2700	110	ERR223667_megahit.fa	5895	2700	110		ERR223667_spades.fa	5895	2700	110		ERR223667_before_rr.fa	5895	2700	110
ERR223668	PRJEB2999	5268	19	25	ERR223668_megahit.fa	5268	19	25		ERR223668_spades.fa	5268	19	25		ERR223668_before_rr.fa	5268	19	25
ERR223669	PRJEB2999	3431	2078	110	ERR223669_megahit.fa	3431	2078	110		ERR223669_spades.fa	3431	2078	110		ERR223669_before_rr.fa	3431	2078	110
ERR223670	PRJEB2999	2493	4	29	ERR223670_megahit.fa	2493	4	29		ERR223670_spades.fa	2493	4	29		ERR223670_before_rr.fa	2493	4	29
ERR223671	PRJEB2999	1407	908	110	ERR223671_megahit.fa	1407	908	110		ERR223671_spades.fa	1407	908	110		ERR223671_before_rr.fa	1407	908	110
ERR223672	PRJEB2999	1407	908	110	ERR223672_megahit.fa	1407	908	110		ERR223672_spades.fa	1407	908	110		ERR223672_before_rr.fa	1407	908	110
ERR223673	PRJEB2999	Unknown	2700	alt_tpbB	ERR223673_megahit.fa	-	2700	-	alternate tbpB from N. meningitidis	ERR223673_spades.fa	-	2700	-	alternate tbpB from N. meningitidis	ERR223673_before_rr.fa	-	2700	-
ERR223674	PRJEB2999	225	4	4	ERR223674_megahit.fa	225	4	4		ERR223674_spades.fa	225	4	4		ERR223674_before_rr.fa	225	4	4
ERR223675	PRJEB2999	Unknown	2700	alt_tpbB	ERR223675_megahit.fa	-	2700	-	alternate tbpB from N. meningitidis	ERR223675_spades.fa	-	2700	-	alternate tbpB from N. meningitidis	ERR223675_before_rr.fa	-	2700	-
ERR223676	PRJEB2999	225	4	4	ERR223676_megahit.fa	225	4	4		ERR223676_spades.fa	225	4	4		ERR223676_before_rr.fa	225	4	4
ERR223677	PRJEB2999	Unknown	2700	alt_tpbB	ERR223677_megahit.fa	-	2700	-	alternate tbpB from N. meningitidis	ERR223677_spades.fa	-	2700	-	alternate tbpB from N. meningitidis	ERR223677_before_rr.fa	-	2700	-
ERR223678	PRJEB2999	6306	3716	25	ERR223678_megahit.fa	6306	3716	25		ERR223678_spades.fa	6306	3716	25		ERR223678_before_rr.fa	6306	3716	25
ERR223679	PRJEB2999	Unknown	2700	alt_tpbB	ERR223679_megahit.fa	-	2700	-	alternate tbpB from N. meningitidis	ERR223679_spades.fa	-	2700	-	alternate tbpB from N. meningitidis	ERR223679_before_rr.fa	-	2700	-
ERR223680	PRJEB2999	2992	1808	29	ERR223680_megahit.fa	2992	1808	29		ERR223680_spades.fa	2992	1808	29		ERR223680_before_rr.fa	2992	1808	29
ERR223681	PRJEB2999	Unknown	2700	alt_tpbB	ERR223681_megahit.fa	-	2700	-	alternate tbpB from N. meningitidis	ERR223681_spades.fa	-	2700	-	alternate tbpB from N. meningitidis	ERR223681_before_rr.fa	-	2700	-
ERR223682	PRJEB2999	Unknown	99%_Allele3381	29	ERR223682_megahit.fa	-	new	-	missing	ERR223682_spades.fa	-	new	29	new	ERR223682_before_rr.fa	-	new	29
ERR223683	PRJEB2999	New	7	110	ERR223683_megahit.fa	-	7	110	unassigned	ERR223683_spades.fa	-	7	110	unassigned	ERR223683_before_rr.fa	-	7	110
ERR223684	PRJEB2999	1407	908	110	ERR223684_megahit.fa	1407	908	110		ERR223684_spades.fa	1407	908	110		ERR223684_before_rr.fa	1407	908	110
ERR223685	PRJEB2999	3128	1900	110	ERR223685_megahit.fa	3128	1900	110		ERR223685_spades.fa	3128	1900	110		ERR223685_before_rr.fa	3128	1900	110
ERR223686	PRJEB2999	New	5014	743	ERR223686_megahit.fa	-	5014	743	unassigned	ERR223686_spades.fa	-	5014	743	unassigned	ERR223686_before_rr.fa	-	5014	743
ERR223687	PRJEB2999	3128	1900	110	ERR223687_megahit.fa	3128	1900	110		ERR223687_spades.fa	3128	1900	110		ERR223687_before_rr.fa	3128	1900	110
ERR223688	PRJEB2999	286	105	21	ERR223688_megahit.fa	multiple	105	110/21	multiple	ERR223688_spades.fa	286	105	21		ERR223688_before_rr.fa	286	105	21
ERR223689	PRJEB2999	3128	1900	110	ERR223689_megahit.fa	3128	1900	110		ERR223689_spades.fa	3128	1900	110		ERR223689_before_rr.fa	3128	1900	110
ERR223690	PRJEB2999	4424	35	944	ERR223690_megahit.fa	4424	35	944		ERR223690_spades.fa	4424	35	944		ERR223690_before_rr.fa	4424	35	944
ERR223691	PRJEB2999	3128	1900	110	ERR223691_megahit.fa	3128	1900	110		ERR223691_spades.fa	3128	1900	110		ERR223691_before_rr.fa	3128	1900	110
ERR223692	PRJEB2999	New	997	743	ERR223692_megahit.fa	-	997	743	unassigned	ERR223692_spades.fa	-	997	743	unassigned	ERR223692_before_rr.fa	-	997	743
ERR223693	PRJEB2999	3128	1900	110	ERR223693_megahit.fa	3128	1900	110		ERR223693_spades.fa	3128	1900	110		ERR223693_before_rr.fa	3128	1900	110
ERR223694	PRJEB2999	New	1142	743	ERR223694_megahit.fa	9271	1142	743	recently assigned	ERR223694_spades.fa	9271	1142	743	recently assigned	ERR223694_before_rr.fa	9271	1142	743
ERR223695	PRJEB2999	4556	2780	110	ERR223695_megahit.fa	4556	2780	110		ERR223695_spades.fa	4556	2780	110		ERR223695_before_rr.fa	4556	2780	110

ERR223696	PRJEB2999	1861	4	135	ERR223696_megahit.fa	1861	4	135		ERR223696_spades.fa	1861	4	135		ERR223696_before_rr.fa	1861	4	135
ERR223697	PRJEB2999	Unknown	2700	alt_tpbB	ERR223697_megahit.fa	-	2700	-	alternate tbpB from N. meningitidis	ERR223697_spades.fa	-	2700	-	alternate tbpB from N. meningitidis	ERR223697_before_rr.fa	-	2700	-
ERR223698	PRJEB2999	2992	1808	29	ERR223698_megahit.fa	2992	1808	29		ERR223698_spades.fa	2992	1808	29		ERR223698_before_rr.fa	2992	1808	29
NCCP11945	PRJNA29335	3618	1284	4	NCCP11945.fna	10699	6277	4	checked with BLAST - 100% match to tbpB-6277	NCCP11945.fna	10699	6277	4	checked with BLAST - 100% match to tbpB-6277	NCCP11945.fna	10699	6277	4
SRR1661153	PRJNA266539	126	206	380	SRR1661153_megahit.fa	380	206	126	transcription error in published result	SRR1661153_spades.fa	380	206	126	transcription error in published result	SRR1661153_before_rr.fa	380	206	126
SRR1661154	PRJNA266539	10443	4	513	SRR1661154_megahit.fa	10443	4	513		SRR1661154_spades.fa	10443	4	513		SRR1661154_before_rr.fa	10443	4	513
SRR1661155	PRJNA266539	21	14	33	SRR1661155_megahit.fa	21	14	33		SRR1661155_spades.fa	21	14	33		SRR1661155_before_rr.fa	21	14	33
SRR1661156	PRJNA266539	10476	6124	1850	SRR1661156_megahit.fa	-	6124	-	missing	SRR1661156_spades.fa	10476	6124	1850		SRR1661156_before_rr.fa	10476	6124	1850
SRR1661157	PRJNA266539	10469	4637	22	SRR1661157_megahit.fa	10469	4637	22		SRR1661157_spades.fa	10469	4637	22		SRR1661157_before_rr.fa	10469	4637	22
SRR1661158	PRJNA266539	4138	2528	10	SRR1661158_megahit.fa	-	-	-	missing	SRR1661158_spades.fa	4138	2528	10		SRR1661158_before_rr.fa	4138	2528	10
SRR1661159	PRJNA266539	8	7	3	SRR1661159_megahit.fa	8	7	3		SRR1661159_spades.fa	8	7	3		SRR1661159_before_rr.fa	8	7	3
SRR1661160	PRJNA266539	2533	101	3	SRR1661160_megahit.fa	2533	101	3		SRR1661160_spades.fa	-	101	new	spades assembly error	SRR1661160_before_rr.fa	2533	101	3
SRR1661161	PRJNA266539	4383	444	21	SRR1661161_megahit.fa	4383	444	21		SRR1661161_spades.fa	4383	444	21		SRR1661161_before_rr.fa	4383	444	21
SRR1661162	PRJNA266539	51	39	27	SRR1661162_megahit.fa	51	39	27		SRR1661162_spades.fa	51	39	27		SRR1661162_before_rr.fa	51	39	27
SRR1661163	PRJNA266539	108	100	24	SRR1661163_megahit.fa	-	-	24	missing	SRR1661163_spades.fa	108	100	24		SRR1661163_before_rr.fa	108	100	24
SRR1661164	PRJNA266539	1424	917	10	SRR1661164_megahit.fa	-	-	10	missing	SRR1661164_spades.fa	1424	917	10		SRR1661164_before_rr.fa	1424	917	10
SRR1661165	PRJNA266539	84	69	33	SRR1661165_megahit.fa	84	69	33		SRR1661165_spades.fa	84	69	33		SRR1661165_before_rr.fa	84	69	33
SRR1661166	PRJNA266539	4383	444	21	SRR1661166_megahit.fa	4383	444	21		SRR1661166_spades.fa	4383	444	21		SRR1661166_before_rr.fa	4383	444	21
SRR1661167	PRJNA266539	5	4	33	SRR1661167_megahit.fa	-	4	-	missing	SRR1661167_spades.fa	5	4	33		SRR1661167_before_rr.fa	5	4	33
SRR1661168	PRJNA266539	4437	2727	10	SRR1661168_megahit.fa	4437	2727	10		SRR1661168_spades.fa	4437	2727	10		SRR1661168_before_rr.fa	4437	2727	10
SRR1661169	PRJNA266539	9022	5342	171	SRR1661169_megahit.fa	9022	5342	171		SRR1661169_spades.fa	9022	5342	171		SRR1661169_before_rr.fa	9022	5342	171
SRR1661170	PRJNA266539	757	262	4	SRR1661170_megahit.fa	757	262	4		SRR1661170_spades.fa	757	262	4		SRR1661170_before_rr.fa	757	262	4
SRR1661171	PRJNA266539	25	18	27	SRR1661171_megahit.fa	25	18	27		SRR1661171_spades.fa	25	18	27		SRR1661171_before_rr.fa	25	18	27
SRR1661172	PRJNA266539	210	59	4	SRR1661172_megahit.fa	210	59	4		SRR1661172_spades.fa	210	59	4		SRR1661172_before_rr.fa	210	59	4
SRR1661173	PRJNA266539	1407	908	110	SRR1661173_megahit.fa	1407	908	110		SRR1661173_spades.fa	1407	908	110		SRR1661173_before_rr.fa	1407	908	110
SRR1661174	PRJNA266539	225	4	4	SRR1661174_megahit.fa	225	4	4		SRR1661174_spades.fa	225	4	4		SRR1661174_before_rr.fa	225	4	4
SRR1661175	PRJNA266539	1407	908	110	SRR1661175_megahit.fa	1407	908	110		SRR1661175_spades.fa	1407	908	110		SRR1661175_before_rr.fa	1407	908	110
SRR1661176	PRJNA266539	1407	908	110	SRR1661176_megahit.fa	1407	908	110		SRR1661176_spades.fa	1407	908	110		SRR1661176_before_rr.fa	1407	908	110
SRR1661177	PRJNA266539	69	55	4	SRR1661177_megahit.fa	69	55	4		SRR1661177_spades.fa	69	55	4		SRR1661177_before_rr.fa	69	55	4
SRR1661178	PRJNA266539	545	63	171	SRR1661178_megahit.fa	545	63	171		SRR1661178_spades.fa	545	63	171		SRR1661178_before_rr.fa	545	63	171
SRR1661179	PRJNA266539	1056	609	186	SRR1661179_megahit.fa	1056	609	186		SRR1661179_spades.fa	1056	609	186		SRR1661179_before_rr.fa	1056	609	186
SRR1661180	PRJNA266539	2	2	16	SRR1661180_megahit.fa	2	2	16		SRR1661180_spades.fa	2	2	16		SRR1661180_before_rr.fa	2	2	16
SRR1661181	PRJNA266539	4014	206	241	SRR1661181_megahit.fa	4014	206	241		SRR1661181_spades.fa	4014	206	241		SRR1661181_before_rr.fa	4014	206	241
SRR1661182	PRJNA266539	4014	206	241	SRR1661182_megahit.fa	-	-	241	missing	SRR1661182_spades.fa	4014	206	241		SRR1661182_before_rr.fa	4014	206	241
SRR1661183	PRJNA266539	225	4	4	SRR1661183_megahit.fa	225	4	4		SRR1661183_spades.fa	225	4	4		SRR1661183_before_rr.fa	225	4	4
SRR1661184	PRJNA266539	3108	1884	4	SRR1661184_megahit.fa	3108	1884	4		SRR1661184_spades.fa	3108	1884	4		SRR1661184_before_rr.fa	3108	1884	4
SRR1661185	PRJNA266539	225	4	4	SRR1661185_megahit.fa	-	-	4	missing	SRR1661185_spades.fa	225	4	4		SRR1661185_before_rr.fa	225	4	4
SRR1661186	PRJNA266539	3108	1884	4	SRR1661186_megahit.fa	3108	1884	4		SRR1661186_spades.fa	3108	1884	4		SRR1661186_before_rr.fa	3108	1884	4
SRR1661187	PRJNA266539	1407	908	110	SRR1661187_megahit.fa	-	908	-	missing	SRR1661187_spades.fa	1407	908	110		SRR1661187_before_rr.fa	1407	908	110
SRR1661188	PRJNA266539	225	4	4	SRR1661188_megahit.fa	225	4	4		SRR1661188_spades.fa	225	4	4		SRR1661188_before_rr.fa	225	4	4
SRR1661189	PRJNA266539	3108	1884	4	SRR1661189_megahit.fa	3108	1884	4		SRR1661189_spades.fa	3108	1884	4		SRR1661189_before_rr.fa	3108	1884	4
SRR1661190	PRJNA266539	1409	910	4	SRR1661190_megahit.fa	-	-	-	missing	SRR1661190_spades.fa	1409	910	4		SRR1661190_before_rr.fa	1409	910	4
SRR1661191	PRJNA266539	225	4	4	SRR1661191_megahit.fa	225	4	4		SRR1661191_spades.fa	225	4	4		SRR1661191_before_rr.fa	225	4	4
SRR1661192	PRJNA266539	225	4	4	SRR1661192_megahit.fa	225	4	4		SRR1661192_spades.fa	225	4	4		SRR1661192_before_rr.fa	225	4	4
SRR1661193	PRJNA266539	225	4	4	SRR1661193_megahit.fa	225	4	4		SRR1661193_spades.fa	225	4	4		SRR1661193_before_rr.fa	225	4	4
SRR1661194	PRJNA266539	3565	2159	25	SRR1661194_megahit.fa	3565	2159	25		SRR1661194_spades.fa	3565	2159	25		SRR1661194_before_rr.fa	3565	2159	25
SRR1661195	PRJNA266539	4427	2694	10	SRR1661195_megahit.fa	4427	2694	10		SRR1661195_spades.fa	4427	2694	10		SRR1661195_before_rr.fa	4427	2694	10
SRR1661196	PRJNA266539	1407	908	110	SRR1661196_megahit.fa	1407	908	110		SRR1661196_spades.fa	1407	908	110		SRR1661196_before_rr.fa	1407	908	110
SRR1661197	PRJNA266539	1407	908	110	SRR1661197_megahit.fa	1407	908	110		SRR1661197_spades.fa	1407	908	110		SRR1661197_before_rr.fa	1407	908	110
SRR1661198	PRJNA266539	1407	908	110	SRR1661198_megahit.fa	1407	908	110		SRR1661198_spades.fa	1407	908	110		SRR1661198_before_rr.fa	1407	908	110
SRR1661199	PRJNA266539	3108	1884	4	SRR1661199_megahit.fa	3108	1884	4		SRR1661199_spades.fa	3108	1884	4		SRR1661199_before_rr.fa	3108	1884	4
SRR1661200	PRJNA266539	1407	908	110	SRR1661200_megahit.fa	1407	908	110		SRR1661200_spades.fa	1407	908	110		SRR1661200_before_rr.fa	1407	908	110
SRR1661201	PRJNA266539	3158	1914	110	SRR1661201_megahit.fa	-	-	110	missing	SRR1661201_spades.fa	3158	1914	110		SRR1661201_before_rr.fa	3158	1914	110
SRR1661202	PRJNA266539	3779	2147	110	SRR1661202_megahit.fa	3779	2147	110		SRR1661202_spades.fa	3779	2147	110		SRR1661202_before_rr.fa	3779	2147	110
SRR1661203	PRJNA266539	3158	1914	110	SRR1661203_megahit.fa	-	-	110	missing	SRR1661203_spades.fa	3158	1914	110		SRR1661203_before_rr.fa	3158	1914	110
SRR1661204	PRJNA266539	225	4	4	SRR1661204_megahit.fa	225	4	4		SRR1661204_spades.fa	225	4	4		SRR1661204_before_rr.fa	225	4	4
SRR1661205	PRJNA266539	225	4	4	SRR1661205_megahit.fa	-	-	4	missing	SRR1661205_spades.fa	225	4	4		SRR1661205_before_rr.fa	225	4	4
SRR1661206	PRJNA266539	3158	1914	110	SRR1661206_megahit.fa	3158	1914	110		SRR1661206_spades.fa	3158	1914	110		SRR1661206_before_rr.fa	3158	1914	110
SRR1661207	PRJNA266539	1948	543	479	SRR1661207_megahit.fa	1948	543	479		SRR1661207_spades.fa	1948	543	479		SRR1661207_before_rr.fa	1948	543	479
SRR1661208	PRJNA266539	1407	908	110	SRR1661208_megahit.fa	1407	908	110		SRR1661208_spades.fa	1407	908	110		SRR1661208_before_rr.fa	1407	908	110
SRR1661209	PRJNA266539	1407	908	110	SRR1661209_megahit.fa	1407	908	110		SRR1661209_spades.fa	1407	908	110		SRR1661209_before_rr.fa	1407	908	110
SRR1661210	PRJNA266539	1407	908	110	SRR1661210_megahit.fa	1407	908	110		SRR1661210_spades.fa	1407	908	110		SRR1661210_before_rr.fa	1407	908	110
SRR1661211	PRJNA266539	2992	1808	29	SRR1661211_megahit.fa	2992	1808	29		SRR1661211_spades.fa	2992	1808	29		SRR1661211_before_rr.fa	2992	1808	29
SRR1661212	PRJNA266539	1407	908	110	SRR1661212_megahit.fa	1407	908	110		SRR1661212_spades.fa	1407	908	110		SRR1661212_before_rr.fa	1407	908	110
SRR1661213	PRJNA266539	1407	908	110	SRR1661213_megahit.fa	1407	908	110		SRR1661213_spades.fa	1407	908	110		SRR1661213_before_rr.fa	1407	908	110
SRR1661214	PRJNA266539	225	4	4	SRR1661214_megahit.fa	-	-	4	missing	SRR1661214_spades.fa	225	4	4		SRR1661214_before_rr.fa	225	4	4
SRR1661215	PRJNA266539	3149	1903	110	SRR1661215_megahit.fa	3149	1903	110		SRR1661215_spades.fa	3149	1903	110		SRR1661215_before_rr.fa	3149	1903	110
SRR1661216	PRJNA266539	1407	908	110	SRR1661216_megahit.fa	1407	908	110		SRR1661216_spades.fa	1407	908	110		SRR1661216_before_rr.fa	1407	908	110
SRR1661217	PRJNA266539	1407	908	110	SRR1661217_megahit.fa	1407	908	110		SRR1661217_spades.fa	1407	908	110		SRR1661217_before_rr.fa	1407	908	110
SRR1661218	PRJNA266539	2992	1808	29	SRR1661218_megahit.fa	2992	1808	29		SRR1661218_spades.fa	2992	1808	29		SRR1661218_before_rr.fa	2992	1808	29
SRR1661219	PRJNA266539	25	18	27	SRR1661219_megahit.fa	25	18	27		SRR1661219_spades.fa	25	18	27		SRR1661219_before_rr.fa	25	18	27
SRR1661220	PRJNA266539	1407	908	110	SRR1661220_megahit.fa	1407	908	110		SRR1661220_spades.fa	1407	908	110		SRR1661220_before_rr.fa	1407	908	110
SRR1661221	PRJNA266539	1978	1259	4	SRR1661221_megahit.fa	1978	1259	4		SRR1661221_spades.fa	1978	1259	4		SRR1661221_before_rr.fa	1978	1259	4
SRR1661222	PRJNA266539	3550	4	49	SRR1661222_megahit.fa	3550	4	49		SRR1661222_spades.fa	3550	4	49		SRR1661222_before_rr.fa	3550	4	49
SRR1661223	PRJNA266539	2992	1808	29	SRR1661223_megahit.fa	2992	1808	29		SRR1661223_spades.fa	2992	1808	29		SRR1661223_before_rr.fa	2992	1808	29
SRR1661224	PRJNA266539	210	59	4	SRR1661224_megahit.fa	210	59	4		SRR1661224_spades.fa	210	59	4		SRR1661224_before_rr.fa	210	59	4
SRR1661225	PRJNA266539	210	59	4	SRR1661225_megahit.fa	210	59	4		SRR1661225_spades.fa	210	59	4		SRR1661225_before_rr.fa	210	59	4
SRR1661226	PRJNA266539	3150	1907	4	SRR1661226_megahit.fa	3150	1907	4		SRR1661226_spades.fa	3150	1907	4		SRR1661226_before_rr.fa	3150	1907	4
SRR1661227	PRJNA266539	4980	3019	4	SRR1661227_megahit.fa	4980	3019	4		SRR1661227_spades.fa	4980	3019	4		SRR1661227_before_rr.fa	4980	3019	4
SRR1661228	PRJNA266539	757	262	4	SRR1661228_megahit.fa	757	262	4		SRR1661228_spades.fa	757	262	4		SRR1661228_before_rr.fa	757	262	4
SRR1661229	PRJNA266539	2992	1808	29	SRR1661229_megahit.fa	2992	1808	29		SRR1661229_spades.fa	2992	1808	29		SRR1661229_before_rr.fa	2992	1808	29
SRR1661230	PRJNA266539	4269	2623	110	SRR1661230_megahit.fa	4269	2623	110		SRR1661230_spades.fa	4269	2623	110		SRR1661230_before_rr.fa	4269	2623	110
SRR1661231	PRJNA266539	4269	2623	110	SRR1661231_megahit.fa	4269	2623	110		SRR1661231_spades.fa	4269	2623	110		SRR1661231_before_rr.fa	4269	2623	110
SRR1661232	PRJNA266539	4637	1910	27	SRR1661232_megahit.fa	4637	1910	27		SRR1661232_spades.fa	4637	1910	27		SRR1661232_before_rr.fa	4637	1910	27
SRR1661233	PRJNA266539	1407	908	110	SRR1661233_megahit.fa	-	908	-	missing	SRR1661233_spades.fa	1407	908	110		SRR1661233_before_rr.fa	1407	908	110
SRR1661234	PRJNA266539	5371	3256	16	SRR1661234_megahit.fa	5371	3256	16		SRR1661234_spades.fa	5371	3256	16		SRR1661234_before_rr.fa	5371	3256	16
SRR1661235	PRJNA266539	5371	3256	16	SRR1661235_megahit.fa	5371	3256	16		SRR1661235_spades.fa	5371	3256	16		SRR1661235_before_rr.fa	5371	3256	16
SRR1661236	PRJNA266539	51	39	27	SRR1661236_megahit.fa	51	39	27		SRR1661236_spades.fa	51	39	27		SRR1661236_before_rr.fa	51	39	27
SRR1661237	PRJNA266539	6351	3299	16	SRR1661237_megahit.fa	6351	3299	16		SRR1661237_spades.fa	6351	3299	16		SRR1661237_before_rr.fa	6351	3299	16
SRR1661238	PRJNA266539	231	343	16	SRR1661238_megahit.fa	231	343	16		SRR1661238_spades.fa	231	343	16		SRR1661238_before_rr.fa	231	343	16
SRR1661239	PRJNA266539	6644	3664	16	SRR1661239_megahit.fa	6644	3664	16		SRR1661239_spades.fa	6644	3664	16		SRR1661239_before_rr.fa	6644	3664	16
SRR1661240	PRJNA266539	6645	3675	16	SRR1661240_megahit.fa	6645	3675	16		SRR1661240_spades.fa	6645	3675	16		SRR1661240_before_rr.fa	6645	3675	16

SRR1661241	PRJNA266539	231	343	16	SRR1661241_megahit.fa	231	343	16		SRR1661241_spades.fa	231	343	16		SRR1661241_before_rr.fa	231	343	16
SRR1661242	PRJNA266539	1407	908	110	SRR1661242_megahit.fa	1407	908	110		SRR1661242_spades.fa	1407	908	110		SRR1661242_before_rr.fa	1407	908	110
SRR1661243	PRJNA266539	3150	1907	4	SRR1661243_megahit.fa	3150	1907	4		SRR1661243_spades.fa	3150	1907	4		SRR1661243_before_rr.fa	3150	1907	4
SRR1661244	PRJNA266539	4985	3024	110	SRR1661244_megahit.fa	4985	3024	110		SRR1661244_spades.fa	4985	3024	110		SRR1661244_before_rr.fa	4985	3024	110
SRR1661245	PRJNA266539	5372	3257	110	SRR1661245_megahit.fa	5372	3257	110		SRR1661245_spades.fa	-	new	110	spades assembly error	SRR1661245_before_rr.fa	5372	3257	110
SRR1661246	PRJNA266539	1407	908	110	SRR1661246_megahit.fa	1407	908	110		SRR1661246_spades.fa	1407	908	110		SRR1661246_before_rr.fa	1407	908	110
SRR1661247	PRJNA266539	1407	908	110	SRR1661247_megahit.fa	1407	908	110		SRR1661247_spades.fa	1407	908	110		SRR1661247_before_rr.fa	1407	908	110
SRR1661248	PRJNA266539	4985	3024	110	SRR1661248_megahit.fa	4985	3024	110		SRR1661248_spades.fa	4985	3024	110		SRR1661248_before_rr.fa	4985	3024	110
SRR1661249	PRJNA266539	1407	908	110	SRR1661249_megahit.fa	1407	908	110		SRR1661249_spades.fa	-	908	new	spades assembly error	SRR1661249_before_rr.fa	1407	908	110
SRR1661250	PRJNA266539	3378	2043	110	SRR1661250_megahit.fa	3378	2043	110		SRR1661250_spades.fa	3378	2043	110		SRR1661250_before_rr.fa	3378	2043	110
SRR1661251	PRJNA266539	4985	3024	110	SRR1661251_megahit.fa	4985	3024	110		SRR1661251_spades.fa	4985	3024	110		SRR1661251_before_rr.fa	4985	3024	110
SRR1661252	PRJNA266539	3550	4	49	SRR1661252_megahit.fa	3550	4	49		SRR1661252_spades.fa	3550	4	49		SRR1661252_before_rr.fa	3550	4	49
SRR1661253	PRJNA266539	3149	1903	110	SRR1661253_megahit.fa	3149	1903	110		SRR1661253_spades.fa	3149	1903	110		SRR1661253_before_rr.fa	3149	1903	110
SRR1661254	PRJNA266539	5643	3424	110	SRR1661254_megahit.fa	5643	3424	110		SRR1661254_spades.fa	5643	3424	110		SRR1661254_before_rr.fa	5643	3424	110
SRR1661255	PRJNA266539	3556	2155	16	SRR1661255_megahit.fa	3556	2155	16		SRR1661255_spades.fa	3556	2155	16		SRR1661255_before_rr.fa	3556	2155	16
SRR1661256	PRJNA266539	3556	2155	16	SRR1661256_megahit.fa	3556	2155	16		SRR1661256_spades.fa	3556	2155	16		SRR1661256_before_rr.fa	3556	2155	16
SRR1661257	PRJNA266539	25	18	27	SRR1661257_megahit.fa	25	18	27		SRR1661257_spades.fa	25	18	27		SRR1661257_before_rr.fa	25	18	27
SRR1661258	PRJNA266539	3150	1907	4	SRR1661258_megahit.fa	3150	1907	4		SRR1661258_spades.fa	3150	1907	4		SRR1661258_before_rr.fa	3150	1907	4
SRR1661259	PRJNA266539	1407	908	110	SRR1661259_megahit.fa	1407	908	110		SRR1661259_spades.fa	1407	908	110		SRR1661259_before_rr.fa	1407	908	110
SRR1661260	PRJNA266539	6175	3662	16	SRR1661260_megahit.fa	6175	3662	16		SRR1661260_spades.fa	6175	3662	16		SRR1661260_before_rr.fa	6175	3662	16
SRR1661261	PRJNA266539	2992	1808	29	SRR1661261_megahit.fa	2992	1808	29		SRR1661261_spades.fa	2992	1808	29		SRR1661261_before_rr.fa	2992	1808	29
SRR1661262	PRJNA266539	1407	908	110	SRR1661262_megahit.fa	1407	908	110		SRR1661262_spades.fa	1407	908	110		SRR1661262_before_rr.fa	1407	908	110
SRR1661263	PRJNA266539	4669	96	986	SRR1661263_megahit.fa	4669	96	986		SRR1661263_spades.fa	4669	96	986	alternate key, but detected	SRR1661263_before_rr.fa	4669	96	986
SRR1661264	PRJNA266539	545	63	171	SRR1661264_megahit.fa	545	63	171		SRR1661264_spades.fa	545	63	171		SRR1661264_before_rr.fa	545	63	171
SRR1661265	PRJNA266539	757	262	4	SRR1661265_megahit.fa	757	262	4		SRR1661265_spades.fa	757	262	4		SRR1661265_before_rr.fa	757	262	4
SRR1661266	PRJNA266539	2	2	16	SRR1661266_megahit.fa	2	2	16		SRR1661266_spades.fa	2	2	16		SRR1661266_before_rr.fa	2	2	16
SRR1661267	PRJNA266539	2	2	16	SRR1661267_megahit.fa	2	2	16		SRR1661267_spades.fa	2	2	16		SRR1661267_before_rr.fa	2	2	16
SRR1661268	PRJNA266539	5643	3424	110	SRR1661268_megahit.fa	5643	3424	110		SRR1661268_spades.fa	5643	3424	110		SRR1661268_before_rr.fa	5643	3424	110
SRR1661269	PRJNA266539	5643	3424	110	SRR1661269_megahit.fa	5643	3424	110		SRR1661269_spades.fa	-	3424	new	spades assembly error	SRR1661269_before_rr.fa	5643	3424	110
SRR1661270	PRJNA266539	3709	2237	110	SRR1661270_megahit.fa	3709	2237	110		SRR1661270_spades.fa	3709	2237	110		SRR1661270_before_rr.fa	3709	2237	110
SRR1661271	PRJNA266539	3158	1914	110	SRR1661271_megahit.fa	3158	1914	110		SRR1661271_spades.fa	3158	1914	110		SRR1661271_before_rr.fa	3158	1914	110
SRR1661272	PRJNA266539	5643	3424	110	SRR1661272_megahit.fa	5643	3424	110		SRR1661272_spades.fa	5643	3424	110		SRR1661272_before_rr.fa	5643	3424	110
SRR1661273	PRJNA266539	1513	971	110	SRR1661273_megahit.fa	1513	971	110		SRR1661273_spades.fa	1513	971	110		SRR1661273_before_rr.fa	1513	971	110
SRR1661274	PRJNA266539	1407	908	110	SRR1661274_megahit.fa	1407	908	110		SRR1661274_spades.fa	1407	908	110		SRR1661274_before_rr.fa	1407	908	110
SRR1661275	PRJNA266539	7159	1245	18	SRR1661275_megahit.fa	7159	1245	18		SRR1661275_spades.fa	7159	1245	18		SRR1661275_before_rr.fa	7159	1245	18
SRR1661276	PRJNA266539	1407	908	110	SRR1661276_megahit.fa	1407	908	110		SRR1661276_spades.fa	-	908	new	spades assembly error	SRR1661276_before_rr.fa	1407	908	110
SRR1661277	PRJNA266539	1407	908	110	SRR1661277_megahit.fa	1407	908	110		SRR1661277_spades.fa	1407	908	110		SRR1661277_before_rr.fa	1407	908	110
SRR1661278	PRJNA266539	1407	908	110	SRR1661278_megahit.fa	1407	908	110		SRR1661278_spades.fa	1407	908	110		SRR1661278_before_rr.fa	1407	908	110
SRR1661279	PRJNA266539	1407	908	110	SRR1661279_megahit.fa	1407	908	110		SRR1661279_spades.fa	1407	908	110		SRR1661279_before_rr.fa	1407	908	110
SRR1661280	PRJNA266539	225	4	4	SRR1661280_megahit.fa	225	4	4		SRR1661280_spades.fa	225	4	4		SRR1661280_before_rr.fa	225	4	4
SRR1661281	PRJNA266539	2992	1808	29	SRR1661281_megahit.fa	2992	1808	29		SRR1661281_spades.fa	2992	1808	29		SRR1661281_before_rr.fa	2992	1808	29
SRR1661282	PRJNA266539	1780	1143	39	SRR1661282_megahit.fa	1780	1143	39		SRR1661282_spades.fa	1780	1143	39		SRR1661282_before_rr.fa	1780	1143	39
SRR1661283	PRJNA266539	1407	908	110	SRR1661283_megahit.fa	1407	908	110		SRR1661283_spades.fa	-	908	new	spades assembly error	SRR1661283_before_rr.fa	1407	908	110
SRR1661284	PRJNA266539	1407	908	110	SRR1661284_megahit.fa	1407	908	110		SRR1661284_spades.fa	-	908	new	spades assembly error	SRR1661284_before_rr.fa	1407	908	110
SRR1661285	PRJNA266539	3556	2155	16	SRR1661285_megahit.fa	3556	2155	16		SRR1661285_spades.fa	3556	2155	16		SRR1661285_before_rr.fa	3556	2155	16
SRR1661286	PRJNA266539	231	343	16	SRR1661286_megahit.fa	231	343	16		SRR1661286_spades.fa	231	343	16		SRR1661286_before_rr.fa	231	343	16
SRR1661287	PRJNA266539	7983	4759	16	SRR1661287_megahit.fa	7983	4759	16		SRR1661287_spades.fa	7983	4759	16		SRR1661287_before_rr.fa	7983	4759	16
SRR1661288	PRJNA266539	6644	3664	16	SRR1661288_megahit.fa	6644	3664	16		SRR1661288_spades.fa	6644	3664	16		SRR1661288_before_rr.fa	6644	3664	16
SRR1661289	PRJNA266539	8	7	3	SRR1661289_megahit.fa	8	7	3		SRR1661289_spades.fa	8	7	3		SRR1661289_before_rr.fa	8	7	3
SRR1661290	PRJNA266539	374	316	16	SRR1661290_megahit.fa	374	316	16		SRR1661290_spades.fa	374	316	16		SRR1661290_before_rr.fa	374	316	16
SRR1661291	PRJNA266539	374	316	16	SRR1661291_megahit.fa	374	316	16		SRR1661291_spades.fa	374	316	16		SRR1661291_before_rr.fa	374	316	16
SRR1661292	PRJNA266539	1407	908	110	SRR1661292_megahit.fa	1407	908	110		SRR1661292_spades.fa	1407	908	110		SRR1661292_before_rr.fa	1407	908	110
SRR1661293	PRJNA266539	7986	4700	4	SRR1661293_megahit.fa	7986	4700	4		SRR1661293_spades.fa	7986	4700	4		SRR1661293_before_rr.fa	7986	4700	4
SRR1661294	PRJNA266539	1407	908	110	SRR1661294_megahit.fa	1407	908	110		SRR1661294_spades.fa	1407	908	110		SRR1661294_before_rr.fa	1407	908	110
SRR1661295	PRJNA266539	5650	3428	4	SRR1661295_megahit.fa	5650	3428	4		SRR1661295_spades.fa	5650	3428	4		SRR1661295_before_rr.fa	5650	3428	4
SRR1661296	PRJNA266539	8851	5230	156	SRR1661296_megahit.fa	8851	5230	156		SRR1661296_spades.fa	8851	5230	156		SRR1661296_before_rr.fa	8851	5230	156
SRR1661297	PRJNA266539	7986	4700	4	SRR1661297_megahit.fa	7986	4700	4		SRR1661297_spades.fa	7986	4700	4		SRR1661297_before_rr.fa	7986	4700	4
SRR1661298	PRJNA266539	3378	2043	110	SRR1661298_megahit.fa	3378	2043	110		SRR1661298_spades.fa	3378	2043	110		SRR1661298_before_rr.fa	3378	2043	110
SRR1661299	PRJNA266539	1407	908	110	SRR1661299_megahit.fa	1407	908	110		SRR1661299_spades.fa	1407	908	110		SRR1661299_before_rr.fa	1407	908	110
SRR1661300	PRJNA266539	7986	4700	4	SRR1661300_megahit.fa	7986	4700	4		SRR1661300_spades.fa	7986	4700	4		SRR1661300_before_rr.fa	7986	4700	4
SRR1661301	PRJNA266539	1407	908	110	SRR1661301_megahit.fa	1407	908	110		SRR1661301_spades.fa	1407	908	110		SRR1661301_before_rr.fa	1407	908	110
SRR1661302	PRJNA266539	3158	1914	110	SRR1661302_megahit.fa	3158	1914	110		SRR1661302_spades.fa	3158	1914	110		SRR1661302_before_rr.fa	3158	1914	110
SRR1661303	PRJNA266539	7986	4700	4	SRR1661303_megahit.fa	7986	4700	4		SRR1661303_spades.fa	7986	4700	4		SRR1661303_before_rr.fa	7986	4700	4
SRR1661304	PRJNA266539	1407	908	110	SRR1661304_megahit.fa	1407	908	110		SRR1661304_spades.fa	-	908	new	spades assembly error	SRR1661304_before_rr.fa	1407	908	110
SRR1661305	PRJNA266539	7986	4700	4	SRR1661305_megahit.fa	7986	4700	4		SRR1661305_spades.fa	7986	4700	4		SRR1661305_before_rr.fa	7986	4700	4
SRR1661306	PRJNA266539	7986	4700	4	SRR1661306_megahit.fa	7986	4700	4		SRR1661306_spades.fa	7986	4700	4		SRR1661306_before_rr.fa	7986	4700	4
SRR1661307	PRJNA266539	5640	1914	1179	SRR1661307_megahit.fa	5640	1914	1179		SRR1661307_spades.fa	5640	1914	1179		SRR1661307_before_rr.fa	5640	1914	1179
SRR1661308	PRJNA266539	4985	3024	110	SRR1661308_megahit.fa	4985	3024	110		SRR1661308_spades.fa	4985	3024	110		SRR1661308_before_rr.fa	4985	3024	110
SRR1661309	PRJNA266539	225	4	4	SRR1661309_megahit.fa	225	4	4		SRR1661309_spades.fa	225	4	4		SRR1661309_before_rr.fa	225	4	4
SRR1661310	PRJNA266539	3158	1914	110	SRR1661310_megahit.fa	-	-	110	missing	SRR1661310_spades.fa	3158	1914	110		SRR1661310_before_rr.fa	3158	1914	110
SRR1661311	PRJNA266539	3158	1914	110	SRR1661311_megahit.fa	3158	1914	110		SRR1661311_spades.fa	-	1914	new	spades assembly error	SRR1661311_before_rr.fa	3158	1914	110
SRR1661312	PRJNA266539	495	91	32	SRR1661312_megahit.fa	-	-	32	missing	SRR1661312_spades.fa	495	91	32		SRR1661312_before_rr.fa	495	91	32
SRR1661313	PRJNA266539	2992	1808	29	SRR1661313_megahit.fa	2992	1808	29		SRR1661313_spades.fa	2992	1808	29		SRR1661313_before_rr.fa	2992	1808	29
SRR1661314	PRJNA266539	210	59	4	SRR1661314_megahit.fa	210	59	4		SRR1661314_spades.fa	210	59	4		SRR1661314_before_rr.fa	210	59	4
SRR1661315	PRJNA266539	3158	1914	110	SRR1661315_megahit.fa	3158	1914	110		SRR1661315_spades.fa	3158	1914	110		SRR1661315_before_rr.fa	3158	1914	110
SRR1661316	PRJNA266539	8694	5150	29	SRR1661316_megahit.fa	8694	5150	29		SRR1661316_spades.fa	8694	5150	29		SRR1661316_before_rr.fa	8694	5150	29
SRR1661317	PRJNA266539	3656	356	821	SRR1661317_megahit.fa	3656	356	821		SRR1661317_spades.fa	3656	356	821		SRR1661317_before_rr.fa	3656	356	821
SRR1661318	PRJNA266539	3657	2193	27	SRR1661318_megahit.fa	3657	2193	27		SRR1661318_spades.fa	3657	2193	27		SRR1661318_before_rr.fa	3657	2193	27
SRR1661319	PRJNA266539	6331	3733	3	SRR1661319_megahit.fa	6331	3733	3		SRR1661319_spades.fa	6331	3733	3		SRR1661319_before_rr.fa	6331	3733	3
SRR1661320	PRJNA266539	1109	18	25	SRR1661320_megahit.fa	1109	18	25		SRR1661320_spades.fa	1109	18	25		SRR1661320_before_rr.fa	1109	18	25
SRR1661321	PRJNA266539	9509	5644	16	SRR1661321_megahit.fa	9509	5644	16		SRR1661321_spades.fa	9509	5644	16		SRR1661321_before_rr.fa	9509	5644	16
SRR1661322	PRJNA266539	919	206	39	SRR1661322_megahit.fa	919	206	39		SRR1661322_spades.fa	919	206	39		SRR1661322_before_rr.fa	919	206	39
SRR1661323	PRJNA266539	1572	1022	6	SRR1661323_megahit.fa	1572	1022	6		SRR1661323_spades.fa	1572	1022	6		SRR1661323_before_rr.fa	1572	1022	6
SRR1661324	PRJNA266539	3303	2002	165	SRR1661324_megahit.fa	3303	2002	165		SRR1661324_spades.fa	3303	2002	165		SRR1661324_before_rr.fa	3303	2002	165
SRR1661325	PRJNA266539	621	90	18	SRR1661325_megahit.fa	621	90	18		SRR1661325_spades.fa	621	90	18		SRR1661325_before_rr.fa	621	90	18
SRR1661326	PRJNA266539	1424	917	10	SRR1661326_megahit.fa	1424	917	10		SRR1661326_spades.fa	1424	917	10		SRR1661326_before_rr.fa	1424	917	10
SRR1661327	PRJNA266539	1422	915	21	SRR1661327_megahit.fa	1422	915	21		SRR1661327_spades.fa	1422	915	21		SRR1661327_before_rr.fa	1422	915	21
SRR1661328	PRJNA266539	3304	2000	4	SRR1661328_megahit.fa	3304	2000	4		SRR1661328_spades.fa	3304	2000	4		SRR1661328_before_rr.fa	3304	2000	4
SRR1661329	PRJNA266539	556	90	122	SRR1661329_megahit.fa	556	90	122		SRR1661329_spades.fa	556	90	122		SRR1661329_before_rr.fa	556	90	122
SRR1661330	PRJNA266539	495	91	32	SRR1661330_megahit.fa	495	91	32		SRR1661330_spades.fa	495	91	32		SRR1661330_before_rr.fa	495	91	32
SRR1661331	PRJNA266539	3305	2001	29	SRR1661331_megahit.fa	3305	2001	29		SRR1661331_spades.fa	3305	2001	29		SRR1661331_before_rr.fa	3305	2001	29
SRR2736093	PRJNA298332	5	4	33	SRR2736093_megahit.fa	5	4	33		SRR2736093_spades.fa	5	4	33		SRR2736093_before_rr.fa	5	4	33

SRR2736094	PRJNA298332	21	14	33	SRR2736094_megahit.fa	21	14	33		SRR2736094_spades.fa	21	14	33		SRR2736094_before_rr.fa	21	14	33
SRR2736095	PRJNA298332	10502	241	33	SRR2736095_megahit.fa	10502	241	33		SRR2736095_spades.fa	10502	241	33		SRR2736095_before_rr.fa	10502	241	33
SRR2736096	PRJNA298332	10575	6178	110	SRR2736096_megahit.fa	10575	6178	110		SRR2736096_spades.fa	10575	6178	110		SRR2736096_before_rr.fa	10575	6178	110
SRR2736097	PRJNA298332	10576	6179	33	SRR2736097_megahit.fa	10576	6179	33		SRR2736097_spades.fa	10576	6179	33		SRR2736097_before_rr.fa	10576	6179	33
SRR2736098	PRJNA298332	21	14	33	SRR2736098_megahit.fa	21	14	33		SRR2736098_spades.fa	21	14	33		SRR2736098_before_rr.fa	21	14	33
SRR2736099	PRJNA298332	21	14	33	SRR2736099_megahit.fa	21	14	33		SRR2736099_spades.fa	21	14	33		SRR2736099_before_rr.fa	21	14	33
SRR2736100	PRJNA298332	21	14	33	SRR2736100_megahit.fa	21	14	33		SRR2736100_spades.fa	21	14	33		SRR2736100_before_rr.fa	21	14	33
SRR2736101	PRJNA298332	21	14	33	SRR2736101_megahit.fa	21	14	33		SRR2736101_spades.fa	21	14	33		SRR2736101_before_rr.fa	21	14	33
SRR2736102	PRJNA298332	21	14	33	SRR2736102_megahit.fa	21	14	33		SRR2736102_spades.fa	21	14	33		SRR2736102_before_rr.fa	21	14	33
SRR2736103	PRJNA298332	21	14	33	SRR2736103_megahit.fa	21	14	33		SRR2736103_spades.fa	21	14	33		SRR2736103_before_rr.fa	21	14	33
SRR2736104	PRJNA298332	5	4	33	SRR2736104_megahit.fa	5	4	33		SRR2736104_spades.fa	5	4	33		SRR2736104_before_rr.fa	5	4	33
SRR2736105	PRJNA298332	10577	6180	156	SRR2736105_megahit.fa	10577	6180	156		SRR2736105_spades.fa	10577	6180	156		SRR2736105_before_rr.fa	10577	6180	156
SRR2736106	PRJNA298332	5	4	33	SRR2736106_megahit.fa	5	4	33		SRR2736106_spades.fa	5	4	33		SRR2736106_before_rr.fa	5	4	33
SRR2736107	PRJNA298332	5	4	33	SRR2736107_megahit.fa	5	4	33		SRR2736107_spades.fa	5	4	33		SRR2736107_before_rr.fa	5	4	33
SRR2736108	PRJNA298332	5	4	33	SRR2736108_megahit.fa	5	4	33		SRR2736108_spades.fa	5	4	33		SRR2736108_before_rr.fa	5	4	33
SRR2736109	PRJNA298332	5	4	33	SRR2736109_megahit.fa	5	4	33		SRR2736109_spades.fa	5	4	33		SRR2736109_before_rr.fa	5	4	33
SRR2736110	PRJNA298332	64	50	29	SRR2736110_megahit.fa	64	50	29		SRR2736110_spades.fa	64	50	29		SRR2736110_before_rr.fa	64	50	29
SRR2736111	PRJNA298332	64	50	29	SRR2736111_megahit.fa	64	50	29		SRR2736111_spades.fa	64	50	29		SRR2736111_before_rr.fa	64	50	29
SRR2736112	PRJNA298332	5	4	33	SRR2736112_megahit.fa	5	4	33		SRR2736112_spades.fa	5	4	33		SRR2736112_before_rr.fa	5	4	33
SRR2736113	PRJNA298332	5	4	33	SRR2736113_megahit.fa	5	4	33		SRR2736113_spades.fa	5	4	33		SRR2736113_before_rr.fa	5	4	33
SRR2736114	PRJNA298332	10575	6178	110	SRR2736114_megahit.fa	10575	6178	110		SRR2736114_spades.fa	10575	6178	110		SRR2736114_before_rr.fa	10575	6178	110
SRR2736115	PRJNA298332	5	4	33	SRR2736115_megahit.fa	5	4	33		SRR2736115_spades.fa	5	4	33		SRR2736115_before_rr.fa	5	4	33
SRR2736116	PRJNA298332	10503	603	33	SRR2736116_megahit.fa	10503	603	33		SRR2736116_spades.fa	10503	603	33		SRR2736116_before_rr.fa	10503	603	33
SRR2736117	PRJNA298332	21	14	33	SRR2736117_megahit.fa	21	14	33		SRR2736117_spades.fa	21	14	33		SRR2736117_before_rr.fa	21	14	33
SRR2736118	PRJNA298332	10578	6181	9	SRR2736118_megahit.fa	10578	6181	9		SRR2736118_spades.fa	10578	6181	9		SRR2736118_before_rr.fa	10578	6181	9
SRR2736119	PRJNA298332	251	187	33	SRR2736119_megahit.fa	251	187	33		SRR2736119_spades.fa	251	187	33		SRR2736119_before_rr.fa	251	187	33
SRR2736120	PRJNA298332	21	14	33	SRR2736120_megahit.fa	21	14	33		SRR2736120_spades.fa	21	14	33		SRR2736120_before_rr.fa	21	14	33
SRR2736121	PRJNA298332	21	14	33	SRR2736121_megahit.fa	21	14	33		SRR2736121_spades.fa	21	14	33		SRR2736121_before_rr.fa	21	14	33
SRR2736122	PRJNA298332	21	14	33	SRR2736122_megahit.fa	21	14	33		SRR2736122_spades.fa	21	14	33		SRR2736122_before_rr.fa	21	14	33
SRR2736123	PRJNA298332	5	4	33	SRR2736123_megahit.fa	5	4	33		SRR2736123_spades.fa	5	4	33		SRR2736123_before_rr.fa	5	4	33
SRR2736124	PRJNA298332	21	14	33	SRR2736124_megahit.fa	21	14	33		SRR2736124_spades.fa	21	14	33		SRR2736124_before_rr.fa	21	14	33
SRR2736125	PRJNA298332	5	4	33	SRR2736125_megahit.fa	5	4	33		SRR2736125_spades.fa	5	4	33		SRR2736125_before_rr.fa	5	4	33
SRR2736126	PRJNA298332	21	14	33	SRR2736126_megahit.fa	21	14	33		SRR2736126_spades.fa	21	14	33		SRR2736126_before_rr.fa	21	14	33
SRR2736127	PRJNA298332	154	87	33	SRR2736127_megahit.fa	-	-	33	missing	SRR2736127_spades.fa	154	87	33		SRR2736127_before_rr.fa	154	87	33
SRR2736128	PRJNA298332	5	4	33	SRR2736128_megahit.fa	5	4	33		SRR2736128_spades.fa	5	4	33		SRR2736128_before_rr.fa	5	4	33
SRR2736129	PRJNA298332	5	4	33	SRR2736129_megahit.fa	5	4	33		SRR2736129_spades.fa	5	4	33		SRR2736129_before_rr.fa	5	4	33
SRR2736130	PRJNA298332	1116	334	33	SRR2736130_megahit.fa	1116	334	33		SRR2736130_spades.fa	1116	334	33		SRR2736130_before_rr.fa	1116	334	33
SRR2736131	PRJNA298332	64	50	29	SRR2736131_megahit.fa	-	-	29	missing	SRR2736131_spades.fa	64	50	29		SRR2736131_before_rr.fa	64	50	29
SRR2736132	PRJNA298332	5445	1582	33	SRR2736132_megahit.fa	5445	1582	33		SRR2736132_spades.fa	5445	1582	33		SRR2736132_before_rr.fa	5445	1582	33
SRR2736133	PRJNA298332	64	50	29	SRR2736133_megahit.fa	64	50	29		SRR2736133_spades.fa	64	50	29		SRR2736133_before_rr.fa	64	50	29
SRR2736134	PRJNA298332	1948	543	479	SRR2736134_megahit.fa	1948	543	479		SRR2736134_spades.fa	1948	543	479		SRR2736134_before_rr.fa	1948	543	479
SRR2736135	PRJNA298332	3132	719	4	SRR2736135_megahit.fa	3132	719	4		SRR2736135_spades.fa	3132	719	4		SRR2736135_before_rr.fa	3132	719	4
SRR2736136	PRJNA298332	64	50	29	SRR2736136_megahit.fa	64	50	29		SRR2736136_spades.fa	64	50	29		SRR2736136_before_rr.fa	64	50	29
SRR2736137	PRJNA298332	64	50	29	SRR2736137_megahit.fa	-	new	29	new	SRR2736137_spades.fa	-	new	29	de novo assembly across por; 1 SNP difference to por-50; nc	SRR2736137_before_rr.fa	-	new	29
SRR2736138	PRJNA298332	3155	1913	4	SRR2736138_megahit.fa	3155	1913	4		SRR2736138_spades.fa	3155	1913	4		SRR2736138_before_rr.fa	3155	1913	4
SRR2736139	PRJNA298332	3155	1913	4	SRR2736139_megahit.fa	3155	1913	4		SRR2736139_spades.fa	3155	1913	4		SRR2736139_before_rr.fa	3155	1913	4
SRR2736140	PRJNA298332	64	50	29	SRR2736140_megahit.fa	64	50	29		SRR2736140_spades.fa	64	50	29		SRR2736140_before_rr.fa	64	50	29
SRR2736141	PRJNA298332	225	4	4	SRR2736141_megahit.fa	225	4	4		SRR2736141_spades.fa	225	4	4		SRR2736141_before_rr.fa	225	4	4
SRR2736142	PRJNA298332	4813	2906	6	SRR2736142_megahit.fa	4813	2906	6		SRR2736142_spades.fa	4813	2906	6		SRR2736142_before_rr.fa	4813	2906	6
SRR2736143	PRJNA298332	3150	1907	4	SRR2736143_megahit.fa	3150	1907	4		SRR2736143_spades.fa	3150	1907	4		SRR2736143_before_rr.fa	3150	1907	4
SRR2736144	PRJNA298332	4815	2907	29	SRR2736144_megahit.fa	4815	2907	29		SRR2736144_spades.fa	4815	2907	29		SRR2736144_before_rr.fa	4815	2907	29
SRR2736145	PRJNA298332	4815	2907	29	SRR2736145_megahit.fa	4815	2907	29		SRR2736145_spades.fa	4815	2907	29		SRR2736145_before_rr.fa	4815	2907	29
SRR2736146	PRJNA298332	3116	1884	25	SRR2736146_megahit.fa	3116	1884	25		SRR2736146_spades.fa	3116	1884	25		SRR2736146_before_rr.fa	3116	1884	25
SRR2736147	PRJNA298332	892	604	4	SRR2736147_megahit.fa	892	604	4		SRR2736147_spades.fa	892	604	4		SRR2736147_before_rr.fa	892	604	4
SRR2736148	PRJNA298332	225	4	4	SRR2736148_megahit.fa	225	4	4		SRR2736148_spades.fa	225	4	4		SRR2736148_before_rr.fa	225	4	4
SRR2736149	PRJNA298332	4814	485	10	SRR2736149_megahit.fa	4814	485	10		SRR2736149_spades.fa	4814	485	10		SRR2736149_before_rr.fa	4814	485	10
SRR2736150	PRJNA298332	3158	1914	110	SRR2736150_megahit.fa	3158	1914	110		SRR2736150_spades.fa	3158	1914	110		SRR2736150_before_rr.fa	3158	1914	110
SRR2736151	PRJNA298332	225	4	4	SRR2736151_megahit.fa	225	4	4		SRR2736151_spades.fa	225	4	4		SRR2736151_before_rr.fa	225	4	4
SRR2736152	PRJNA298332	4815	2907	29	SRR2736152_megahit.fa	4815	2907	29		SRR2736152_spades.fa	4815	2907	29		SRR2736152_before_rr.fa	4815	2907	29
SRR2736153	PRJNA298332	4815	2907	29	SRR2736153_megahit.fa	4815	2907	29		SRR2736153_spades.fa	4815	2907	29		SRR2736153_before_rr.fa	4815	2907	29
SRR2736154	PRJNA298332	4827	182	508	SRR2736154_megahit.fa	4827	182	508		SRR2736154_spades.fa	4827	182	508		SRR2736154_before_rr.fa	4827	182	508
SRR2736155	PRJNA298332	4816	2704	4	SRR2736155_megahit.fa	4816	2704	4		SRR2736155_spades.fa	4816	2704	4		SRR2736155_before_rr.fa	4816	2704	4
SRR2736156	PRJNA298332	421	206	156	SRR2736156_megahit.fa	421	206	156		SRR2736156_spades.fa	421	206	156		SRR2736156_before_rr.fa	421	206	156
SRR2736157	PRJNA298332	4836	1447	1020	SRR2736157_megahit.fa	4836	1447	1020		SRR2736157_spades.fa	4836	1447	1020		SRR2736157_before_rr.fa	4836	1447	1020
SRR2736158	PRJNA298332	3132	719	4	SRR2736158_megahit.fa	3132	719	4		SRR2736158_spades.fa	3132	719	4		SRR2736158_before_rr.fa	3132	719	4
SRR2736159	PRJNA298332	2992	1808	29	SRR2736159_megahit.fa	2992	1808	29		SRR2736159_spades.fa	2992	1808	29		SRR2736159_before_rr.fa	2992	1808	29
SRR2736160	PRJNA298332	1407	908	110	SRR2736160_megahit.fa	1407	908	110		SRR2736160_spades.fa	1407	908	110		SRR2736160_before_rr.fa	1407	908	110
SRR2736161	PRJNA298332	1407	908	110	SRR2736161_megahit.fa	1407	908	110		SRR2736161_spades.fa	1407	908	110		SRR2736161_before_rr.fa	1407	908	110
SRR2736162	PRJNA298332	1407	908	110	SRR2736162_megahit.fa	1407	908	110		SRR2736162_spades.fa	1407	908	110		SRR2736162_before_rr.fa	1407	908	110
SRR2736163	PRJNA298332	1407	908	110	SRR2736163_megahit.fa	1407	908	110		SRR2736163_spades.fa	1407	908	110		SRR2736163_before_rr.fa	1407	908	110
SRR2736164	PRJNA298332	225	4	4	SRR2736164_megahit.fa	225	4	4		SRR2736164_spades.fa	225	4	4		SRR2736164_before_rr.fa	225	4	4
SRR2736165	PRJNA298332	285	232	4	SRR2736165_megahit.fa	285	232	4		SRR2736165_spades.fa	285	232	4		SRR2736165_before_rr.fa	285	232	4
SRR2736166	PRJNA298332	4809	2888	367	SRR2736166_megahit.fa	4809	2888	367		SRR2736166_spades.fa	4809	2888	367		SRR2736166_before_rr.fa	4809	2888	367
SRR2736167	PRJNA298332	3150	1907	4	SRR2736167_megahit.fa	3150	1907	4		SRR2736167_spades.fa	3150	1907	4		SRR2736167_before_rr.fa	3150	1907	4
SRR2736168	PRJNA298332	225	4	4	SRR2736168_megahit.fa	225	4	4		SRR2736168_spades.fa	225	4	4		SRR2736168_before_rr.fa	225	4	4
SRR2736169	PRJNA298332	688	98	4	SRR2736169_megahit.fa	688	98	4		SRR2736169_spades.fa	688	98	4		SRR2736169_before_rr.fa	688	98	4
SRR2736170	PRJNA298332	688	98	4	SRR2736170_megahit.fa	688	98	4		SRR2736170_spades.fa	688	98	4		SRR2736170_before_rr.fa	688	98	4
SRR2736171	PRJNA298332	688	98	4	SRR2736171_megahit.fa	688	98	4		SRR2736171_spades.fa	688	98	4		SRR2736171_before_rr.fa	688	98	4
SRR2736172	PRJNA298332	688	98	4	SRR2736172_megahit.fa	688	98	4		SRR2736172_spades.fa	688	98	4		SRR2736172_before_rr.fa	688	98	4
SRR2736173	PRJNA298332	688	98	4	SRR2736173_megahit.fa	688	98	4		SRR2736173_spades.fa	688	98	4		SRR2736173_before_rr.fa	688	98	4
SRR2736174	PRJNA298332	688	98	4	SRR2736174_megahit.fa	688	98	4		SRR2736174_spades.fa	688	98	4		SRR2736174_before_rr.fa	688	98	4
SRR2736175	PRJNA298332	3150	1907	4	SRR2736175_megahit.fa	3150	1907	4		SRR2736175_spades.fa	3150	1907	4		SRR2736175_before_rr.fa	3150	1907	4
SRR2736176	PRJNA298332	1407	908	110	SRR2736176_megahit.fa	688	98	4	sequence mixup?	SRR2736176_spades.fa	688	98	4	sequence mixed up with SRR2736178?	SRR2736176_before_rr.fa	688	98	4
SRR2736177	PRJNA298332	3150	1907	4	SRR2736177_megahit.fa	3150	1907	4		SRR2736177_spades.fa	3150	1907	4		SRR2736177_before_rr.fa	3150	1907	4
SRR2736178	PRJNA298332	688	98	4	SRR2736178_megahit.fa	1407	908	110	sequence mixup?	SRR2736178_spades.fa	1407	908	110	sequence mixed up with SRR2736176?	SRR2736178_before_rr.fa	1407	908	110
SRR2736179	PRJNA298332	688	98	4	SRR2736179_megahit.fa	688	98	4		SRR2736179_spades.fa	688	98	4		SRR2736179_before_rr.fa	688	98	4
SRR2736180	PRJNA298332	688	98	4	SRR2736180_megahit.fa	688	98	4		SRR2736180_spades.fa	688	98	4		SRR2736180_before_rr.fa	688	98	4
SRR2736181	PRJNA298332	2992	1808	29	SRR2736181_megahit.fa	2992	1808	29		SRR2736181_spades.fa	2992	1808	29		SRR2736181_before_rr.fa	2992	1808	29
SRR2736182	PRJNA298332	688	98	4	SRR2736182_megahit.fa	688	98	4		SRR2736182_spades.fa	688	98	4		SRR2736182_before_rr.fa	688	98	4
SRR2736183	PRJNA298332	688	98	4	SRR2736183_megahit.fa	688	98	4		SRR2736183_spades.fa	688	98	4		SRR2736183_before_rr.fa	688	98	4
SRR2736184	PRJNA298332	688	98	4	SRR2736184_megahit.fa	688	98	4		SRR2736184_spades.fa	688	98	4		SRR2736184_before_rr.fa	688	98	4
SRR2736185	PRJNA298332	3150	1907	4	SRR2736185_megahit.fa	3150	1907	4		SRR2736185_spades.fa	3150	1907	4		SRR2736185_before_rr.fa	3150	1907	4

SRR2736186	PRJNA298332	3150	1907	4	SRR2736186_megahit.fa	3150	1907	4		SRR2736186_spades.fa	-	1907	new	spades assembly error	SRR2736186_before_rr.fa	3150	1907	4
SRR2736187	PRJNA298332	2992	1808	29	SRR2736187_megahit.fa	2992	1808	29		SRR2736187_spades.fa	2992	1808	29		SRR2736187_before_rr.fa	2992	1808	29
SRR2736188	PRJNA298332	2992	1808	29	SRR2736188_megahit.fa	2992	1808	29		SRR2736188_spades.fa	2992	1808	29		SRR2736188_before_rr.fa	2992	1808	29
SRR2736189	PRJNA298332	2992	1808	29	SRR2736189_megahit.fa	2992	1808	29		SRR2736189_spades.fa	2992	1808	29		SRR2736189_before_rr.fa	2992	1808	29
SRR2736190	PRJNA298332	2992	1808	29	SRR2736190_megahit.fa	2992	1808	29		SRR2736190_spades.fa	2992	1808	29		SRR2736190_before_rr.fa	2992	1808	29
SRR2736191	PRJNA298332	6296	3706	29	SRR2736191_megahit.fa	6296	3706	29		SRR2736191_spades.fa	6296	3706	29		SRR2736191_before_rr.fa	6296	3706	29
SRR2736192	PRJNA298332	2992	1808	29	SRR2736192_megahit.fa	2992	1808	29		SRR2736192_spades.fa	2992	1808	29		SRR2736192_before_rr.fa	2992	1808	29
SRR2736193	PRJNA298332	757	262	4	SRR2736193_megahit.fa	757	262	4		SRR2736193_spades.fa	757	262	4		SRR2736193_before_rr.fa	757	262	4
SRR2736194	PRJNA298332	2992	1808	29	SRR2736194_megahit.fa	2992	1808	29		SRR2736194_spades.fa	2992	1808	29		SRR2736194_before_rr.fa	2992	1808	29
SRR2736195	PRJNA298332	2992	1808	29	SRR2736195_megahit.fa	2992	1808	29		SRR2736195_spades.fa	2992	1808	29		SRR2736195_before_rr.fa	2992	1808	29
SRR2736196	PRJNA298332	2992	1808	29	SRR2736196_megahit.fa	2992	1808	29		SRR2736196_spades.fa	2992	1808	29		SRR2736196_before_rr.fa	2992	1808	29
SRR2736197	PRJNA298332	2992	1808	29	SRR2736197_megahit.fa	2992	1808	29		SRR2736197_spades.fa	2992	1808	29		SRR2736197_before_rr.fa	2992	1808	29
SRR2736198	PRJNA298332	3149	1903	110	SRR2736198_megahit.fa	437	14	4	sequence mixup?	SRR2736198_spades.fa	437	14	4	sequence mixed up with SRR2736209?	SRR2736198_before_rr.fa	437	14	4
SRR2736199	PRJNA298332	1407	908	110	SRR2736199_megahit.fa	1407	908	110		SRR2736199_spades.fa	1407	908	110		SRR2736199_before_rr.fa	1407	908	110
SRR2736200	PRJNA298332	688	98	4	SRR2736200_megahit.fa	688	98	4		SRR2736200_spades.fa	688	98	4		SRR2736200_before_rr.fa	688	98	4
SRR2736201	PRJNA298332	688	98	4	SRR2736201_megahit.fa	688	98	4		SRR2736201_spades.fa	688	98	4		SRR2736201_before_rr.fa	688	98	4
SRR2736202	PRJNA298332	5049	3059	29	SRR2736202_megahit.fa	5049	3059	29		SRR2736202_spades.fa	5049	3059	29		SRR2736202_before_rr.fa	5049	3059	29
SRR2736203	PRJNA298332	5343	581	1131	SRR2736203_megahit.fa	5343	581	1131		SRR2736203_spades.fa	5343	581	1131		SRR2736203_before_rr.fa	5343	581	1131
SRR2736204	PRJNA298332	757	262	4	SRR2736204_megahit.fa	757	262	4		SRR2736204_spades.fa	757	262	4		SRR2736204_before_rr.fa	757	262	4
SRR2736205	PRJNA298332	3150	1907	4	SRR2736205_megahit.fa	3150	1907	4		SRR2736205_spades.fa	3150	1907	4		SRR2736205_before_rr.fa	3150	1907	4
SRR2736206	PRJNA298332	7199	4309	29	SRR2736206_megahit.fa	7199	4309	29		SRR2736206_spades.fa	7199	4309	29		SRR2736206_before_rr.fa	7199	4309	29
SRR2736207	PRJNA298332	7199	4309	29	SRR2736207_megahit.fa	7199	4309	29		SRR2736207_spades.fa	7199	4309	29		SRR2736207_before_rr.fa	7199	4309	29
SRR2736208	PRJNA298332	8035	4790	29	SRR2736208_megahit.fa	8035	4790	29		SRR2736208_spades.fa	8035	4790	29		SRR2736208_before_rr.fa	8035	4790	29
SRR2736209	PRJNA298332	437	14	4	SRR2736209_megahit.fa	3149	1903	110	sequence mixup?	SRR2736209_spades.fa	3149	1903	110	sequence mixed up with SRR2736198?	SRR2736209_before_rr.fa	3149	1903	110
SRR2736210	PRJNA298332	2992	1808	29	SRR2736210_megahit.fa	2992	1808	29		SRR2736210_spades.fa	2992	1808	29		SRR2736210_before_rr.fa	2992	1808	29
SRR2736211	PRJNA298332	2992	1808	29	SRR2736211_megahit.fa	2992	1808	29		SRR2736211_spades.fa	2992	1808	29		SRR2736211_before_rr.fa	2992	1808	29
SRR2736212	PRJNA298332	3935	908	29	SRR2736212_megahit.fa	3935	908	29		SRR2736212_spades.fa	3935	908	29		SRR2736212_before_rr.fa	3935	908	29
SRR2736213	PRJNA298332	4991	3029	4	SRR2736213_megahit.fa	4991	3029	4		SRR2736213_spades.fa	4991	3029	4		SRR2736213_before_rr.fa	4991	3029	4
SRR2736214	PRJNA298332	8690	5146	4	SRR2736214_megahit.fa	8690	5146	4		SRR2736214_spades.fa	8690	5146	4		SRR2736214_before_rr.fa	8690	5146	4
SRR2736215	PRJNA298332	7986	4700	4	SRR2736215_megahit.fa	7986	4700	4		SRR2736215_spades.fa	7986	4700	4		SRR2736215_before_rr.fa	7986	4700	4
SRR2736216	PRJNA298332	7986	4700	4	SRR2736216_megahit.fa	7986	4700	4		SRR2736216_spades.fa	7986	4700	4		SRR2736216_before_rr.fa	7986	4700	4
SRR2736217	PRJNA298332	8203	2161	980	SRR2736217_megahit.fa	8203	2161	980		SRR2736217_spades.fa	8203	2161	980		SRR2736217_before_rr.fa	8203	2161	980
SRR2736218	PRJNA298332	5650	3428	4	SRR2736218_megahit.fa	multiple	3428	4/29	multiple	SRR2736218_spades.fa	5650	3428	4		SRR2736218_before_rr.fa	5650	3428	4
SRR2736219	PRJNA298332	2992	1808	29	SRR2736219_megahit.fa	2992	1808	29		SRR2736219_spades.fa	2992	1808	29		SRR2736219_before_rr.fa	2992	1808	29
SRR2736220	PRJNA298332	1407	908	110	SRR2736220_megahit.fa	1407	908	110		SRR2736220_spades.fa	1407	908	110		SRR2736220_before_rr.fa	1407	908	110
SRR2736221	PRJNA298332	7199	4309	29	SRR2736221_megahit.fa	7199	4309	29		SRR2736221_spades.fa	7199	4309	29		SRR2736221_before_rr.fa	7199	4309	29
SRR2736222	PRJNA298332	3158	1914	110	SRR2736222_megahit.fa	3158	1914	110		SRR2736222_spades.fa	3158	1914	110		SRR2736222_before_rr.fa	3158	1914	110
SRR2736223	PRJNA298332	3158	1914	110	SRR2736223_megahit.fa	3158	1914	110		SRR2736223_spades.fa	3158	1914	110		SRR2736223_before_rr.fa	3158	1914	110
SRR2736224	PRJNA298332	3158	1914	110	SRR2736224_megahit.fa	3158	1914	110		SRR2736224_spades.fa	3158	1914	110		SRR2736224_before_rr.fa	3158	1914	110
SRR2736225	PRJNA298332	8915	5268	29	SRR2736225_megahit.fa	-	-	29	missing	SRR2736225_spades.fa	8915	5268	29		SRR2736225_before_rr.fa	8915	5268	29
SRR2736226	PRJNA298332	3158	1914	110	SRR2736226_megahit.fa	3158	1914	110		SRR2736226_spades.fa	3158	1914	110		SRR2736226_before_rr.fa	3158	1914	110
SRR2736227	PRJNA298332	3158	1914	110	SRR2736227_megahit.fa	3158	1914	110		SRR2736227_spades.fa	3158	1914	110		SRR2736227_before_rr.fa	3158	1914	110
SRR2736228	PRJNA298332	3158	1914	110	SRR2736228_megahit.fa	-	-	110	missing	SRR2736228_spades.fa	3158	1914	110		SRR2736228_before_rr.fa	3158	1914	110
SRR2736229	PRJNA298332	3158	1914	110	SRR2736229_megahit.fa	3158	1914	110		SRR2736229_spades.fa	3158	1914	110		SRR2736229_before_rr.fa	3158	1914	110
SRR2736230	PRJNA298332	3158	1914	110	SRR2736230_megahit.fa	3158	1914	110		SRR2736230_spades.fa	3158	1914	110		SRR2736230_before_rr.fa	3158	1914	110
SRR2736231	PRJNA298332	9436	260	1710	SRR2736231_megahit.fa	9436	260	1710		SRR2736231_spades.fa	9436	260	1710		SRR2736231_before_rr.fa	9436	260	1710
SRR2736232	PRJNA298332	3158	1914	110	SRR2736232_megahit.fa	3158	1914	110		SRR2736232_spades.fa	3158	1914	110		SRR2736232_before_rr.fa	3158	1914	110
SRR2736233	PRJNA298332	2992	1808	29	SRR2736233_megahit.fa	2992	1808	29		SRR2736233_spades.fa	2992	1808	29		SRR2736233_before_rr.fa	2992	1808	29
SRR2736234	PRJNA298332	2992	1808	29	SRR2736234_megahit.fa	2992	1808	29		SRR2736234_spades.fa	2992	1808	29		SRR2736234_before_rr.fa	2992	1808	29
SRR2736235	PRJNA298332	2992	1808	29	SRR2736235_megahit.fa	2992	1808	29		SRR2736235_spades.fa	2992	1808	29		SRR2736235_before_rr.fa	2992	1808	29
SRR2736236	PRJNA298332	2992	1808	29	SRR2736236_megahit.fa	2992	1808	29		SRR2736236_spades.fa	2992	1808	29		SRR2736236_before_rr.fa	2992	1808	29
SRR2736237	PRJNA298332	2992	1808	29	SRR2736237_megahit.fa	2992	1808	29		SRR2736237_spades.fa	2992	1808	29		SRR2736237_before_rr.fa	2992	1808	29
SRR2736238	PRJNA298332	2992	1808	29	SRR2736238_megahit.fa	2992	1808	29		SRR2736238_spades.fa	2992	1808	29		SRR2736238_before_rr.fa	2992	1808	29
SRR2736239	PRJNA298332	2992	1808	29	SRR2736239_megahit.fa	2992	1808	29		SRR2736239_spades.fa	2992	1808	29		SRR2736239_before_rr.fa	2992	1808	29
SRR2736240	PRJNA298332	2992	1808	29	SRR2736240_megahit.fa	2992	1808	29		SRR2736240_spades.fa	2992	1808	29		SRR2736240_before_rr.fa	2992	1808	29
SRR2736241	PRJNA298332	9427	5596	110	SRR2736241_megahit.fa	9427	5596	110		SRR2736241_spades.fa	9427	5596	110		SRR2736241_before_rr.fa	9427	5596	110
SRR2736242	PRJNA298332	8915	5268	29	SRR2736242_megahit.fa	8915	5268	29		SRR2736242_spades.fa	8915	5268	29		SRR2736242_before_rr.fa	8915	5268	29
SRR2736243	PRJNA298332	6360	3957	563	SRR2736243_megahit.fa	6360	3957	563		SRR2736243_spades.fa	6360	3957	563		SRR2736243_before_rr.fa	6360	3957	563
SRR2736244	PRJNA298332	9427	5596	110	SRR2736244_megahit.fa	-	-	110	missing	SRR2736244_spades.fa	9427	5596	110		SRR2736244_before_rr.fa	9427	5596	110
SRR2736245	PRJNA298332	9427	5596	110	SRR2736245_megahit.fa	9427	5596	110		SRR2736245_spades.fa	9427	5596	110		SRR2736245_before_rr.fa	9427	5596	110
SRR2736246	PRJNA298332	9427	5596	110	SRR2736246_megahit.fa	9427	5596	110		SRR2736246_spades.fa	9427	5596	110		SRR2736246_before_rr.fa	9427	5596	110
SRR2736247	PRJNA298332	9427	5596	110	SRR2736247_megahit.fa	9427	5596	110		SRR2736247_spades.fa	9427	5596	110		SRR2736247_before_rr.fa	9427	5596	110
SRR2736248	PRJNA298332	9427	5596	110	SRR2736248_megahit.fa	9427	5596	110		SRR2736248_spades.fa	9427	5596	110		SRR2736248_before_rr.fa	9427	5596	110
SRR2736249	PRJNA298332	3935	908	29	SRR2736249_megahit.fa	3935	908	29		SRR2736249_spades.fa	3935	908	29		SRR2736249_before_rr.fa	3935	908	29
SRR2736250	PRJNA298332	9047	5351	29	SRR2736250_megahit.fa	-	-	-	missing	SRR2736250_spades.fa	multiple	5351	29/16	is dominant allele (assembled in context; allele 16 lone gene	SRR2736250_before_rr.fa	multiple	5351	29/16
SRR2736251	PRJNA298332	2992	1808	29	SRR2736251_megahit.fa	2992	1808	29		SRR2736251_spades.fa	2992	1808	29		SRR2736251_before_rr.fa	2992	1808	29
SRR2736252	PRJNA298332	2992	1808	29	SRR2736252_megahit.fa	2992	1808	29		SRR2736252_spades.fa	2992	1808	29		SRR2736252_before_rr.fa	2992	1808	29
SRR2736253	PRJNA298332	8015	4778	29	SRR2736253_megahit.fa	8015	4778	29		SRR2736253_spades.fa	8015	4778	29		SRR2736253_before_rr.fa	8015	4778	29
SRR2736254	PRJNA298332	3158	1914	110	SRR2736254_megahit.fa	3158	1914	110		SRR2736254_spades.fa	3158	1914	110		SRR2736254_before_rr.fa	3158	1914	110
SRR2736255	PRJNA298332	6765	2078	29	SRR2736255_megahit.fa	6765	2078	29		SRR2736255_spades.fa	6765	2078	29		SRR2736255_before_rr.fa	6765	2078	29
SRR2736256	PRJNA298332	6765	2078	29	SRR2736256_megahit.fa	6765	2078	29		SRR2736256_spades.fa	6765	2078	29		SRR2736256_before_rr.fa	6765	2078	29
SRR2736257	PRJNA298332	10128	5942	563	SRR2736257_megahit.fa	10128	5942	563		SRR2736257_spades.fa	10128	5942	563		SRR2736257_before_rr.fa	10128	5942	563
SRR2736258	PRJNA298332	3158	1914	110	SRR2736258_megahit.fa	3158	1914	110		SRR2736258_spades.fa	3158	1914	110		SRR2736258_before_rr.fa	3158	1914	110
SRR2736259	PRJNA298332	3158	1914	110	SRR2736259_megahit.fa	3158	1914	110		SRR2736259_spades.fa	3158	1914	110		SRR2736259_before_rr.fa	3158	1914	110
SRR2736260	PRJNA298332	1407	908	110	SRR2736260_megahit.fa	1407	908	110		SRR2736260_spades.fa	1407	908	110		SRR2736260_before_rr.fa	1407	908	110
SRR2736261	PRJNA298332	1034	430	33	SRR2736261_megahit.fa	1034	430	33		SRR2736261_spades.fa	1034	430	33		SRR2736261_before_rr.fa	1034	430	33
SRR2736262	PRJNA298332	9700	5722	33	SRR2736262_megahit.fa	9700	5722	33		SRR2736262_spades.fa	9700	5722	33		SRR2736262_before_rr.fa	9700	5722	33
SRR2736263	PRJNA298332	3158	1914	110	SRR2736263_megahit.fa	3158	1914	110		SRR2736263_spades.fa	3158	1914	110		SRR2736263_before_rr.fa	3158	1914	110
SRR2736264	PRJNA298332	3158	1914	110	SRR2736264_megahit.fa	3158	1914	110		SRR2736264_spades.fa	3158	1914	110		SRR2736264_before_rr.fa	3158	1914	110
SRR2736265	PRJNA298332	3158	1914	110	SRR2736265_megahit.fa	3158	1914	110		SRR2736265_spades.fa	3158	1914	110		SRR2736265_before_rr.fa	3158	1914	110
SRR2736266	PRJNA298332	10451	6106	110	SRR2736266_megahit.fa	10451	6106	110		SRR2736266_spades.fa	10451	6106	110		SRR2736266_before_rr.fa	10451	6106	110
SRR2736267	PRJNA298332	1929	1223	29	SRR2736267_megahit.fa	1929	1223	29		SRR2736267_spades.fa	1929	1223	29		SRR2736267_before_rr.fa	1929	1223	29
SRR2736268	PRJNA298332	9047	5351	29	SRR2736268_megahit.fa	9047	5351	29		SRR2736268_spades.fa	9047	5351	29		SRR2736268_before_rr.fa	9047	5351	29
SRR2736269	PRJNA298332	9368	2703	893	SRR2736269_megahit.fa	9368	2703	893		SRR2736269_spades.fa	9368	2703	893		SRR2736269_before_rr.fa	9368	2703	893
SRR2736270	PRJNA298332	9994	5888	4	SRR2736270_megahit.fa	9994	5888	4		SRR2736270_spades.fa	9994	5888	4		SRR2736270_before_rr.fa	9994	5888	4
SRR2736271	PRJNA298332	9368	2703	893	SRR2736271_megahit.fa	9368	2703	893		SRR2736271_spades.fa	9368	2703	893		SRR2736271_before_rr.fa	9368	2703	893
SRR2736272	PRJNA298332	9368	2703	893	SRR2736272_megahit.fa	9368	2703	893		SRR2736272_spades.fa	9368	2703	893		SRR2736272_before_rr.fa	9368	2703	893
SRR2736273	PRJNA298332	10128	5942	563	SRR2736273_megahit.fa	10128	5942	563		SRR2736273_spades.fa	10128	5942	563		SRR2736273_before_rr.fa	10128	5942	563
SRR2736274	PRJNA298332	10128	5942	563	SRR2736274_megahit.fa	10128	5942	563		SRR2736274_spades.fa	10128	5942	563		SRR2736274_before_rr.fa	10128	5942	563
SRR2736275	PRJNA298332	6765	2078	29	SRR2736275_megahit.fa	6765	2078	29		SRR2736275_spades.fa	6765	2078	29		SRR2736275_before_rr.fa	6765	2078	29
SRR2736276	PRJNA298332	10451	6106	110	SRR2736276_megahit.fa	10451	6106	110		SRR2736276_spades.fa	10451	6106	110		SRR2736276_before_rr.fa	10451	6106	110
SRR2736277	PRJNA298332	3158	1914	110	SRR2736277_megahit.fa	3158	1914	110		SRR2736277_spades.fa	3158	1914	110		SRR2736277_before_rr.fa	3158	1914	110

SRR2736278	PRJNA298332	10567	6175	29	SRR2736278_megahit.fa	10567	6175	29		SRR2736278_spades.fa	10567	6175	29	SRR2736278_before_rr.fa	10567	6175	29
SRR2736279	PRJNA298332	3149	1903	110	SRR2736279_megahit.fa	3149	1903	110		SRR2736279_spades.fa	3149	1903	110	SRR2736279_before_rr.fa	3149	1903	110
SRR2736280	PRJNA298332	1407	908	110	SRR2736280_megahit.fa	1407	908	110		SRR2736280_spades.fa	1407	908	110	SRR2736280_before_rr.fa	1407	908	110
SRR2736281	PRJNA298332	2400	1489	563	SRR2736281_megahit.fa	2400	1489	563		SRR2736281_spades.fa	2400	1489	563	SRR2736281_before_rr.fa	2400	1489	563
SRR2736282	PRJNA298332	10567	6175	29	SRR2736282_megahit.fa	-	-	29	missing	SRR2736282_spades.fa	10567	6175	29	SRR2736282_before_rr.fa	10567	6175	29
SRR2736283	PRJNA298332	10567	6175	29	SRR2736283_megahit.fa	10567	6175	29		SRR2736283_spades.fa	10567	6175	29	SRR2736283_before_rr.fa	10567	6175	29
SRR2736284	PRJNA298332	10567	6175	29	SRR2736284_megahit.fa	10567	6175	29		SRR2736284_spades.fa	10567	6175	29	SRR2736284_before_rr.fa	10567	6175	29
SRR2736285	PRJNA298332	10567	6175	29	SRR2736285_megahit.fa	10567	6175	29		SRR2736285_spades.fa	10567	6175	29	SRR2736285_before_rr.fa	10567	6175	29
SRR2736286	PRJNA298332	10567	6175	29	SRR2736286_megahit.fa	10567	6175	29		SRR2736286_spades.fa	10567	6175	29	SRR2736286_before_rr.fa	10567	6175	29
SRR2736287	PRJNA298332	10567	6175	29	SRR2736287_megahit.fa	10567	6175	29		SRR2736287_spades.fa	10567	6175	29	SRR2736287_before_rr.fa	10567	6175	29
SRR2736288	PRJNA298332	10567	6175	29	SRR2736288_megahit.fa	10567	6175	29		SRR2736288_spades.fa	10567	6175	29	SRR2736288_before_rr.fa	10567	6175	29
SRR2736289	PRJNA298332	10568	5829	4	SRR2736289_megahit.fa	10568	5829	4		SRR2736289_spades.fa	10568	5829	4	SRR2736289_before_rr.fa	10568	5829	4
SRR2736290	PRJNA298332	7571	2763	29	SRR2736290_megahit.fa	-	-	29	missing	SRR2736290_spades.fa	7571	2763	29	SRR2736290_before_rr.fa	7571	2763	29
SRR2736291	PRJNA298332	10567	6175	29	SRR2736291_megahit.fa	10567	6175	29		SRR2736291_spades.fa	10567	6175	29	SRR2736291_before_rr.fa	10567	6175	29
SRR2736292	PRJNA298332	10567	6175	29	SRR2736292_megahit.fa	10567	6175	29		SRR2736292_spades.fa	10567	6175	29	SRR2736292_before_rr.fa	10567	6175	29
SRR2736293	PRJNA298332	9368	2703	893	SRR2736293_megahit.fa	9368	2703	893		SRR2736293_spades.fa	9368	2703	893	SRR2736293_before_rr.fa	9368	2703	893
SRR2736294	PRJNA298332	9368	2703	893	SRR2736294_megahit.fa	9368	2703	893		SRR2736294_spades.fa	9368	2703	893	SRR2736294_before_rr.fa	9368	2703	893
SRR2736295	PRJNA298332	10567	6175	29	SRR2736295_megahit.fa	10567	6175	29		SRR2736295_spades.fa	10567	6175	29	SRR2736295_before_rr.fa	10567	6175	29
SRR2736296	PRJNA298332	10567	6175	29	SRR2736296_megahit.fa	10567	6175	29		SRR2736296_spades.fa	10567	6175	29	SRR2736296_before_rr.fa	10567	6175	29
SRR2736297	PRJNA298332	10567	6175	29	SRR2736297_megahit.fa	10567	6175	29		SRR2736297_spades.fa	10567	6175	29	SRR2736297_before_rr.fa	10567	6175	29
SRR2736298	PRJNA298332	9368	2703	893	SRR2736298_megahit.fa	9368	2703	893		SRR2736298_spades.fa	9368	2703	893	SRR2736298_before_rr.fa	9368	2703	893
SRR2736299	PRJNA298332	9047	5351	29	SRR2736299_megahit.fa	9047	5351	29		SRR2736299_spades.fa	9047	5351	29	SRR2736299_before_rr.fa	9047	5351	29
SRR2736300	PRJNA298332	10814	6341	29	SRR2736300_megahit.fa	10814	6341	29		SRR2736300_spades.fa	10814	6341	29	SRR2736300_before_rr.fa	10814	6341	29
SRR2736301	PRJNA298332	10815	6342	894	SRR2736301_megahit.fa	10815	6342	894		SRR2736301_spades.fa	10815	6342	894	SRR2736301_before_rr.fa	10815	6342	894
SRR2736302	PRJNA298332	10567	6175	29	SRR2736302_megahit.fa	10567	6175	29		SRR2736302_spades.fa	10567	6175	29	SRR2736302_before_rr.fa	10567	6175	29
SRR2736303	PRJNA298332	10567	6175	29	SRR2736303_megahit.fa	-	6175	-	missing	SRR2736303_spades.fa	10567	6175	29	SRR2736303_before_rr.fa	10567	6175	29
SRR2736304	PRJNA298332	9368	2703	893	SRR2736304_megahit.fa	9368	2703	893		SRR2736304_spades.fa	9368	2703	893	SRR2736304_before_rr.fa	9368	2703	893
SRR2736305	PRJNA298332	9368	2703	893	SRR2736305_megahit.fa	9368	2703	893		SRR2736305_spades.fa	9368	2703	893	SRR2736305_before_rr.fa	9368	2703	893
SRR2736306	PRJNA298332	9047	5351	29	SRR2736306_megahit.fa	9047	5351	29		SRR2736306_spades.fa	9047	5351	29	SRR2736306_before_rr.fa	9047	5351	29
ERR1426708	PRJEB14168	495	91	32	AUSMDU00004452_megahit.fa	495	91	32		AUSMDU00004452_spades.fa	495	91	32	AUSMDU00004452_before-rr.fa	495	91	32
ERR1426709	PRJEB14168	3304	2000	4	AUSMDU00004453_megahit.fa	3304	2000	4		AUSMDU00004453_spades.fa	3304	2000	4	AUSMDU00004453_before-rr.fa	3304	2000	4
ERR1426710	PRJEB14168	556	90	122	AUSMDU00004454_megahit.fa	556	90	122		AUSMDU00004454_spades.fa	556	90	122	AUSMDU00004454_before-rr.fa	556	90	122
ERR1426711	PRJEB14168	3303	2002	165	AUSMDU00004455_megahit.fa	3303	2002	165		AUSMDU00004455_spades.fa	3303	2002	165	AUSMDU00004455_before-rr.fa	3303	2002	165
ERR1426712	PRJEB14168	1407	908	110	AUSMDU00004456_megahit.fa	1407	908	110		AUSMDU00004456_spades.fa	1407	908	110	AUSMDU00004456_before-rr.fa	1407	908	110
ERR1426713	PRJEB14168	1407	908	110	AUSMDU00004457_megahit.fa	1407	908	110		AUSMDU00004457_spades.fa	1407	908	110	AUSMDU00004457_before-rr.fa	1407	908	110
ERR1426714	PRJEB14168	1407	908	110	AUSMDU00004458_megahit.fa	1407	908	110		AUSMDU00004458_spades.fa	1407	908	110	AUSMDU00004458_before-rr.fa	1407	908	110
ERR1426715	PRJEB14168	1619	1057	21	AUSMDU00004459_megahit.fa	1619	1057	21		AUSMDU00004459_spades.fa	1619	1057	21	AUSMDU00004459_before-rr.fa	1619	1057	21
ERR1426716	PRJEB14168	3493	2111	26	AUSMDU00004460_megahit.fa	3493	2111	26		AUSMDU00004460_spades.fa	3493	2111	26	AUSMDU00004460_before-rr.fa	3493	2111	26
ERR1426717	PRJEB14168	1768	1135	446	AUSMDU00004461_megahit.fa	1768	1135	446		AUSMDU00004461_spades.fa	1768	1135	446	AUSMDU00004461_before-rr.fa	1768	1135	446
ERR1426718	PRJEB14168	225	4	4	AUSMDU00004462_megahit.fa	225	4	4		AUSMDU00004462_spades.fa	225	4	4	AUSMDU00004462_before-rr.fa	225	4	4
ERR1426719	PRJEB14168	4950	3002	110	AUSMDU00004463_megahit.fa	4950	3002	110		AUSMDU00004463_spades.fa	4950	3002	110	AUSMDU00004463_before-rr.fa	4950	3002	110
ERR1426720	PRJEB14168	4951	3003	110	AUSMDU00004464_megahit.fa	4951	3003	110		AUSMDU00004464_spades.fa	4951	3003	110	AUSMDU00004464_before-rr.fa	4951	3003	110
ERR1426721	PRJEB14168	4949	3001	29	AUSMDU00004465_megahit.fa	4949	3001	29		AUSMDU00004465_spades.fa	4949	3001	29	AUSMDU00004465_before-rr.fa	4949	3001	29
ERR1426722	PRJEB14168	2992	1808	29	AUSMDU00004466_megahit.fa	2992	1808	29		AUSMDU00004466_spades.fa	2992	1808	29	AUSMDU00004466_before-rr.fa	2992	1808	29
ERR1426723	PRJEB14168	4990	90	1044	AUSMDU00004467_megahit.fa	4990	90	1044		AUSMDU00004467_spades.fa	4990	90	1044	AUSMDU00004467_before-rr.fa	4990	90	1044
ERR1426724	PRJEB14168	4376	2683	186	AUSMDU00004468_megahit.fa	4376	2683	186		AUSMDU00004468_spades.fa	4376	2683	186	AUSMDU00004468_before-rr.fa	4376	2683	186
ERR1426725	PRJEB14168	835	566	10	AUSMDU00004469_megahit.fa	835	566	10		AUSMDU00004469_spades.fa	835	566	10	AUSMDU00004469_before-rr.fa	835	566	10
ERR1426726	PRJEB14168	912	318	74	AUSMDU00004470_megahit.fa	912	318	74		AUSMDU00004470_spades.fa	912	318	74	AUSMDU00004470_before-rr.fa	912	318	74
ERR1426727	PRJEB14168	3305	2001	29	AUSMDU00004471_megahit.fa	3305	2001	29		AUSMDU00004471_spades.fa	3305	2001	29	AUSMDU00004471_before-rr.fa	3305	2001	29
ERR1426728	PRJEB14168	621	90	18	AUSMDU00004472_megahit.fa	621	90	18		AUSMDU00004472_spades.fa	621	90	18	AUSMDU00004472_before-rr.fa	621	90	18
ERR1426729	PRJEB14168	6354	3751	470	AUSMDU00004473_megahit.fa	6354	3751	470		AUSMDU00004473_spades.fa	6354	3751	470	AUSMDU00004473_before-rr.fa	6354	3751	470
ERR1426730	PRJEB14168	4822	1903	29	AUSMDU00004474_megahit.fa	4822	1903	29		AUSMDU00004474_spades.fa	4822	1903	29	AUSMDU00004474_before-rr.fa	4822	1903	29
ERR1426731	PRJEB14168	8917	5170	467	AUSMDU00004475_megahit.fa	8917	5170	467		AUSMDU00004475_spades.fa	8917	5170	467	AUSMDU00004475_before-rr.fa	8917	5170	467
ERR1426732	PRJEB14168	1424	917	10	AUSMDU00004476_megahit.fa	1424	917	10		AUSMDU00004476_spades.fa	1424	917	10	AUSMDU00004476_before-rr.fa	1424	917	10
ERR1426733	PRJEB14168	1407	908	110	AUSMDU00004477_megahit.fa	1407	908	110		AUSMDU00004477_spades.fa	1407	908	110	AUSMDU00004477_before-rr.fa	1407	908	110
ERR1426734	PRJEB14168	1752	1119	193	AUSMDU00004478_megahit.fa	1752	1119	193		AUSMDU00004478_spades.fa	1752	1119	193	AUSMDU00004478_before-rr.fa	1752	1119	193
ERR1426735	PRJEB14168	1422	915	21	AUSMDU00004479_megahit.fa	1422	915	21		AUSMDU00004479_spades.fa	1422	915	21	AUSMDU00004479_before-rr.fa	1422	915	21
ERR1426736	PRJEB14168	1572	1022	6	AUSMDU00004480_megahit.fa	1572	1022	6		AUSMDU00004480_spades.fa	1572	1022	6	AUSMDU00004480_before-rr.fa	1572	1022	6
ERR1426737	PRJEB14168	285	232	4	AUSMDU00004481_megahit.fa	285	232	4		AUSMDU00004481_spades.fa	285	232	4	AUSMDU00004481_before-rr.fa	285	232	4
ERR1426738	PRJEB14168	2400	1489	563	AUSMDU00004482_megahit.fa	2400	1489	563		AUSMDU00004482_spades.fa	2400	1489	563	AUSMDU00004482_before-rr.fa	2400	1489	563
ERR1426739	PRJEB14168	9368	2703	893	AUSMDU00004483_megahit.fa	9368	2703	893		AUSMDU00004483_spades.fa	9368	2703	893	AUSMDU00004483_before-rr.fa	9368	2703	893
ERR1426740	PRJEB14168	10193	4097	579	AUSMDU00004484_megahit.fa	10193	4097	579		AUSMDU00004484_spades.fa	10193	4097	579	AUSMDU00004484_before-rr.fa	10193	4097	579
ERR1426741	PRJEB14168	356	61	27	AUSMDU00004485_megahit.fa	356	61	27		AUSMDU00004485_spades.fa	356	61	27	AUSMDU00004485_before-rr.fa	356	61	27
ERR1426742	PRJEB14168	299	2	27	AUSMDU00004486_megahit.fa	299	2	27		AUSMDU00004486_spades.fa	299	2	27	AUSMDU00004486_before-rr.fa	299	2	27
ERR1426743	PRJEB14168	7175	4292	35	AUSMDU00004487_megahit.fa	7175	4292	35		AUSMDU00004487_spades.fa	7175	4292	35	AUSMDU00004487_before-rr.fa	7175	4292	35
ERR1426744	PRJEB14168	9650	787	4	AUSMDU00004488_megahit.fa	9650	787	4		AUSMDU00004488_spades.fa	9650	787	4	AUSMDU00004488_before-rr.fa	9650	787	4
ERR1426745	PRJEB14168	7577	30	1432	AUSMDU00004489_megahit.fa	7577	30	1432		AUSMDU00004489_spades.fa	7577	30	1432	AUSMDU00004489_before-rr.fa	7577	30	1432
ERR1426746	PRJEB14168	10998	5936	1783	AUSMDU00004490_megahit.fa	10998	5936	1783		AUSMDU00004490_spades.fa	10998	5936	1783	AUSMDU00004490_before-rr.fa	10998	5936	1783
ERR1426747	PRJEB14168	7753	4659	4	AUSMDU00004491_megahit.fa	7753	4659	4		AUSMDU00004491_spades.fa	7753	4659	4	AUSMDU00004491_before-rr.fa	7753	4659	4
ERR1426748	PRJEB14168	1407	908	110	AUSMDU00004492_megahit.fa	1407	908	110		AUSMDU00004492_spades.fa	1407	908	110	AUSMDU00004492_before-rr.fa	1407	908	110
ERR1426749	PRJEB14168	4186	2569	241	AUSMDU00004493_megahit.fa	4186	2569	241		AUSMDU00004493_spades.fa	4186	2569	241	AUSMDU00004493_before-rr.fa	4186	2569	241
ERR1426750	PRJEB14168	7803	161	349	AUSMDU00004494_megahit.fa	7803	161	349		AUSMDU00004494_spades.fa	7803	161	349	AUSMDU00004494_before-rr.fa	7803	161	349
ERR1426751	PRJEB14168	9166	5466	29	AUSMDU00004495_megahit.fa	9166	5466	29		AUSMDU00004495_spades.fa	9166	5466	29	AUSMDU00004495_before-rr.fa	9166	5466	29
ERR1426752	PRJEB14168	11520	6746	19	AUSMDU00004496_megahit.fa	11520	6746	19		AUSMDU00004496_spades.fa	11520	6746	19	AUSMDU00004496_before-rr.fa	11520	6746	19
ERR1426753	PRJEB14168	402	294	21	AUSMDU00004497_megahit.fa	402	294	21		AUSMDU00004497_spades.fa	402	294	21	AUSMDU00004497_before-rr.fa	402	294	21
ERR1426754	PRJEB14168	217	158	4	AUSMDU00004498_megahit.fa	217	158	4		AUSMDU00004498_spades.fa	217	158	4	AUSMDU00004498_before-rr.fa	217	158	4
ERR1426755	PRJEB14168	4244	1808	893	AUSMDU00004499_megahit.fa	4244	1808	893		AUSMDU00004499_spades.fa	4244	1808	893	AUSMDU00004499_before-rr.fa	4244	1808	893
ERR1426756	PRJEB14168	6950	4150	1341	AUSMDU00004500_megahit.fa	6950	4150	1341		AUSMDU00004500_spades.fa	6950	4150	1341	AUSMDU00004500_before-rr.fa	6950	4150	1341
ERR1426757	PRJEB14168	5624	90	953	AUSMDU00004501_megahit.fa	5624	90	953		AUSMDU00004501_spades.fa	5624	90	953	AUSMDU00004501_before-rr.fa	5624	90	953

SAMPLE_ID	STUDY	READS	YIELD	GC	MIN_LEN	AVG_LEN	MAX_LEN	MODE_LEN	PHRED	AVG_QUAL	DEPTH	MEGAHIT_ID	CONTIGS	SIZE	OK	Ns	GAPS	MIN	AVG	MAX	N50	SPADES_ID	CONTIGS	SIZE	OK	Ns	GAPS	MIN	AVG	MAX	N50
ERR191730	PRJEB2999	4596890	459689000	51.9	100	100	100	100	33	35	202x	ERR191730_megahit.fa	188	2138715	2138715	0	0	500	11376	67545	36060	ERR191730_spades.fa	139	2156462	2156462	0	0	98	15514	108574	48768
ERR191731	PRJEB2999	7120646	712064600	51.9	100	100	100	100	33	35.1	313x	ERR191731_megahit.fa	158	2129597	2129597	0	0	530	13478	105283	37120	ERR191731_spades.fa	155	2167893	2167893	0	0	98	13986	208318	47518
ERR191732	PRJEB2999	3621838	362183800	51.9	100	100	100	100	33	35	159x	ERR191732_megahit.fa	166	2122954	2122954	0	0	500	12788	67539	36060	ERR191732_spades.fa	131	2146700	2146700	0	0	102	16387	189751	47744
ERR191733	PRJEB2999	3663482	366348200	51.8	100	100	100	100	33	35.1	161x	ERR191733_megahit.fa	162	2127410	2127410	0	0	530	13132	68377	35401	ERR191733_spades.fa	147	2157581	2157581	0	0	98	14677	128525	44403
ERR191734	PRJEB2999	4054914	405491400	51.7	100	100	100	100	33	35.1	178x	ERR191734_megahit.fa	165	2121568	2121568	0	0	500	12857	67543	34015	ERR191734_spades.fa	163	2153213	2153213	0	0	98	13209	152282	44155
ERR191735	PRJEB2999	5961108	596110800	51.9	100	100	100	100	33	35	262x	ERR191735_megahit.fa	159	2123466	2123466	0	0	509	13355	107835	35447	ERR191735_spades.fa	183	2162888	2162888	0	0	98	11819	173534	47595
ERR191736	PRJEB2999	5031372	503137200	51.7	100	100	100	100	33	35.1	221x	ERR191736_megahit.fa	165	2121895	2121895	0	0	500	12859	67544	35447	ERR191736_spades.fa	162	2155355	2155355	0	0	98	13304	173792	43212
ERR191737	PRJEB2999	5842272	584227200	51.9	100	100	100	100	33	35.1	256x	ERR191737_megahit.fa	161	2104746	2104746	0	0	510	13072	172708	34831	ERR191737_spades.fa	146	2144684	2144684	0	0	98	14689	207731	47514
ERR191738	PRJEB2999	3670272	367027200	51.8	100	100	100	100	33	35	161x	ERR191738_megahit.fa	163	2073202	2073202	0	0	507	12719	105300	36225	ERR191738_spades.fa	106	2082005	2082005	0	0	104	19641	173517	49446
ERR191739	PRJEB2999	3927866	392786600	52	100	100	100	100	33	35.1	172x	ERR191739_megahit.fa	160	2067336	2067336	0	0	537	12920	95102	35291	ERR191739_spades.fa	165	2102627	2102627	0	0	98	12743	172831	43394
ERR191740	PRJEB2999	7196718	719671800	51.9	100	100	100	100	33	35.2	316x	ERR191740_megahit.fa	166	2132198	2132198	0	0	503	12844	108265	35458	ERR191740_spades.fa	163	2166646	2166646	0	0	98	13292	152708	47749
ERR191741	PRJEB2999	5788932	578893200	51.9	100	100	100	100	33	35.1	254x	ERR191741_megahit.fa	163	2134135	2134135	0	0	503	13092	108268	35494	ERR191741_spades.fa	171	2167532	2167532	0	0	98	12675	152708	48639
ERR191746	PRJEB2999	7151404	715140400	51.8	100	100	100	100	33	35.2	314x	ERR191746_megahit.fa	165	2122347	2122347	0	0	500	12862	105280	35447	ERR191746_spades.fa	152	2156915	2156915	0	0	98	14190	172950	47242
ERR191747	PRJEB2999	3714522	371452200	52	100	100	100	100	33	35.1	163x	ERR191747_megahit.fa	188	2088881	2088881	0	0	507	11111	105296	35465	ERR191747_spades.fa	152	2100394	2100394	0	0	98	13818	172963	47110
ERR191748	PRJEB2999	4640150	464015000	51.8	100	100	100	100	33	35.1	204x	ERR191748_megahit.fa	164	2123211	2123211	0	0	500	12946	105293	35447	ERR191748_spades.fa	141	2154787	2154787	0	0	98	15282	191263	48627
ERR191749	PRJEB2999	4854092	485409200	51.8	100	100	100	100	33	35.1	213x	ERR191749_megahit.fa	162	2124359	2124359	0	0	505	13113	113809	35863	ERR191749_spades.fa	156	2157790	2157790	0	0	98	13831	207774	48865
ERR191750	PRJEB2999	7848930	784893000	51.8	100	100	100	100	33	35.1	345x	ERR191750_megahit.fa	166	2123451	2123451	0	0	500	12791	105298	35447	ERR191750_spades.fa	144	2159239	2159239	0	0	98	14994	207975	48721
ERR191751	PRJEB2999	4618014	461801400	51.9	100	100	100	100	33	35.1	203x	ERR191751_megahit.fa	193	2137810	2137810	0	0	505	11076	67849	27311	ERR191751_spades.fa	162	2150581	2150581	0	0	98	13275	196720	44518
ERR191752	PRJEB2999	6199800	619980000	51.8	100	100	100	100	33	35.1	272x	ERR191752_megahit.fa	165	2122162	2122162	0	0	500	12861	108327	36712	ERR191752_spades.fa	172	2162430	2162430	0	0	98	12572	207962	46837
ERR191753	PRJEB2999	4474248	447424800	51	100	100	100	100	33	35.1	196x	ERR191753_megahit.fa	163	2122240	2122240	0	0	502	13019	105244	34611	ERR191753_spades.fa	152	2152815	2152815	0	0	98	14163	173631	48736
ERR191754	PRJEB2999	4344046	434404600	51.7	100	100	100	100	33	35.1	191x	ERR191754_megahit.fa	165	2124161	2124161	0	0	500	12873	105280	35447	ERR191754_spades.fa	129	2152508	2152508	0	0	98	16686	207972	43132
ERR191755	PRJEB2999	3254600	325460000	51.8	100	100	100	100	33	34.9	143x	ERR191755_megahit.fa	168	2128274	2128274	0	0	500	12668	105239	30498	ERR191755_spades.fa	102	2138994	2138994	0	0	379	20970	172632	46733
ERR191756	PRJEB2999	3126182	312618200	51.7	100	100	100	100	33	35	137x	ERR191756_megahit.fa	169	2125571	2125571	0	0	507	12577	105278	35384	ERR191756_spades.fa	126	2146314	2146314	0	0	102	17034	207756	48614
ERR191757	PRJEB2999	4178670	417867000	51.7	100	100	100	100	33	35.1	183x	ERR191757_megahit.fa	170	2124691	2124691	0	0	500	12498	67266	35302	ERR191757_spades.fa	145	2155320	2155320	0	0	98	14864	207979	46118
ERR191758	PRJEB2999	3621414	362141400	51.7	100	100	100	100	33	35	159x	ERR191758_megahit.fa	166	2124751	2124751	0	0	500	12799	105278	35384	ERR191758_spades.fa	93	2140201	2140201	0	0	98	23012	207784	43338
ERR191759	PRJEB2999	3753938	375393800	51.7	100	100	100	100	33	35.1	165x	ERR191759_megahit.fa	166	2125219	2125219	0	0	500	12802	67543	34862	ERR191759_spades.fa	117	2149035	2149035	0	0	98	18367	127775	43176
ERR191760	PRJEB2999	3935142	393514200	51.6	100	100	100	100	33	35	173x	ERR191760_megahit.fa	164	2124604	2124604	0	0	500	12954	105278	30731	ERR191760_spades.fa	152	2154021	2154021	0	0	98	14171	193260	42088
ERR191761	PRJEB2999	4046338	404633800	52	100	100	100	100	33	35.1	177x	ERR191761_megahit.fa	169	2071510	2071510	0	0	509	12257	105258	30494	ERR191761_spades.fa	163	2106817	2106817	0	0	98	12925	173201	47040
ERR191762	PRJEB2999	3799268	379926800	51.5	100	100	100	100	33	35.1	167x	ERR191762_megahit.fa	166	2167169	2167169	0	0	500	13055	108336	35864	ERR191762_spades.fa	139	2191632	2191632	0	0	98	15767	176202	44542
ERR191763	PRJEB2999	3939662	393966200	51.8	100	100	100	100	33	35	173x	ERR191763_megahit.fa	166	2133455	2133455	0	0	524	12852	105263	30518	ERR191763_spades.fa	149	2163013	2163013	0	0	98	14516	172940	47640
ERR191764	PRJEB2999	11148772	1114877200	51.8	100	100	100	100	33	35.1	490x	ERR191764_megahit.fa	169	2126814	2126814	0	0	500	12584	67544	35387	ERR191764_spades.fa	153	2155850	2155850	0	0	98	14090	207968	52951
ERR191765	PRJEB2999	8513900	851390000	51.7	100	100	100	100	33	35.1	374x	ERR191765_megahit.fa	168	2130498	2130498	0	0	519	12681	67544	37120	ERR191765_spades.fa	165	2169716	2169715	1	0	98	13149	152508	55147
ERR191766	PRJEB2999	7042696	704269600	51.8	100	100	100	100	33	35.1	309x	ERR191766_megahit.fa	164	2121039	2121039	0	0	500	12933	67544	34015	ERR191766_spades.fa	160	2159652	2159652	0	0	98	13497	152519	48627
ERR191767	PRJEB2999	3667728	366772800	52	100	100	100	100	33	35.1	161x	ERR191767_megahit.fa	174	2075917	2075917	0	0	507	11930	95095	35485	ERR191767_spades.fa	144	2099940	2099940	0	0	101	14582	176264	52589
ERR191768	PRJEB2999	5504190	550419000	51.8	100	100	100	100	33	35.1	242x	ERR191768_megahit.fa	166	2122619	2122619	0	0	500	12786	108299	36060	ERR191768_spades.fa	147	2155750	2155750	0	0	98	14664	207911	47297
ERR191769	PRJEB2999	3659636	365963600	51.4	100	100	100	100	33	35.1	160x	ERR191769_megahit.fa	155	2120454	2120454	0	0	529	13680	105225	37628	ERR191769_spades.fa	165	2154426	2154426	0	0	98	13057	173284	43162
ERR191770	PRJEB2999	3708338	370833800	51.8	100	100	100	100	33	35.1	163x	ERR191770_megahit.fa	173	2126857	2126857	0	0	500	12293	67545	34015	ERR191770_spades.fa	135	2153961	2153961	0	0	98	15955	218563	55272
ERR191771	PRJEB2999	3512872	351287200	51.9	100	100	100	100	33	35	154x	ERR191771_megahit.fa	160	2125919	2125919	0	0	515	13286	172701	35465	ERR191771_spades.fa	105	2134985	2134985	0	0	109	20333	175958	45652
ERR191772	PRJEB2999	3958930	395893000	51.8	100	100	100	100	33	35.1	174x	ERR191772_megahit.fa	167	2123459	2123459	0	0	500	12715	172654	35447	ERR191772_spades.fa	154	2151557	2151557	0	0	98	13971	191144	45640
ERR191773	PRJEB2999	5082240	508224000	52	100	100	100	100	33	35	223x	ERR191773_megahit.fa	259	2141981	2141981	0	0	500	8270	105246	23350	ERR191773_spades.fa	161	2110007	2110007	0	0	98	13105	196762	44693
ERR191774	PRJEB2999	3760098	376009800	51.8	100	100	100	100	33	35.1	165x	ERR191774_megahit.fa	166	2125505	2125505	0	0	500	12804	172690	36060	ERR191774_spades.fa	147	2150891	2150891	0	0	98	14631	207716	47004
ERR191775	PRJEB2999	3794942	379494200	51.8	100	100	100	100	33	35	166x	ERR191775_megahit.fa	164	2125944	2125944	0	0	500	12963	108303	36060	ERR191775_spades.fa	134	2148754	2148754	0	0	102	16035	108491	49732
ERR191776	PRJEB2999	4115432	411543200	51.9	100	100	100	100	33	35.1	181x	ERR191776_megahit.fa	165	2123715	2123715	0	0	500	12871	172685	36060	ERR191776_spades.fa	149	2151005	2151004	1	0	102	14436	173704	45653
ERR191777	PRJEB2999	3949456	394945600	51.7	100	100	100	100	33	35	173x	ERR191777_megahit.fa	167	2124139	2124139	0	0	500	12719	108299	36060	ERR191777_spades.fa	156	2151152	2151152	0	0	98	13789	152519	46859
ERR191778	PRJEB2999	6186750	618675000	51.8	100	100	100	100	33	35.1	272x	ERR191778_megahit.fa	167	2123113	2123113	0	0	507	12713	108289	35447	ERR191778_spades.fa	150	2153720	2153720	0	0	98	14358	207733	47364
ERR191779	PRJEB2999	4361412	436141200	51.9	100	100	100	100	33	35.1	191x	ERR191779_megahit.fa	156	2117418	2117418	0	0	508	13573	108284	30729	ERR191779_spades.fa	138	2148469	2148469	0	0	98	15568	153937	49270
ERR191780	PRJEB2999	3831580	383158000	51.8	100	100	100	100	33	35.1	168x	ERR191780_megahit.fa	168	2125080	2125080	0	0	504	12649	172654	36060	ERR191780_spades.fa	127	2147840	2147840	0	0	98	16912	207680	55660
ERR191781	PRJEB2999	3539522	353952200	51.9	100	100	100	100	33	35	155x	ERR191781_megahit.fa	162	2125896	2125896	0	0	520	13122	67540	36836	ERR191781_spades.fa	138	2150098	2150098	0	0	98	15580	175419	48993
ERR191782	PRJEB2999	3743504	374350400	51.8	100	100	100	100	33	35	164x	ERR191782_megahit.fa	159	2120914	2120914	0	0	500	13339	67543	35447	ERR191782_spades.fa	136	2150557	2150557	0	0	101	15812	176209	47375
ERR191783	PRJEB2999	3986548	398654800	51.7	100	100	100	100	33	35	175x	ERR191783_megahit.fa	159	2124603	2124603	0	0	515	13362	172718	35404	ERR191783_spades.fa	131	2150553	2150553	0	0	98	16416	207747	49240
ERR191784	PRJEB2999	4654772	465477200	51.7	100	100	100	100	33	35	204x	ERR191784_megahit.fa	169	2123277	2123277	0	0	500	12563	105265	34015	ERR191784_spades.fa	150	2153874	2153874	0	0	98	14359	207902	47021
ERR191785	PRJEB2999	4048280	404828000	51.8	100	100	100	100	33	35	178x	ERR191785_megahit.fa	164	2123639	2123639	0	0	525	12949	105246	31854	ERR191785_spades.fa	137	2155550	2155550	0	0	98	15733	175994	45686
ERR191786	PRJEB2999	8215558	821555800	51.7	100	100	100	100	33	35.2	361x	ERR191786_megahit.fa	166	2122195	2122195	0	0	500	12784	105286	35447	ERR191786_spades.fa	149	2154835	2154835	0	0	98	14461	207963	52771
ERR191787	PRJEB2999	4247452	424745200	51.7	100	100	100	100	33	35	186x	ERR191787_megahit.fa	163	2128892	2128892	0	0	500	13060	68457	36064	ERR191787_spades.fa	159	2159186	2159186	0	0	98	13579	188911	55579
ERR191788	PRJEB2999	4014274	401427400	51.7	100	100	100	100	33	35	176x	ERR191788_megahit.fa	167	2123776	2123776	0	0	500	12717	67542	35394	ERR191788_spades.fa	151	2156312	2156312	0	0	98	14280	193317	43757
ERR191789	PRJEB2999	6082130	608213000	52	100	100	100	100	33	35.1	267x	ERR191789_megahit.fa	168	2069929	2069929	0	0	514	12321	105262	30494	ERR191789_spades.fa	160	2109668	2109668	0	0	98	13185	207846	46665
ERR191790	PRJEB2999	6691256	669125600	51.7	100	100	100	100	33	35	294x	ERR191790_megahit.fa	165	2122142	2122142	0	0	500	12861	105286	35384	ERR191790_spades.fa	152	2155296	2155296	0	0	98	14179	207970	44236
ERR191791	PRJEB2999	4208566	420856600	51.9	100	100	100	100	33	35.1	185x	ERR191791_megahit.fa	152	2068306	2068306	0	0	541	13607	105288	36091	ERR191791_spades.fa	146	2095400	2095400	0	0	98	14352	176246	53346
ERR191792	PRJEB2999	4376650	437665000	51.7	100	100	100	100	33	35.1	192x	ERR191792_megahit.fa	169	2123053	2123053	0	0	500	12562	67541	35447	ERR191792_spades.fa	145	2151544	2151544	0	0	98	14838	207981	44327
ERR191793	PRJEB2999	4078976	407897600	51.7	100	100	100	100	33	35.2	179x	ERR191793_megahit.fa	181	2124400	2124400	0	0	506	11737	67549	30236	ERR191793_spades.fa	147	2142192	2142192	0	0	98	14572	172708	44714
ERR191794	PRJEB2999	3534168	353416800	51.7	100	100	100	100	33	35	155x	ERR191794_megahit.fa	166	2122453	2122453	0	0	500	12785	67541	35447	ERR191794_spades.fa	99	2129281	2129281	0	0	98	21507	189238	48753
ERR191795	PRJEB2999	3718062	371806200	51.7	100	100	100	100	33	35.1	163x	ERR191795_megahit.fa	165	2123378	2123378	0	0	500	12868	105304	35447	ERR191795_spades.fa	127	2148740	2148740	0	0	98	16919	207805	47509
ERR191796	PRJEB2999	3889168	388916800	51.8	100	100	100	100	33	35	171x	ERR191796_megahit.fa	164	2121807	2121807	0	0	500	12937	67544	37224	ERR191796_spades.fa	128	2149712	2149712	0	0	116	16794	189087	49035
ERR191797	PRJEB2999	7986198	798619800	51.7	100	100	100	100	33	35.1	351x	ERR191797_megahit.fa	167	2123779	2123779	0	0	500	12717	67545	35310	ERR191797_spades.fa	151	2162857	2162857	0	0	98	14323	207981	47839
ERR191798	PRJEB2999	5209026	520902600	51.8	100	100	100	100	33	35.1	229x	ERR191798_megahit.fa	199	2168903	2168903	0	0	500	10899	67541	27555	ERR191798_spades.fa	159	2153682	2153682	0	0	99	13545	108585	55597
ERR191799	PRJEB2999	5157586	515758600	51.8	100	100	100	100	33	35.1	226x	ERR191799_megahit.fa	173	2134274	2134274	0	0	500	12336	67982	33971	ERR191799_spades.fa	182	2166016	2166016	0	0	98	11901	108981	49287
ERR191800	PRJEB2999	3116254	311625400	51.7	100	100	100	100	33	35	137x	ERR191800_megahit.fa	166	2129753	2129753	0	0	531	12829	113807	36787	ERR191800_spades.fa	99	2138583	2138583	0	0	102	21601	208050	47420
ERR191801	PRJEB2999	5739328	573932800	51.7	100	100	100	100	33	35	252x	ERR191801_megahit.fa	159	2156613	2156613	0	0	509	13563	151785	35648	ERR191801_spades.fa	145	2184463	2184463	0	0	98	15065	170297	46720
ERR191802	PRJEB2999	8851502	885150200	51.7	100	100	100	100	33	35.1	389x	ERR191802_megahit.fa	165	2128796	2128796	0	0	507	12901	126149	36787	ERR191802_spades.fa	137	2156884	2156884	0	0	98	15743	152466	49540
ERR191803	PRJEB2999	8111370	811137000	52	100	100	100	100	33	35.1	356x	ERR191803_megahit.fa	152	2066897	2066897	0	0	537	13598	105147	35521	ERR191803_spades.fa	176	2103617	2103617	0	0	98	11952	207653	46934
ERR191804	PRJEB2999	4026850	402685000	51.7	100	100	100	100	33	35.1	177x	ERR191804_megahit.fa	165	2121765	2121765	0	0	500	12859	108326	30313	ERR191804_spades.fa	138	2150546	2150546	0	0	101	15583	152250	46645
ERR191805	PRJEB2999	3756450	375645000	51.7	100	100	100	100	33	35.1	165x	ERR191805_megahit.fa	181	2136146	2136146	0	0	500	11801	67543	35447	ERR191805_spades.fa	147	2151963	2151963	0	0	98	14639	193209	51603
ERR191806	PRJEB2999	4312310	431231000	51.7	100	100	100	100	33	35.2	189x	ERR191806_megahit.fa	166	2124228	2124228	0	0	500	12796	67542	35447	ERR191806_spades.fa	141	2154173	2154173	0	0	98	15277	153974	45181
ERR191807	PRJEB2999	3890720	389072000	51.9	100	100	100	100	33	35.1	171x	ERR191807_megahit.fa	168	2124565	2124565	0	0	533	12646	105245	31854	ERR191807_spades.fa	165	2158779	2158779	0	0	100	13083	207829	46935
ERR191808	PRJEB2999	5253178	525317800	51.8	100	100	100	100	33	35.1	231x	ERR191808_megahit.fa	166	2123081	2123081	0	0	500	12789	105264	36060	ERR191808_spades.fa	149	2155132	2155132	0	0	98	14463	207942	47992
ERR191809	PRJEB2999	4470312	447031200	51.8	100	100	100	100	33	35.1	196x	ERR191809_megahit.fa	253	2154594	2154594	0	0	501	8516	67266	25383	ERR191809_spades.fa	207	2174595	2174595	0	0	98	10505	175602	42314
ERR191810	PRJEB2999	3825448	382544800	51.7	100	100	100	100	33	35.1	168x	ERR191810_megahit.fa	186	2160961	2160961	0	0	500	11618	105261	36060	ERR191810_spades.fa	164	2165736	2165736	0	0	98	13205	218498	46624
ERR191811	PRJEB2999	8741912	874191200	51.7	100	100	100	100	33	35	384x	ERR191811_megahit.fa	154	2107010	2107010	0	0	523	13681	172657	34575	ERR191811_spades.fa	170	2147661	2147661	0	0	98	12633	172841	44576
ERR191812	PRJEB2999	5673144	567314400	51.9	100	100	100	100	33	35.1	249x	ERR191812_megahit.fa	167	2074457	2074457	0	0	511	12421	105296	30820	ERR191812_spades.fa	145	2107996	2107996	0	0	98	14537	208032	50949
ERR191813	PRJEB2999	4437784	443778400	52	100	100	100	100	33	35.1	195x	ERR191813_megahit.fa	166	2075310	2075310	0	0	520	12501	105272	34792	ERR191813_spades.fa	154	2104805	2104805	0	0	98	13667	173489	41380
ERR191814	PRJEB2999	8716244	871624400	51.8	100	100	100	100	33	35	383x	ERR191814_megahit.fa	170	2123217	2123217	0	0	500	12489	105290	34015	ERR191814_spades.fa	176	2159621	2159621	0	0	98	12270	207968	50080
ERR191815	PRJEB2999	5756120	575612000	51.7	100	100	100	100	33	35	253x	ERR191815_megahit.fa	331	2174762	2174762	0	0	501	6570	101652	18622	ERR191815_spades.fa	190	2180435	2180435	0	0	98	11475	207906	55576
ERR191816	PRJEB2999	3436542	343654200	51.7	100	100	100	100	33	35	151x	ERR191816_megahit.fa	167	2131323	2131323	0	0	500	12762	67542	35865	ERR191816_spades.fa	110	2142987	2142987	0	0	99	19481	110236	46687
ERR191817	PRJEB2999	4431070	443107000	51.7	100	100	100	100	33	35.1	194x	ERR191817_megahit.fa	178	2133674	2133674	0	0	500	11986	67544	35403	ERR191817_spades.fa	149	2162161	2162161	0	0	98	14511	152519	44405
ERR191818	PRJEB2999	5303724	530372400	51.7	100	100	100	100	33	35.1	233x	ERR191818_megahit.fa	164	2121205	2121205	0	0	500	12934	67544	35384	ERR191818_spades.fa	131	2156927	2156927	0	0	98	16465	189239	52936
ERR191819	PRJEB2999	4189990	418999000	51.2	100	100	100	100	33	35	184x	ERR191819_megahit.fa	171	2115006	2115006	0	0	505	12368	72472	30500	ERR191819_spades.fa	154	2143443	2143443	0	0	98	13918	175853	43114
ERR191820	PRJEB2999	3854860	385486000	51.5	100	100	100	100	33	35	169x	ERR191820_megahit.fa	163	2121662	2121662	0	0	500	13016	67545	35583	ERR191820_spades.fa	136	2147826	2147826	0	0	99	15792	152518	48621
ERR191821	PRJEB2999	4608620	460862000	51.7	100	100	100	100	33	35.1	202x	ERR191821_megahit.fa	155	2073895	2073895	0	0	535	13379	105293	36787	ERR191821_spades.fa	140	2104837	2104837	0	0	98	15034	208035	55179
ERR191822	PRJEB2999	4638610	463861000	51.7	100	100	100	100	33	35.1	204x	ERR191822_megahit.fa	164	2120919	2120919	0	0	500	12932	67546	34015	ERR191822_spades.fa	152	2153334	2153334	0	0	98	14166	189238	47717
ERR191823	PRJEB2999	8453366	845336600	51.8	100	100	100	100	33	35	371x	ERR191823_megahit.fa	158	2123607	2123607	0	0	500	13440	68372	35384	ERR191823_spades.fa	160	2162277	2162277	0	0	98	13514	176176	53640
ERR191824	PRJEB2999	4558994	455899400	51.4	100	100	100	100	33	35.1	200x	ERR191824_megahit.fa	160	2124629	2124629	0	0	510	13278	67541	36060	ERR191824_spades.fa	148	2157996	2157996	0	0	98	14581	152590	52700
ERR191825	PRJEB2999	5654242	565424200	51.9	100	100	100	100	33	35	248x	ERR191825_megahit.fa	167	2075207	2075207	0	0	507	12426	105281	35455	ERR191825_spades.fa	156	2105014	2105014	0	0	98	13493	208018	59787
ERR223603	PRJEB2999	3535056	353505600	52	100	100	100	100	33	34.5	155x	ERR223603_megahit.fa	198	2140258	2140258	0	0	500	10809	67540	32782	ERR223603_spades.fa	131	2156527	2156527	0	0	98	16462	108364	46883
ERR223604	PRJEB2999	4201410	420141000	52	100	100	100	100	33	34.5	184x	ERR223604_megahit.fa	168	2074677	2074677	0	0	507	12349	105280	35455	ERR223604_spades.fa	136	2098740	2098740	0	0	98	15431	172833	47438
ERR223605	PRJEB2999	3933460	393346000	51.8	100	100	100	100	33	34.5	173x	ERR223605_megahit.fa	164	2124742	2124742	0	0	500	12955	68274	36075	ERR223605_spades.fa	154	2152692	2152692	0	0	98	13978	176385	47872
ERR223606	PRJEB2999	3776980	377698000	51.8	100	100	100	100	33	34.4	166x	ERR223606_megahit.fa	163	2133325	2133325	0	0	503	13087	74544	35472	ERR223606_spades.fa	146	2159607	2159607	0	0	103	14791	191082	41975
ERR223607	PRJEB2999	4000584	400058400	51.8	100	100	100	100	33	34.6	175x	ERR223607_megahit.fa	195	2149906	2149906	0	0	500	11025	108308	35387	ERR223607_spades.fa	140	2153836	2153836	0	0	103	15384	112612	47167
ERR223608	PRJEB2999	3379858	337985800	51.7	100	100	100	100	33	34.5	148x	ERR223608_megahit.fa	166	2122729	2122729	0	0	500	12787	108312	34015	ERR223608_spades.fa	104	2131244	2131244	0	0	98	20492	110212	49838
ERR223609	PRJEB2999	3700190	370019000	51.7	100	100	100	100	33	34.4	162x	ERR223609_megahit.fa	204	2177601	2177601	0	0	500	10674	67541	30306	ERR223609_spades.fa	138	2198085	2198085	0	0	98	15928	172807	47408
ERR223610	PRJEB2999	3857734	385773400	51.8	100	100	100	100	33	34.6	169x	ERR223610_megahit.fa	166	2124170	2124170	0	0	500	12796	67543	35447	ERR223610_spades.fa	137	2151340	2151340	0	0	108	15703	152519	42310
ERR223611	PRJEB2999	3628686	362868600	51.8	100	100	100	100	33	34.6	159x	ERR223611_megahit.fa	171	2126262	2126262	0	0	500	12434	67542	35387	ERR223611_spades.fa	145	2147004	2147004	0	0	100	14806	176188	41208
ERR223612	PRJEB2999	3382680	338268000	51.8	100	100	100	100	33	34.6	148x	ERR223612_megahit.fa	159	2129047	2129047	0	0	531	13390	113677	37853	ERR223612_spades.fa	113	2142049	2142049	0	0	98	18956	172771	48803
ERR223613	PRJEB2999	3816958	381695800	51.8	100	100	100	100	33	34.5	167x	ERR223613_megahit.fa	195	2139557	2139557	0	0	500	10972	172723	30536	ERR223613_spades.fa	133	2147262	2147262	0	0	98	16144	172895	48017
ERR223614	PRJEB2999	4136110	413611000	51.9	100	100	100	100	33	34.5	181x	ERR223614_megahit.fa	170	2132664	2132664	0	0	500	12545	67543	35867	ERR223614_spades.fa	168	2160809	2160809	0	0	99	12861	172931	47725
ERR223615	PRJEB2999	4098456	409845600	51.9	100	100	100	100	33	34.6	180x	ERR223615_megahit.fa	322	2164857	2164857	0	0	500	6723	67261	18607	ERR223615_spades.fa	202	2173460	2173460	0	0	98	10759	191249	46498

ERR223616	PRJEB2999	5240324	524032400	52	100	100	100	100	33	34.5	230x	ERR223616_megahit.fa	173	2075682	2075682	0	0	507	11998	95095	35008	ERR223616_spades.fa	159	2107264	2107264	0	0	98	13253	208044	48076
ERR223619	PRJEB2999	3498488	349848800	51.6	100	100	100	100	33	34.6	153x	ERR223619_megahit.fa	166	2124403	2124403	0	0	500	12797	105284	35447	ERR223619_spades.fa	95	2130599	2130599	0	0	98	22427	140349	57554
ERR223620	PRJEB2999	3931468	393146800	51.8	100	100	100	100	33	34.5	172x	ERR223620_megahit.fa	172	2130618	2130618	0	0	500	12387	67544	35322	ERR223620_spades.fa	140	2147695	2147695	0	0	99	15340	172773	47408
ERR223621	PRJEB2999	3325616	332561600	51.8	100	100	100	100	33	34.6	146x	ERR223621_megahit.fa	164	2122796	2122796	0	0	500	12943	105281	35379	ERR223621_spades.fa	109	2131945	2131945	0	0	98	19559	172719	41975
ERR223622	PRJEB2999	2834704	283470400	51.8	100	100	100	100	33	34.7	124x	ERR223622_megahit.fa	169	2124871	2124871	0	0	503	12573	105254	31854	ERR223622_spades.fa	116	2135522	2135522	0	0	98	18409	175783	45310
ERR223623	PRJEB2999	3281226	328122600	51.8	100	100	100	100	33	34.5	144x	ERR223623_megahit.fa	163	2122559	2122559	0	0	500	13021	105279	30310	ERR223623_spades.fa	98	2131913	2131913	0	0	329	21754	207961	43964
ERR223624	PRJEB2999	3909014	390901400	52	100	100	100	100	33	34.5	171x	ERR223624_megahit.fa	208	2100744	2100744	0	0	507	10099	85634	30517	ERR223624_spades.fa	145	2100027	2100027	0	0	98	14482	127842	52713
ERR223625	PRJEB2999	4077338	407733800	51.7	100	100	100	100	33	34.6	179x	ERR223625_megahit.fa	162	2122581	2122581	0	0	500	13102	105281	34015	ERR223625_spades.fa	138	2150323	2150323	0	0	98	15582	189849	40869
ERR223626	PRJEB2999	3937098	393709800	52	100	100	100	100	33	34.7	173x	ERR223626_megahit.fa	169	2074898	2074898	0	0	507	12277	105297	35465	ERR223626_spades.fa	169	2102278	2102278	0	0	98	12439	208032	44141
ERR223627	PRJEB2999	3930962	393096200	51.7	100	100	100	100	33	34.6	172x	ERR223627_megahit.fa	163	2124556	2124556	0	0	500	13034	108309	36060	ERR223627_spades.fa	140	2147297	2147297	0	0	102	15337	207906	41208
ERR223628	PRJEB2999	6640528	664052800	51.8	100	100	100	100	33	34.5	292x	ERR223628_megahit.fa	159	2127474	2127474	0	0	541	13380	67545	37090	ERR223628_spades.fa	163	2166940	2166940	0	0	98	13294	152508	53119
ERR223629	PRJEB2999	3519586	351958600	51.8	100	100	100	100	33	34.6	154x	ERR223629_megahit.fa	202	2168615	2168615	0	0	500	10735	101846	30717	ERR223629_spades.fa	138	2197687	2197687	0	0	98	15925	125335	40675
ERR223630	PRJEB2999	4742246	474224600	51.8	100	100	100	100	33	34.6	208x	ERR223630_megahit.fa	167	2124155	2124155	0	0	500	12719	105267	30727	ERR223630_spades.fa	151	2158114	2158114	0	0	98	14292	172737	41530
ERR223631	PRJEB2999	5906566	590656600	51.8	100	100	100	100	33	34.5	259x	ERR223631_megahit.fa	159	2120681	2120681	0	0	500	13337	108292	37225	ERR223631_spades.fa	156	2155126	2155126	0	0	98	13814	110317	48776
ERR223632	PRJEB2999	4159388	415938800	51.9	100	100	100	100	33	34.5	182x	ERR223632_megahit.fa	170	2074953	2074953	0	0	507	12205	105255	35003	ERR223632_spades.fa	159	2100309	2100309	0	0	98	13209	131726	52618
ERR223633	PRJEB2999	3466274	346627400	51.8	100	100	100	100	33	34.6	152x	ERR223633_megahit.fa	166	2123284	2123284	0	0	507	12790	67543	35379	ERR223633_spades.fa	105	2133187	2133187	0	0	102	20316	198871	48793
ERR223634	PRJEB2999	3960878	396087800	51.9	100	100	100	100	33	34.5	174x	ERR223634_megahit.fa	168	2073206	2073206	0	0	507	12340	105305	34950	ERR223634_spades.fa	155	2099242	2099242	0	0	103	13543	208049	45428
ERR223635	PRJEB2999	3546958	354695800	51.7	100	100	100	100	33	34.5	156x	ERR223635_megahit.fa	167	2123509	2123509	0	0	500	12715	105286	34015	ERR223635_spades.fa	104	2132929	2132929	0	0	98	20508	173299	44099
ERR223636	PRJEB2999	4126366	412636600	51.7	100	100	100	100	33	34.6	181x	ERR223636_megahit.fa	163	2128168	2128168	0	0	500	13056	67547	35417	ERR223636_spades.fa	145	2155713	2155713	0	0	98	14866	176224	49998
ERR223637	PRJEB2999	3761596	376159600	51.7	100	100	100	100	33	34.5	165x	ERR223637_megahit.fa	166	2124059	2124059	0	0	500	12795	105302	30711	ERR223637_spades.fa	150	2152528	2152528	0	0	102	14350	152519	49505
ERR223638	PRJEB2999	3553868	355386800	51.8	100	100	100	100	33	34.5	156x	ERR223638_megahit.fa	159	2124039	2124039	0	0	502	13358	67541	35859	ERR223638_spades.fa	106	2134050	2134050	0	0	163	20132	134085	41210
ERR223639	PRJEB2999	3683108	368310800	51.9	100	100	100	100	33	34.4	161x	ERR223639_megahit.fa	163	2122668	2122668	0	0	500	13022	108309	35447	ERR223639_spades.fa	153	2154920	2154920	0	0	101	14084	152517	47382
ERR223640	PRJEB2999	3841518	384151800	51.8	100	100	100	100	33	34.5	168x	ERR223640_megahit.fa	167	2124209	2124209	0	0	513	12719	172478	30494	ERR223640_spades.fa	169	2153516	2153516	0	0	98	12742	207724	44717
ERR223641	PRJEB2999	3214706	321470600	51.8	100	100	100	100	33	34.4	141x	ERR223641_megahit.fa	166	2122339	2122339	0	0	500	12785	108309	35447	ERR223641_spades.fa	97	2132977	2132977	0	0	102	21989	172699	47408
ERR223642	PRJEB2999	3416304	341630400	52	100	100	100	100	33	34.5	150x	ERR223642_megahit.fa	176	2076478	2076478	0	0	507	11798	95095	35445	ERR223642_spades.fa	105	2086992	2086992	0	0	98	19876	108578	41602
ERR223643	PRJEB2999	2756136	275613600	51.8	100	100	100	100	33	34.5	121x	ERR223643_megahit.fa	169	2122481	2122481	0	0	500	12559	67542	30741	ERR223643_spades.fa	125	2146014	2146014	0	0	248	17168	173310	46503
ERR223644	PRJEB2999	4718758	471875800	51.9	100	100	100	100	33	34.5	207x	ERR223644_megahit.fa	166	2071159	2071159	0	0	507	12476	95095	35460	ERR223644_spades.fa	161	2102332	2102332	0	0	98	13057	208041	49032
ERR223645	PRJEB2999	3624630	362463000	51.8	100	100	100	100	33	34.5	159x	ERR223645_megahit.fa	162	2122027	2122027	0	0	500	13098	108309	37225	ERR223645_spades.fa	112	2142616	2142616	0	0	98	19130	146751	52772
ERR223646	PRJEB2999	3164208	316420800	51.7	100	100	100	100	33	34.5	139x	ERR223646_megahit.fa	197	2142418	2142418	0	0	504	10875	67545	35376	ERR223646_spades.fa	133	2152750	2152750	0	0	98	16186	189081	47109
ERR223647	PRJEB2999	3890888	389088800	51.7	100	100	100	100	33	34.5	171x	ERR223647_megahit.fa	168	2125653	2125653	0	0	500	12652	108291	36060	ERR223647_spades.fa	130	2148573	2148573	0	0	100	16527	152519	49080
ERR223648	PRJEB2999	3419682	341968200	51.9	100	100	100	100	33	34.5	150x	ERR223648_megahit.fa	151	2113516	2113516	0	0	515	13996	172562	36064	ERR223648_spades.fa	102	2123797	2123797	0	0	98	20821	173013	41601
ERR223649	PRJEB2999	3396940	339694000	51.7	100	100	100	100	33	34.6	149x	ERR223649_megahit.fa	167	2123959	2123959	0	0	500	12718	108289	36060	ERR223649_spades.fa	137	2145912	2145912	0	0	98	15663	173539	43995
ERR223650	PRJEB2999	3785506	378550600	52	100	100	100	100	33	34.4	166x	ERR223650_megahit.fa	162	2066847	2066847	0	0	537	12758	95102	35291	ERR223650_spades.fa	147	2096051	2096051	0	0	98	14258	136649	47418
ERR223651	PRJEB2999	6185818	618581800	51.8	100	100	100	100	33	34.6	272x	ERR223651_megahit.fa	163	2122872	2122872	0	0	540	13023	67544	35583	ERR223651_spades.fa	140	2155177	2155177	0	0	98	15394	152519	46645
ERR223652	PRJEB2999	5230312	523031200	51.4	100	100	100	100	33	34.5	230x	ERR223652_megahit.fa	163	2110518	2110518	0	0	522	12947	72472	34034	ERR223652_spades.fa	151	2145758	2145758	0	0	98	14210	108211	48353
ERR223653	PRJEB2999	4549756	454975600	51.8	100	100	100	100	33	34.5	200x	ERR223653_megahit.fa	166	2122796	2122796	0	0	500	12787	108302	36060	ERR223653_spades.fa	139	2152996	2152996	0	0	98	15489	207902	53509
ERR223654	PRJEB2999	3457030	345703000	51.9	100	100	100	100	33	34.5	152x	ERR223654_megahit.fa	160	2124339	2124339	0	0	502	13277	172716	35377	ERR223654_spades.fa	111	2141544	2141544	0	0	98	19293	207733	49200
ERR223655	PRJEB2999	3426566	342656600	51.7	100	100	100	100	33	34.4	150x	ERR223655_megahit.fa	162	2129524	2129524	0	0	507	13145	126149	36787	ERR223655_spades.fa	107	2137246	2137246	0	0	98	19974	152466	46895
ERR223656	PRJEB2999	4717038	471703800	50.2	100	100	100	100	33	34.7	207x	ERR223656_megahit.fa	171	2168862	2168862	0	0	528	12683	108075	30141	ERR223656_spades.fa	156	2198323	2198323	0	0	111	14091	153694	42000
ERR223657	PRJEB2999	4655272	465527200	51.9	100	100	100	100	33	34.4	204x	ERR223657_megahit.fa	166	2123808	2123808	0	0	500	12794	105288	34015	ERR223657_spades.fa	128	2150883	2150883	0	0	102	16803	218703	44150
ERR223658	PRJEB2999	4127282	412728200	51.8	100	100	100	100	33	34.4	181x	ERR223658_megahit.fa	166	2120549	2120549	0	0	500	12774	105278	35412	ERR223658_spades.fa	159	2151589	2151589	0	0	98	13532	205160	40839
ERR223659	PRJEB2999	3776398	377639800	51.8	100	100	100	100	33	34.5	166x	ERR223659_megahit.fa	159	2128256	2128256	0	0	507	13385	126246	36787	ERR223659_spades.fa	138	2152850	2152850	0	0	101	15600	172815	44540
ERR223660	PRJEB2999	3978692	397869200	51.1	100	100	100	100	33	34.5	174x	ERR223660_megahit.fa	163	2116794	2116794	0	0	523	12986	172526	31508	ERR223660_spades.fa	149	2141916	2141916	0	0	98	14375	172675	39207
ERR223661	PRJEB2999	3916826	391682600	51.7	100	100	100	100	33	34.4	172x	ERR223661_megahit.fa	170	2124649	2124649	0	0	500	12497	108343	35384	ERR223661_spades.fa	127	2148644	2148644	0	0	99	16918	176026	47364
ERR223662	PRJEB2999	3952250	395225000	52.3	100	100	100	100	33	34.5	173x	ERR223662_megahit.fa	172	2076997	2076997	0	0	507	12075	105266	35460	ERR223662_spades.fa	163	2102507	2102507	0	0	98	12898	106013	47070
ERR223663	PRJEB2999	3846422	384642200	51.9	100	100	100	100	33	34.5	169x	ERR223663_megahit.fa	168	2125036	2125036	0	0	500	12649	67541	34015	ERR223663_spades.fa	139	2152594	2152594	0	0	98	15486	82086	41430
ERR223664	PRJEB2999	8212170	821217000	51.8	100	100	100	100	33	34.5	361x	ERR223664_megahit.fa	162	2122104	2122104	0	0	500	13099	68273	35417	ERR223664_spades.fa	165	2159859	2159859	0	0	98	13090	152508	47607
ERR223665	PRJEB2999	5749440	574944000	51.8	100	100	100	100	33	34.6	252x	ERR223665_megahit.fa	167	2122905	2122905	0	0	500	12712	108315	37225	ERR223665_spades.fa	149	2155200	2155200	0	0	98	14464	191305	44382
ERR223666	PRJEB2999	3877430	387743000	51.8	100	100	100	100	33	34.5	170x	ERR223666_megahit.fa	170	2129370	2129370	0	0	500	12525	67541	35302	ERR223666_spades.fa	160	2154044	2154044	0	0	102	13462	173620	43275
ERR223667	PRJEB2999	4393684	439368400	51.7	100	100	100	100	33	34.6	193x	ERR223667_megahit.fa	163	2134968	2134968	0	0	522	13097	113807	36224	ERR223667_spades.fa	141	2154834	2154834	0	0	98	15282	153868	55245
ERR223668	PRJEB2999	8853002	885300200	51.3	100	100	100	100	33	34.5	389x	ERR223668_megahit.fa	177	2160165	2160165	0	0	509	12204	105614	26970	ERR223668_spades.fa	149	2193709	2193709	0	0	98	14722	172981	48959
ERR223669	PRJEB2999	3467604	346760400	51.7	100	100	100	100	33	34.5	152x	ERR223669_megahit.fa	170	2122443	2122443	0	0	500	12484	105264	34015	ERR223669_spades.fa	105	2134359	2134359	0	0	98	20327	188931	48627
ERR223670	PRJEB2999	3158668	315866800	51.8	100	100	100	100	33	34.5	138x	ERR223670_megahit.fa	172	2125751	2125751	0	0	524	12359	105243	30494	ERR223670_spades.fa	131	2145251	2145251	0	0	98	16375	196682	44534
ERR223671	PRJEB2999	3569300	356930000	51.7	100	100	100	100	33	34.6	156x	ERR223671_megahit.fa	171	2126685	2126685	0	0	500	12436	67545	35400	ERR223671_spades.fa	128	2150032	2150032	0	0	250	16797	207756	44034
ERR223672	PRJEB2999	3924176	392417600	51.9	100	100	100	100	33	34.4	172x	ERR223672_megahit.fa	170	2135094	2135094	0	0	500	12559	105288	35389	ERR223672_spades.fa	152	2155770	2155770	0	0	98	14182	191261	40682
ERR223673	PRJEB2999	4145084	414508400	51.7	100	100	100	100	33	34.5	182x	ERR223673_megahit.fa	158	2124966	2124966	0	0	507	13449	126148	36787	ERR223673_spades.fa	152	2149130	2149130	0	0	98	14139	189703	49399
ERR223674	PRJEB2999	4519738	451973800	51.9	100	100	100	100	33	34.6	198x	ERR223674_megahit.fa	163	2119182	2119182	0	0	535	13001	73785	31436	ERR223674_spades.fa	154	2151078	2151078	0	0	98	13968	207832	42217
ERR223675	PRJEB2999	5555552	555555200	51.7	100	100	100	100	33	34.6	244x	ERR223675_megahit.fa	158	2123560	2123560	0	0	507	13440	126148	36787	ERR223675_spades.fa	137	2151369	2151369	0	0	98	15703	152466	48792
ERR223676	PRJEB2999	7506656	750665600	51.9	100	100	100	100	33	34.6	330x	ERR223676_megahit.fa	162	2115792	2115792	0	0	537	13060	105246	30494	ERR223676_spades.fa	138	2152678	2152678	0	0	98	15599	207825	51375
ERR223677	PRJEB2999	4040154	404015400	51.8	100	100	100	100	33	34.6	177x	ERR223677_megahit.fa	159	2126768	2126768	0	0	501	13375	126147	36787	ERR223677_spades.fa	141	2153339	2153339	0	0	98	15271	207994	48402
ERR223678	PRJEB2999	3832602	383260200	52	100	100	100	100	33	34.5	168x	ERR223678_megahit.fa	248	2162763	2162763	0	0	504	8720	85711	26280	ERR223678_spades.fa	149	2109394	2109394	0	0	98	14157	108534	41129
ERR223679	PRJEB2999	4563140	456314000	51.8	100	100	100	100	33	34.5	200x	ERR223679_megahit.fa	161	2129577	2129577	0	0	504	13227	126147	36787	ERR223679_spades.fa	158	2157218	2157218	0	0	98	13653	207924	48808
ERR223680	PRJEB2999	3530036	353003600	52	100	100	100	100	33	34.6	155x	ERR223680_megahit.fa	173	2078584	2078584	0	0	507	12014	105276	35460	ERR223680_spades.fa	136	2101258	2101258	0	0	98	15450	193085	47667
ERR223681	PRJEB2999	4869612	486961200	51.7	100	100	100	100	33	34.5	214x	ERR223681_megahit.fa	159	2125887	2125887	0	0	507	13370	126149	36787	ERR223681_spades.fa	140	2150940	2150940	0	0	98	15363	152466	55246
ERR223682	PRJEB2999	4414446	441444600	51.6	100	100	100	100	33	34.5	194x	ERR223682_megahit.fa	297	2193274	2193274	0	0	502	7384	85710	23552	ERR223682_spades.fa	218	2117659	2117659	0	0	98	9714	191543	47210
ERR223683	PRJEB2999	4316190	431619000	51.7	100	100	100	100	33	34.5	189x	ERR223683_megahit.fa	173	2125826	2125826	0	0	500	12288	67545	30696	ERR223683_spades.fa	137	2162258	2162258	0	0	102	15782	152510	49421
ERR223684	PRJEB2999	4249536	424953600	51.8	100	100	100	100	33	34.5	186x	ERR223684_megahit.fa	165	2129567	2129567	0	0	500	12906	105281	35302	ERR223684_spades.fa	156	2158659	2158659	0	0	102	13837	172756	44029
ERR223685	PRJEB2999	4342462	434246200	51.7	100	100	100	100	33	34.5	190x	ERR223685_megahit.fa	167	2120625	2120625	0	0	500	12698	67542	35864	ERR223685_spades.fa	156	2149548	2149548	0	0	98	13779	208025	49119
ERR223686	PRJEB2999	4323332	432333200	51.9	100	100	100	100	33	34.6	190x	ERR223686_megahit.fa	163	2105566	2105566	0	0	509	12917	108084	34015	ERR223686_spades.fa	154	2135386	2135386	0	0	98	13866	175923	44191
ERR223687	PRJEB2999	6511894	651189400	51.7	100	100	100	100	33	34.6	286x	ERR223687_megahit.fa	165	2121181	2121181	0	0	500	12855	67544	35869	ERR223687_spades.fa	161	2153049	2153049	0	0	98	13372	152519	46687
ERR223688	PRJEB2999	11531186	1153118600	51.8	100	100	100	100	33	34.5	507x	ERR223688_megahit.fa	211	2145239	2145239	0	0	505	10167	113809	31801	ERR223688_spades.fa	155	2162196	2162196	0	0	98	13949	207991	55569
ERR223689	PRJEB2999	7430804	743080400	51.7	100	100	100	100	33	34.6	326x	ERR223689_megahit.fa	234	2194760	2194760	0	0	500	9379	66307	24288	ERR223689_spades.fa	163	2163995	2163994	1	0	98	13276	152596	46803
ERR223690	PRJEB2999	9993542	999354200	50.9	100	100	100	100	33	34.6	439x	ERR223690_megahit.fa	322	2206091	2206091	0	0	500	6851	83199	18124	ERR223690_spades.fa	183	2157575	2157575	0	0	98	11790	128244	49838
ERR223691	PRJEB2999	4508890	450889000	51.6	100	100	100	100	33	34.6	198x	ERR223691_megahit.fa	165	2124795	2124795	0	0	500	12877	67545	37225	ERR223691_spades.fa	151	2151759	2151759	0	0	98	14250	189243	49863
ERR223692	PRJEB2999	6224108	622410800	51.3	100	100	100	100	33	34.6	273x	ERR223692_megahit.fa	159	2111970	2111970	0	0	522	13282	108144	34015	ERR223692_spades.fa	146	2144880	2144880	0	0	98	14690	108297	47104
ERR223693	PRJEB2999	4349024	434902400	51.7	100	100	100	100	33	34.5	191x	ERR223693_megahit.fa	165	2125949	2125949	0	0	500	12884	67541	35867	ERR223693_spades.fa	157	2154801	2154801	0	0	98	13724	152519	41208
ERR223694	PRJEB2999	3672820	367282000	51.9	100	100	100	100	33	34.5	161x	ERR223694_megahit.fa	170	2109670	2109670	0	0	503	12409	72472	30500	ERR223694_spades.fa	149	2136385	2136385	0	0	98	14338	207656	44338
ERR223695	PRJEB2999	4070246	407024600	51.8	100	100	100	100	33	34.6	179x	ERR223695_megahit.fa	164	2121437	2121437	0	0	500	12935	67543	35370	ERR223695_spades.fa	136	2150982	2150982	0	0	98	15816	207805	49121
ERR223696	PRJEB2999	4013266	401326600	51.7	100	100	100	100	33	34.5	176x	ERR223696_megahit.fa	172	2121833	2121833	0	0	537	12336	105246	30494	ERR223696_spades.fa	155	2152103	2152103	0	0	99	13884	175951	44531
ERR223697	PRJEB2999	4847764	484776400	51.8	100	100	100	100	33	34.5	213x	ERR223697_megahit.fa	160	2125181	2125181	0	0	507	13282	126151	35911	ERR223697_spades.fa	132	2151325	2151325	0	0	98	16297	152466	48657
ERR223698	PRJEB2999	4626670	462667000	52	100	100	100	100	33	34.5	203x	ERR223698_megahit.fa	176	2077099	2077099	0	0	504	11801	95095	34801	ERR223698_spades.fa	155	2105571	2105571	0	0	98	13584	124902	53337
SRR1661153	PRJNA266539	1226610	368774527	52.8	35	300	301	301	33	30.8	162x	SRR1661153_megahit.fa	328	2039724	2039724	0	0	500	6218	66195	16888	SRR1661153_spades.fa	652	2415567	2415567	0	0	128	3704	205441	62010
SRR1661154	PRJNA266539	1109912	333669352	52.8	35	300	301	301	33	30.9	146x	SRR1661154_megahit.fa	294	2069851	2069851	0	0	500	7040	44087	17596	SRR1661154_spades.fa	417	2311349	2311349	0	0	128	5542	211156	84567
SRR1661155	PRJNA266539	1137352	341916174	52.9	35	300	301	301	33	31.1	150x	SRR1661155_megahit.fa	299	2090622	2090622	0	0	500	6992	40634	16799	SRR1661155_spades.fa	423	2320298	2320298	0	0	128	5485	207999	70223
SRR1661156	PRJNA266539	1300846	391030822	52.9	40	300	301	301	33	30.7	171x	SRR1661156_megahit.fa	373	2056611	2056611	0	0	500	5513	49120	13200	SRR1661156_spades.fa	510	2359211	2359211	0	0	128	4625	205342	63949
SRR1661157	PRJNA266539	1313690	394896413	52.8	35	300	301	301	33	31.1	173x	SRR1661157_megahit.fa	372	2067968	2067968	0	0	500	5559	53374	12437	SRR1661157_spades.fa	444	2319121	2319121	0	0	128	5223	205378	77355
SRR1661158	PRJNA266539	2392686	691305756	52.7	35	288	301	301	33	31	304x	SRR1661158_megahit.fa	1006	1945236	1945236	0	0	500	1933	22087	2802	SRR1661158_spades.fa	211	2154721	2154721	0	0	128	10211	275232	81269
SRR1661159	PRJNA266539	1513684	352287303	52	35	232	251	251	33	32.9	154x	SRR1661159_megahit.fa	358	2050194	2050194	0	0	500	5726	40834	10445	SRR1661159_spades.fa	165	2141739	2141739	0	0	128	12980	208867	65834
SRR1661160	PRJNA266539	833388	158576586	51.7	35	190	251	251	33	30	69x	SRR1661160_megahit.fa	255	2149488	2149488	0	0	502	8429	48431	19493	SRR1661160_spades.fa	563	2288956	2288955	1	0	128	4065	173535	44129
SRR1661161	PRJNA266539	1548382	444457144	52.4	35	287	301	301	33	30.7	195x	SRR1661161_megahit.fa	471	2130106	2130106	0	0	500	4522	37548	8986	SRR1661161_spades.fa	212	2217461	2217461	0	0	128	10459	208068	75123
SRR1661162	PRJNA266539	2762432	444081843	51.8	35	160	251	251	33	28.6	195x	SRR1661162_megahit.fa	308	2094832	2094832	0	0	502	6801	48088	12895	SRR1661162_spades.fa	168	2170560	2170560	0	0	128	12920	153107	55311
SRR1661163	PRJNA266539	1483546	426554164	52.3	35	287	301	301	33	31.3	187x	SRR1661163_megahit.fa	498	2059209	2059209	0	0	500	4134	30752	6995	SRR1661163_spades.fa	144	2122119	2122119	0	0	128	14736	207307	75624
SRR1661164	PRJNA266539	1486928	431654887	52.6	35	290	301	301	33	31.1	189x	SRR1661164_megahit.fa	588	2063487	2063487	0	0	500	3509	24387	6095	SRR1661164_spades.fa	146	2132449	2132449	0	0	128	14605	323833	89669
SRR1661165	PRJNA266539	1513826	437455311	52.4	35	288	301	301	33	30.5	192x	SRR1661165_megahit.fa	432	2090019	2090019	0	0	500	4838	41772	10861	SRR1661165_spades.fa	205	2192226	2192226	0	0	128	10693	207744	67730
SRR1661166	PRJNA266539	1385982	400285304	52.5	35	288	301	301	33	31.1	176x	SRR1661166_megahit.fa	422	2109524	2109524	0	0	500	4998	31836	8602	SRR1661166_spades.fa	161	2191212	2191212	0	0	128	13610	208068	79047
SRR1661167	PRJNA266539	2223950	632713816	52.7	35	284	301	301	33	31.5	278x	SRR1661167_megahit.fa	788	2054057	2054057	0	0	500	2606	30959	4212	SRR1661167_spades.fa	180	2195392	2195392	0	0	128	12196	211204	85067
SRR1661168	PRJNA266539	819172	155488997	51.5	35	189	251	251	33	31.1	68x	SRR1661168_megahit.fa	232	2059225	2059225	0	0	508	8875	52953	18332	SRR1661168_spades.fa	770	2216109	2216102	7	0	128	2878	105727	48154
SRR1661169	PRJNA266539	1489104	247685673	51.8	35	166	251	251	33	28.6	108x	SRR1661169_megahit.fa	195	2096382	2096382	0	0	502	10750	64409	20470	SRR1661169_spades.fa	147	2151549	2151548	1	0	128	14636	173037	47711
SRR1661170	PRJNA266539	314296	66030668	51.2	35	210	251	251	33	32.5	29x	SRR1661170_megahit.fa	482	2119945	2119945	0	0	500	4398	33129	7438	SRR1661170_spades.fa	342	2186866	2186865	1	0	128	6394	89355	25442
SRR1661171	PRJNA266539	1191162	358133900	52.5	51	300	301	301	33	33.5	157x	SRR1661171_megahit.fa	212	2098179	2098179	0	0	503	9897	109056	22798	SRR1661171_spades.fa	171	2196796	2196796	0	0	128	12846	246028	61255
SRR1661172	PRJNA266539	1370862	412138972	52.4	37	300	301	301	33	33.5	181x	SRR1661172_megahit.fa	241	2138250	2138250	0	0	502	8872	84715	19440	SRR1661172_spades.fa	180	2242292	2242292	0	0	128	12457	210896	65861
SRR1661173	PRJNA266539	1248094	312534581	52.7	35	250	251	251	33	32.7	137x	SRR1661173_megahit.fa	344	2113185	2113185	0	0	502	6142	61529	13195	SRR1661173_spades.fa	138	2173628	2173628	0	0	128	15750	207887	60621
SRR1661174	PRJNA266539	1852892	352258735	52.4	35	190	251	251	33	34.2	154x	SRR1661174_megahit.fa	363	2095266	2095266	0	0	506	5772	40517	10717	SRR1661174_spades.fa	192	2184575	2184575	0	0	128	11377	207709	48234
SRR1661175	PRJNA266539	1783268	444570887	52.3	35	249	251	251	33	33.1	195x	SRR1661175_megahit.fa	367	2104121	2104121	0	0	500	5733	35068	12404	SRR1661175_spades.fa	164	2181517	2181517	0	0	128	13301	207849	60622
SRR1661176	PRJNA266539	1361908	340664412	51.7	35	250	251	251	33	32.4	149x	SRR1661176_megahit.fa	303	2127856	2127856	0	0	500	7022	52985	16800	SRR1661176_spades.fa	158	2180551	2180551	0	0	128	13800	207897	67224
SRR1661177	PRJNA266539	1247046	214088898	52.1	35	171	251	251	33	29.7	94x	SRR1661177_megahit.fa	206	2110553	2110553	0	0	501	10245	64816	22483	SRR1661177_spades.fa	146	2160834	2160833	1	0	128	14800	154326	53146

SRR1661178	PRJNA266539	797070	140420370	52.2	35	176	251	251	33	29.1	61x	SRR1661178_megahit.fa	186	2110965	2110965	0	0	511	11349	103677	23595	SRR1661178_spades.fa	150	2147588	2147588	0	0	128	14317	176681	44269
SRR1661179	PRJNA266539	1266914	364568754	51.3	35	287	301	301	33	31.1	160x	SRR1661179_megahit.fa	341	2157901	2157901	0	0	501	6328	55585	13001	SRR1661179_spades.fa	144	2241611	2241611	0	0	128	15566	336436	138565
SRR1661180	PRJNA266539	719520	216327960	52.3	39	300	301	301	33	33.8	95x	SRR1661180_megahit.fa	180	2086967	2086967	0	0	501	11594	64364	22902	SRR1661180_spades.fa	119	2153046	2153046	0	0	128	18092	207701	72656
SRR1661181	PRJNA266539	1380306	401746527	52.7	35	291	301	301	33	30.8	176x	SRR1661181_megahit.fa	338	2081993	2081993	0	0	500	6159	35798	13047	SRR1661181_spades.fa	178	2167994	2167994	0	0	128	12179	204949	66351
SRR1661182	PRJNA266539	1854138	548590442	52.7	35	295	301	301	33	30.1	241x	SRR1661182_megahit.fa	666	2089033	2089033	0	0	500	3136	29742	6093	SRR1661182_spades.fa	251	2201130	2201130	0	0	128	8769	204949	64022
SRR1661183	PRJNA266539	1787416	344291721	51.7	35	192	251	251	33	34	151x	SRR1661183_megahit.fa	274	2100608	2100608	0	0	500	7666	46816	15367	SRR1661183_spades.fa	183	2171505	2171503	2	0	128	11866	207699	49336
SRR1661184	PRJNA266539	1590284	306418123	52.4	35	192	251	251	33	33.9	134x	SRR1661184_megahit.fa	315	2098965	2098965	0	0	509	6663	46467	13710	SRR1661184_spades.fa	153	2176800	2176800	0	0	128	14227	207917	67358
SRR1661185	PRJNA266539	1992628	586424189	52.6	35	294	301	301	33	30.4	257x	SRR1661185_megahit.fa	732	2083311	2083311	0	0	500	2846	21455	5240	SRR1661185_spades.fa	190	2214085	2214085	0	0	128	11653	246443	95325
SRR1661186	PRJNA266539	1562330	305527795	51.4	35	195	251	251	33	33.9	134x	SRR1661186_megahit.fa	275	2111558	2111558	0	0	509	7678	40901	15071	SRR1661186_spades.fa	183	2174763	2174763	0	0	128	11883	108459	57895
SRR1661187	PRJNA266539	1163626	290763364	53.4	35	249	251	251	33	30.6	127x	SRR1661187_megahit.fa	407	2089886	2089886	0	0	500	5134	40138	12441	SRR1661187_spades.fa	193	2200398	2200398	0	0	128	11401	208019	67425
SRR1661188	PRJNA266539	1512422	293730915	51.4	35	194	251	251	33	34.2	129x	SRR1661188_megahit.fa	224	2110621	2110621	0	0	503	9422	88303	19770	SRR1661188_spades.fa	176	2173004	2173004	0	0	128	12346	207913	48275
SRR1661189	PRJNA266539	829254	162608975	51.6	35	196	251	251	33	33.7	71x	SRR1661189_megahit.fa	227	2114600	2114600	0	0	505	9315	49907	21251	SRR1661189_spades.fa	205	2176982	2176982	0	0	128	10619	113167	59012
SRR1661190	PRJNA266539	4345502	1298632307	52.6	35	298	301	301	33	30.4	571x	SRR1661190_megahit.fa	1124	697964	697964	0	0	500	620	2024	574	SRR1661190_spades.fa	393	2300624	2300624	0	0	128	5854	207925	76623
SRR1661191	PRJNA266539	492786	148086058	52.3	35	300	301	301	33	30.9	65x	SRR1661191_megahit.fa	185	2111747	2111747	0	0	502	11414	65948	30212	SRR1661191_spades.fa	118	2168261	2168261	0	0	128	18375	207719	70450
SRR1661192	PRJNA266539	858334	257978769	52.5	35	300	301	301	33	30.5	113x	SRR1661192_megahit.fa	211	2103316	2103316	0	0	500	9968	49786	21203	SRR1661192_spades.fa	125	2181527	2181527	0	0	128	17452	207903	90629
SRR1661193	PRJNA266539	676888	203434426	52.5	35	300	301	301	33	30.8	89x	SRR1661193_megahit.fa	188	2108657	2108657	0	0	503	11216	103336	26947	SRR1661193_spades.fa	105	2174715	2174715	0	0	128	20711	207726	85991
SRR1661194	PRJNA266539	1025154	308168634	52.5	35	300	301	301	33	30.5	135x	SRR1661194_megahit.fa	209	2091973	2091973	0	0	501	10009	67552	23221	SRR1661194_spades.fa	167	2174224	2174224	0	0	128	13019	207744	68228
SRR1661195	PRJNA266539	938520	163446659	52	35	174	251	251	33	31	71x	SRR1661195_megahit.fa	221	2050808	2050808	0	0	504	9279	58022	18621	SRR1661195_spades.fa	162	2108211	2108211	0	0	128	13013	205094	47772
SRR1661196	PRJNA266539	710102	127866250	51.7	35	180	251	251	33	30.6	56x	SRR1661196_megahit.fa	226	2112944	2112944	0	0	530	9349	67545	19822	SRR1661196_spades.fa	160	2156302	2156302	0	0	128	13476	208058	49666
SRR1661197	PRJNA266539	1445810	361824531	52.2	35	250	251	251	33	33.5	159x	SRR1661197_megahit.fa	349	2101279	2101279	0	0	503	6020	65940	13143	SRR1661197_spades.fa	159	2179358	2179358	0	0	128	13706	207862	67448
SRR1661198	PRJNA266539	1228600	266640138	52.3	35	217	251	251	33	35.1	117x	SRR1661198_megahit.fa	263	2109666	2109666	0	0	501	8021	55928	17774	SRR1661198_spades.fa	1162	2369344	2369344	0	0	128	2039	207862	46850
SRR1661199	PRJNA266539	2341156	546155164	52.3	35	233	251	251	33	32.8	240x	SRR1661199_megahit.fa	515	2077386	2077386	0	0	502	4033	26271	6386	SRR1661199_spades.fa	349	2262009	2262009	0	0	128	6481	207917	63624
SRR1661200	PRJNA266539	1666434	416983752	52	35	250	251	251	33	33.6	183x	SRR1661200_megahit.fa	396	2093080	2093080	0	0	503	5285	43990	10326	SRR1661200_spades.fa	150	2178075	2178075	0	0	128	14520	208088	71057
SRR1661201	PRJNA266539	1779962	439361098	53.9	35	246	251	251	33	31.4	193x	SRR1661201_megahit.fa	976	1878792	1878792	0	0	500	1924	37548	3082	SRR1661201_spades.fa	175	2193365	2193365	0	0	128	12533	207831	67451
SRR1661202	PRJNA266539	1548706	383904547	54.2	35	247	251	251	33	31.3	168x	SRR1661202_megahit.fa	815	1933444	1933444	0	0	500	2372	37543	4023	SRR1661202_spades.fa	176	2195349	2195349	0	0	128	12473	207965	61403
SRR1661203	PRJNA266539	1631724	402574663	54.3	35	246	251	251	33	30.7	177x	SRR1661203_megahit.fa	905	1950939	1950939	0	0	500	2155	24111	3702	SRR1661203_spades.fa	207	2202486	2202486	0	0	128	10640	207871	67398
SRR1661204	PRJNA266539	1145360	265353278	53.7	35	231	251	251	33	33.4	116x	SRR1661204_megahit.fa	303	2096624	2096624	0	0	504	6919	44260	14401	SRR1661204_spades.fa	192	2173352	2173352	0	0	128	11319	223239	78149
SRR1661205	PRJNA266539	1684328	315088078	51.7	35	187	251	251	33	34.2	138x	SRR1661205_megahit.fa	411	2094242	2094242	0	0	505	5095	37535	10202	SRR1661205_spades.fa	198	2178925	2178925	0	0	128	11004	207975	49599
SRR1661206	PRJNA266539	926396	229184402	54.8	35	247	251	251	33	30.1	100x	SRR1661206_megahit.fa	744	1997665	1997665	0	0	500	2685	24313	5097	SRR1661206_spades.fa	184	2192957	2192957	0	0	128	11918	207859	61184
SRR1661207	PRJNA266539	2297762	367434949	49.4	35	159	251	251	33	29.1	161x	SRR1661207_megahit.fa	253	2150870	2150870	0	0	500	8501	63005	18247	SRR1661207_spades.fa	170	2222528	2222528	0	0	128	13073	173001	52304
SRR1661208	PRJNA266539	564144	132576279	51.9	35	235	251	251	33	33.7	58x	SRR1661208_megahit.fa	199	2117154	2117154	0	0	500	10638	67545	21100	SRR1661208_spades.fa	157	2170186	2170186	0	0	133	13822	210834	49689
SRR1661209	PRJNA266539	1174966	199141547	52.3	35	169	251	251	33	29.9	87x	SRR1661209_megahit.fa	191	2108234	2108234	0	0	510	11037	105266	23340	SRR1661209_spades.fa	146	2160530	2160530	0	0	128	14798	152658	47537
SRR1661210	PRJNA266539	1833796	443501581	52.2	35	241	251	251	33	32.8	195x	SRR1661210_megahit.fa	472	2079889	2079889	0	0	500	4406	29993	7170	SRR1661210_spades.fa	147	2178222	2178222	0	0	128	14817	207820	70869
SRR1661211	PRJNA266539	1700256	326200438	51.9	35	191	251	251	33	34	143x	SRR1661211_megahit.fa	327	2042610	2042610	0	0	502	6246	42126	12168	SRR1661211_spades.fa	182	2124265	2124265	0	0	128	11671	208090	59755
SRR1661212	PRJNA266539	1028798	238135808	51.4	35	231	251	251	33	31.9	104x	SRR1661212_megahit.fa	243	2112988	2112988	0	0	500	8695	57072	20410	SRR1661212_spades.fa	183	2178680	2178680	0	0	128	11905	207833	71271
SRR1661213	PRJNA266539	1434884	357245153	51.7	35	248	251	251	33	33.4	157x	SRR1661213_megahit.fa	295	2110153	2110153	0	0	510	7153	53789	16622	SRR1661213_spades.fa	169	2176556	2176556	0	0	128	12879	293533	55697
SRR1661214	PRJNA266539	1476564	364983174	54.4	35	247	251	251	33	31.3	160x	SRR1661214_megahit.fa	1020	1851299	1851299	0	0	500	1814	27139	2800	SRR1661214_spades.fa	157	2185041	2185041	0	0	128	13917	207848	72670
SRR1661215	PRJNA266539	1372638	338743957	53.4	35	246	251	251	33	31.8	148x	SRR1661215_megahit.fa	353	2024326	2024326	0	0	501	5734	45480	13306	SRR1661215_spades.fa	152	2178237	2178237	0	0	128	14330	207874	65500
SRR1661216	PRJNA266539	410288	78975808	52.3	35	192	251	251	33	29.4	34x	SRR1661216_megahit.fa	287	2118201	2118201	0	0	500	7380	49409	14147	SRR1661216_spades.fa	160	2165006	2165006	0	0	128	13531	97050	47334
SRR1661217	PRJNA266539	1420390	294567416	52	35	207	251	251	33	32.7	129x	SRR1661217_megahit.fa	242	2113489	2113489	0	0	500	8733	49028	18148	SRR1661217_spades.fa	232	2183629	2183627	2	0	128	9412	261281	60617
SRR1661218	PRJNA266539	2531910	415813847	51.8	35	164	251	251	33	29.6	182x	SRR1661218_megahit.fa	280	2049048	2049048	0	0	501	7318	60695	14880	SRR1661218_spades.fa	148	2116941	2116941	0	0	128	14303	208084	59922
SRR1661219	PRJNA266539	545184	102280686	52.6	35	187	251	251	33	28.5	44x	SRR1661219_megahit.fa	237	2147362	2147362	0	0	508	9060	54916	18259	SRR1661219_spades.fa	170	2195613	2195612	1	0	128	12915	172860	40787
SRR1661220	PRJNA266539	1178840	244384253	52.3	35	207	251	251	33	32	107x	SRR1661220_megahit.fa	230	2103630	2103630	0	0	509	9146	67162	19624	SRR1661220_spades.fa	227	2182422	2182422	0	0	128	9614	207953	53376
SRR1661221	PRJNA266539	450022	89490522	51.7	35	198	251	251	33	29.9	39x	SRR1661221_megahit.fa	272	2148485	2148485	0	0	505	7898	60205	18760	SRR1661221_spades.fa	377	2275208	2275205	3	0	129	6035	172974	44162
SRR1661222	PRJNA266539	692226	132463479	51.5	35	191	251	251	33	30.3	58x	SRR1661222_megahit.fa	244	2110223	2110223	0	0	505	8648	63485	16974	SRR1661222_spades.fa	269	2181577	2181576	1	0	128	8109	210787	52003
SRR1661223	PRJNA266539	741194	130055145	51.7	35	175	251	251	33	29.5	57x	SRR1661223_megahit.fa	225	2069629	2069629	0	0	507	9198	63487	19632	SRR1661223_spades.fa	255	2151116	2151114	2	0	128	8435	176373	47163
SRR1661224	PRJNA266539	1842214	303868150	52	35	164	251	251	33	29.8	133x	SRR1661224_megahit.fa	270	2134986	2134986	0	0	502	7907	50524	15798	SRR1661224_spades.fa	171	2209038	2209038	0	0	128	12918	207664	53117
SRR1661225	PRJNA266539	1577772	376514428	52.3	35	238	251	251	33	31.8	165x	SRR1661225_megahit.fa	403	2109022	2109022	0	0	502	5233	34569	10248	SRR1661225_spades.fa	148	2217801	2217800	1	0	128	14985	207847	81671
SRR1661226	PRJNA266539	1735780	338723770	51.6	35	195	251	251	33	33.9	148x	SRR1661226_megahit.fa	282	2108710	2108710	0	0	508	7477	49055	15446	SRR1661226_spades.fa	208	2192576	2192576	0	0	128	10541	208038	63204
SRR1661227	PRJNA266539	772466	147290052	52.3	35	190	251	251	33	29.2	64x	SRR1661227_megahit.fa	204	2113965	2113965	0	0	525	10362	105238	20838	SRR1661227_spades.fa	157	2170455	2170453	2	0	128	13824	175976	44428
SRR1661228	PRJNA266539	1970496	377250481	51.8	35	191	251	251	33	33.7	165x	SRR1661228_megahit.fa	478	2092880	2092880	0	0	500	4378	47686	7653	SRR1661228_spades.fa	154	2174294	2174294	0	0	128	14118	207822	67963
SRR1661229	PRJNA266539	361076	71134283	51.5	35	197	251	251	33	29.2	31x	SRR1661229_megahit.fa	429	2078223	2078223	0	0	507	4844	30053	7607	SRR1661229_spades.fa	135	2080843	2080843	0	0	128	15413	88042	43386
SRR1661230	PRJNA266539	1144472	280454693	52.7	35	245	251	251	33	32.9	123x	SRR1661230_megahit.fa	713	2065867	2065867	0	0	500	2897	25388	5193	SRR1661230_spades.fa	193	2178467	2178467	0	0	128	11287	152578	57860
SRR1661231	PRJNA266539	1354922	330799380	52.7	35	244	251	251	33	33.1	145x	SRR1661231_megahit.fa	804	2049034	2049034	0	0	502	2548	18990	4514	SRR1661231_spades.fa	196	2183845	2183845	0	0	128	11142	152579	56254
SRR1661232	PRJNA266539	821506	146662217	51.7	35	178	251	251	33	29.9	64x	SRR1661232_megahit.fa	235	2111111	2111111	0	0	502	8983	63494	18540	SRR1661232_spades.fa	193	2168938	2168935	3	0	128	11238	172909	53609
SRR1661233	PRJNA266539	1010898	190505922	51.1	35	188	251	251	33	29.8	83x	SRR1661233_megahit.fa	257	2106439	2106439	0	0	505	8196	60275	16912	SRR1661233_spades.fa	182	2172590	2172590	0	0	128	11937	208043	53000
SRR1661234	PRJNA266539	944220	283884604	52.5	47	300	301	301	33	33.5	124x	SRR1661234_megahit.fa	180	2104783	2104783	0	0	500	11693	72577	27655	SRR1661234_spades.fa	123	2169776	2169776	0	0	128	17640	292770	80283
SRR1661235	PRJNA266539	1400134	420947063	52.5	53	300	301	301	33	33.4	185x	SRR1661235_megahit.fa	237	2091031	2091031	0	0	500	8822	72577	17029	SRR1661235_spades.fa	172	2192990	2192990	0	0	128	12749	249914	80285
SRR1661236	PRJNA266539	1326770	398902506	52.6	64	300	301	301	33	33.6	175x	SRR1661236_megahit.fa	222	2098045	2098045	0	0	502	9450	73029	23037	SRR1661236_spades.fa	138	2178628	2178628	0	0	128	15787	246033	84418
SRR1661237	PRJNA266539	1435226	431498293	52.2	52	300	301	301	33	33.7	189x	SRR1661237_megahit.fa	219	2092922	2092922	0	0	510	9556	72551	19373	SRR1661237_spades.fa	138	2177907	2177907	0	0	128	15781	311816	80144
SRR1661238	PRJNA266539	1485242	446538721	52.3	63	300	301	301	33	33.4	196x	SRR1661238_megahit.fa	234	2096804	2096804	0	0	502	8960	43949	19243	SRR1661238_spades.fa	145	2179604	2179604	0	0	128	15031	253248	85228
SRR1661239	PRJNA266539	1451738	436464637	52.4	37	300	301	301	33	33.8	191x	SRR1661239_megahit.fa	220	2088866	2088866	0	0	503	9494	63359	20158	SRR1661239_spades.fa	158	2184694	2184694	0	0	128	13827	271954	72289
SRR1661240	PRJNA266539	1333916	401046101	52.4	35	300	301	301	33	33.7	176x	SRR1661240_megahit.fa	225	2098307	2098307	0	0	505	9325	67977	20298	SRR1661240_spades.fa	138	2183250	2183250	0	0	128	15820	352850	85228
SRR1661241	PRJNA266539	1088372	327218868	52.5	65	300	301	301	33	33.6	143x	SRR1661241_megahit.fa	217	2101970	2101970	0	0	500	9686	72513	20530	SRR1661241_spades.fa	141	2178300	2178300	0	0	128	15448	249978	72289
SRR1661242	PRJNA266539	1196374	291040051	52.7	35	243	251	251	33	33	128x	SRR1661242_megahit.fa	456	2094237	2094237	0	0	500	4592	39047	9316	SRR1661242_spades.fa	186	2179881	2179881	0	0	128	11719	207888	60154
SRR1661243	PRJNA266539	1522232	297882575	51.5	35	195	251	251	33	34	131x	SRR1661243_megahit.fa	291	2102600	2102600	0	0	511	7225	59939	15131	SRR1661243_spades.fa	166	2174336	2174335	1	0	128	13098	208048	55769
SRR1661244	PRJNA266539	968078	200936425	51.1	35	207	251	251	33	34.3	88x	SRR1661244_megahit.fa	257	2118887	2118887	0	0	500	8244	67545	18149	SRR1661244_spades.fa	1088	2373205	2373205	0	0	128	2181	172859	44465
SRR1661245	PRJNA266539	675726	140849031	51.8	35	208	251	251	33	34	61x	SRR1661245_megahit.fa	290	2111251	2111251	0	0	500	7280	63571	15078	SRR1661245_spades.fa	308	2195147	2195145	2	0	128	7127	207960	44385
SRR1661246	PRJNA266539	2258576	504496674	51.3	35	223	251	251	33	33.5	221x	SRR1661246_megahit.fa	381	2110447	2110447	0	0	502	5539	47284	12718	SRR1661246_spades.fa	221	2192720	2192720	0	0	128	9921	207878	67351
SRR1661247	PRJNA266539	1375308	282963888	51.3	35	205	251	251	33	34.1	124x	SRR1661247_megahit.fa	253	2110321	2110321	0	0	500	8341	87923	17721	SRR1661247_spades.fa	1986	2569666	2569659	7	0	128	1293	223511	36981
SRR1661248	PRJNA266539	1136614	232937672	52.2	35	204	251	251	33	33.9	102x	SRR1661248_megahit.fa	307	2103741	2103741	0	0	500	6852	48092	14477	SRR1661248_spades.fa	207	2185543	2185543	0	0	128	10558	207912	57716
SRR1661249	PRJNA266539	1849064	375206873	51.7	35	202	251	251	33	34.1	165x	SRR1661249_megahit.fa	324	2107677	2107677	0	0	500	6505	41772	13886	SRR1661249_spades.fa	272	2195559	2195557	2	0	128	8071	207898	50192
SRR1661250	PRJNA266539	2112508	411928749	53.8	35	194	251	251	33	34.2	181x	SRR1661250_megahit.fa	562	2078619	2078619	0	0	501	3698	34928	6968	SRR1661250_spades.fa	173	2167189	2167189	0	0	128	12527	176021	60195
SRR1661251	PRJNA266539	2197746	506708797	51.6	35	230	251	251	33	33.5	222x	SRR1661251_megahit.fa	525	2098283	2098283	0	0	501	3996	32213	6874	SRR1661251_spades.fa	214	2194194	2194194	0	0	128	10253	207870	64263
SRR1661252	PRJNA266539	941236	165043249	52	35	175	251	251	33	30.7	72x	SRR1661252_megahit.fa	228	2114110	2114110	0	0	507	9272	66366	19759	SRR1661252_spades.fa	221	2174693	2174690	3	0	128	9840	207888	46800
SRR1661253	PRJNA266539	878746	182758782	52.1	35	207	251	251	33	33.8	80x	SRR1661253_megahit.fa	282	2110277	2110277	0	0	507	7483	100439	16038	SRR1661253_spades.fa	359	2205603	2205603	0	0	128	6143	207840	55698
SRR1661254	PRJNA266539	1146434	236118838	51.9	35	205	251	251	33	33.7	103x	SRR1661254_megahit.fa	309	2108546	2108546	0	0	504	6823	56580	14987	SRR1661254_spades.fa	215	2188377	2188377	0	0	128	10178	207759	64461
SRR1661255	PRJNA266539	1008620	173610226	51.7	35	172	251	251	33	28.8	76x	SRR1661255_megahit.fa	194	2106237	2106237	0	0	502	10856	72682	22399	SRR1661255_spades.fa	1626	2454540	2454536	4	0	128	1509	172935	40047
SRR1661256	PRJNA266539	1118884	336394899	52.5	59	300	301	301	33	33.8	147x	SRR1661256_megahit.fa	194	2099138	2099138	0	0	500	10820	73263	23052	SRR1661256_spades.fa	130	2173737	2173737	0	0	128	16721	207961	80876
SRR1661257	PRJNA266539	674826	132311086	52.3	35	196	251	251	33	29.1	58x	SRR1661257_megahit.fa	204	2113124	2113124	0	0	502	10358	94925	23251	SRR1661257_spades.fa	402	2204507	2204464	43	0	128	5483	172751	41573
SRR1661258	PRJNA266539	2154892	399575947	52.9	35	185	251	251	33	33.3	175x	SRR1661258_megahit.fa	479	2074734	2074734	0	0	500	4331	43046	8175	SRR1661258_spades.fa	171	2181414	2181414	0	0	128	12756	208082	56419
SRR1661259	PRJNA266539	1115472	278493794	51.8	35	249	251	251	33	32.3	122x	SRR1661259_megahit.fa	276	2111670	2111670	0	0	500	7650	61858	16639	SRR1661259_spades.fa	179	2184047	2184047	0	0	128	12201	207923	52996
SRR1661260	PRJNA266539	1174012	352970893	52.7	74	300	301	301	33	33.4	155x	SRR1661260_megahit.fa	204	2033521	2033521	0	0	502	9968	58878	21872	SRR1661260_spades.fa	197	2138668	2138668	0	0	128	10856	207658	66632
SRR1661261	PRJNA266539	3484782	803570577	52.5	35	230	301	301	33	33.3	353x	SRR1661261_megahit.fa	892	1942327	1942327	0	0	500	2177	19659	3580	SRR1661261_spades.fa	174	2145290	2145290	0	0	128	12329	208078	84889
SRR1661262	PRJNA266539	1771072	340975133	51.5	35	192	251	251	33	33.4	149x	SRR1661262_megahit.fa	331	2088739	2088739	0	0	500	6310	57214	11692	SRR1661262_spades.fa	169	2175673	2175672	1	0	128	12873	208074	52981
SRR1661263	PRJNA266539	1170338	351871408	52.5	48	300	301	301	33	33.5	154x	SRR1661263_megahit.fa	207	2104049	2104049	0	0	500	10164	93085	23031	SRR1661263_spades.fa	132	2176185	2176185	0	0	128	16486	214913	80834
SRR1661264	PRJNA266539	360296	108322342	52.3	35	300	301	301	33	33.3	47x	SRR1661264_megahit.fa	197	2112350	2112350	0	0	501	10722	119627	23076	SRR1661264_spades.fa	111	2157586	2157586	0	0	128	19437	211914	63385
SRR1661265	PRJNA266539	2091450	400746453	52.1	35	191	251	251	33	33.4	176x	SRR1661265_megahit.fa	369	2095281	2095281	0	0	502	5678	46770	10025	SRR1661265_spades.fa	152	2174734	2174734	0	0	128	14307	207999	55133
SRR1661266	PRJNA266539	1158154	348208580	52.4	63	300	301	301	33	33.6	153x	SRR1661266_megahit.fa	219	2062509	2062509	0	0	502	9417	72884	21550	SRR1661266_spades.fa	147	2162363	2162363	0	0	128	14709	238343	77790
SRR1661267	PRJNA266539	891954	268159758	52.1	70	300	301	301	33	33.5	117x	SRR1661267_megahit.fa	213	2086360	2086360	0	0	506	9795	66344	23197	SRR1661267_spades.fa	377	2298082	2298082	0	0	128	6095	207698	72815
SRR1661268	PRJNA266539	1077306	213238988	51.4	35	197	251	251	33	33.8	93x	SRR1661268_megahit.fa	275	2108202	2108202	0	0	500	7666	48914	16064	SRR1661268_spades.fa	214	2181711	2181711	0	0	128	10194	207942	47742
SRR1661269	PRJNA266539	1016962	208805390	51.3	35	205	251	251	33	33	91x	SRR1661269_megahit.fa	306	2106734	2106734	0	0	500	6884	50628	13353	SRR1661269_spades.fa	183	2174688	2174688	0	0	128	11883	207955	55739
SRR1661270	PRJNA266539	1660148	395645674	52.6	35	238	251	251	33	33.6	174x	SRR1661270_megahit.fa	474	2082852	2082852	0	0	500	4394	34625	7553	SRR1661270_spades.fa	155	2173295	2173295	0	0	128	14021	207793	63715
SRR1661271	PRJNA266539	1627344	386341309	52.6	35	237	251	251	33	33	169x	SRR1661271_megahit.fa	439	2081020	2081020	0	0	501	4740	49020	8181	SRR1661271_spades.fa	167	2184298	2184298	0	0	128	13079	269364	64801
SRR1661272	PRJNA266539	2719508	647426683	52.9	35	238	251	251	33	33.5	284x	SRR1661272_megahit.fa	1165	1812738	1812738	0	0	501	1555	22820	1988	SRR1661272_spades.fa	151	2178410	2178410	0	0	128	14426	207954	84717
SRR1661273	PRJNA266539	2069780	490193037	52.7	35	236	251	251	33	33.5	215x	SRR1661273_megahit.fa	479	2062022	2062022	0	0	500	4304	49218	7928	SRR1661273_spades.fa	208	2174733	2174733	0	0	128	10455	207830	76001
SRR1661274	PRJNA266539	1844954	446847615	53.2	35	242	251	251	33	33	196x	SRR1661274_megahit.fa	747	2019182	2019182	0	0	500	2703	35176	4292	SRR1661274_spades.fa	162	2178728	2178728	0	0	128	13448	207966	94555
SRR1661275	PRJNA266539	1437778	432245833	52.3	57	300	301	301	33	34.1	190x	SRR1661275_megahit.fa	245	2140589	2140589	0	0	504	8737	66276	18690	SRR1661275_spades.fa	157	2232841	2232841	0	0	128	14221	277207	76375
SRR1661276	PRJNA266539	753900	131708736	51.5	35	174	251	251	33	29.4	57x	SRR1661276_megahit.fa	223	2111720	2111720	0	0	506	9469	64284	18538	SRR1661276_spades.fa	219	2176038	2176035	3	0	128	9936	208091	41164
SRR1661277	PRJNA266539	671436	125778326	51.7	35	187	251	251	33	29.9	55x	SRR1661277_megahit.fa	241	2115878	2115878	0	0	506	8779	63793	19488	SRR1661277_spades.fa	271	2188927	2188923	4	0	128	8077	207948	44150
SRR1661278	PRJNA266539	575922	105502549	52	35	183	251	251	33	30.4	46x	SRR1661278_megahit.fa	243	2122570	2122570	0	0	500	8734	63508	18149	SRR1661278_spades.fa	140	2164731	2164730	1	0	128	15462	211176	48034
SRR1661279	PRJNA266539	649250	114101435	52.4	35	175	251	251	33	30.8	50x	SRR1661279_megahit.fa	220	2116996	2116996	0	0	514	9622	82069	19522	SRR1661279_spades.fa	207	2174025	2174025	0	0	128	10502	208039	47748
SRR1661280	PRJNA266539	1517302	374061643	52.9	35	246	251	251	33	31.6	164x	SRR1661280_megahit.fa	748	2037933	2037933	0	0	502	2724	34514	4338	SRR1661280_spades.fa	156	2188529	2188529	0	0	128	14029	216090	68219
SRR1661281	PRJNA266539	1547646	381411342	53.2	35	246	251	251	33	31.8	167x	SRR1661281_megahit.fa	647	1998611	1998611	0	0	500	3089	27822	5199	SRR1661281_spades.fa	180	2139877	2139877	0	0	128	11888	207925	67272
SRR1661282	PRJNA266539	1479810	444875441	52.3	52	300	301	301	33	33.4	195x	SRR1661282_megahit.fa	241	2093159	2093159	0	0	500	8685	92979	19139	SRR1661282_spades.fa	201	2210969	2210969	0	0	128	10999	208034	60357
SRR1661283	PRJNA266539	1279476	315059355	52.9	35	246	251	251	33	31.4	138x	SRR1661283_megahit.fa	537	2087285	2087285	0	0	500	3886	32455	7282	SRR1661283_spades.fa	225	2190619	2190619	0	0	128	9736	207868	57665

SRR1661284	PRJNA266539	712520	131716531	53.5	35	184	251	251	33	29.7	57x	SRR1661284_megahit.fa	228	2110968	2110968	0	0	500	9258	66298	19113	SRR1661284_spades.fa	135	2158634	2158633	1	0	128	15989	207943	47788
SRR1661285	PRJNA266539	1329756	399781177	52.4	59	300	301	301	33	33.7	175x	SRR1661285_megahit.fa	206	2089668	2089668	0	0	502	10144	106639	20650	SRR1661285_spades.fa	136	2176457	2176457	0	0	128	16003	208138	80330
SRR1661286	PRJNA266539	1334210	401120790	52.3	40	300	301	301	33	33.6	176x	SRR1661286_megahit.fa	228	2095253	2095253	0	0	500	9189	63335	20054	SRR1661286_spades.fa	149	2181667	2181667	0	0	128	14642	293123	72289
SRR1661287	PRJNA266539	1087390	326914766	52.4	42	300	301	301	33	33.5	143x	SRR1661287_megahit.fa	197	2092789	2092789	0	0	508	10623	72577	22821	SRR1661287_spades.fa	163	2187777	2187777	0	0	128	13421	352939	72289
SRR1661288	PRJNA266539	933512	280664223	52.5	51	300	301	301	33	33.4	123x	SRR1661288_megahit.fa	192	2106144	2106144	0	0	501	10969	63369	22872	SRR1661288_spades.fa	144	2175418	2175418	0	0	128	15107	293219	80283
SRR1661289	PRJNA266539	1244268	374096108	52.4	60	300	301	301	33	33.3	164x	SRR1661289_megahit.fa	211	2060626	2060626	0	0	504	9766	65085	21392	SRR1661289_spades.fa	193	2168093	2168093	0	0	128	11233	209200	80643
SRR1661290	PRJNA266539	349802	105170774	52.5	67	300	301	301	33	32.8	46x	SRR1661290_megahit.fa	166	2043403	2043403	0	0	501	12309	67477	28967	SRR1661290_spades.fa	142	2110662	2110662	0	0	128	14863	207780	67074
SRR1661291	PRJNA266539	832810	250385786	52.7	46	300	301	301	33	33.7	110x	SRR1661291_megahit.fa	180	2046326	2046326	0	0	501	11368	81959	23294	SRR1661291_spades.fa	127	2109294	2109294	0	0	128	16608	211357	73239
SRR1661292	PRJNA266539	972638	170519636	51.9	35	175	251	251	33	31.4	74x	SRR1661292_megahit.fa	227	2112511	2112511	0	0	501	9306	67277	18746	SRR1661292_spades.fa	179	2167862	2167860	2	0	128	12110	208040	48618
SRR1661293	PRJNA266539	1210096	271004104	53.7	35	223	251	251	33	33.3	119x	SRR1661293_megahit.fa	499	2000865	2000865	0	0	506	4009	25507	7701	SRR1661293_spades.fa	193	2113119	2113119	0	0	128	10948	255908	55278
SRR1661294	PRJNA266539	1578040	375605490	52.2	35	238	251	251	33	32.9	165x	SRR1661294_megahit.fa	305	2098218	2098218	0	0	502	6879	44021	14204	SRR1661294_spades.fa	200	2182014	2182014	0	0	128	10910	207869	59758
SRR1661295	PRJNA266539	1147362	344947350	52.6	35	300	301	301	33	33.6	151x	SRR1661295_megahit.fa	189	2047477	2047477	0	0	503	10833	64295	25084	SRR1661295_spades.fa	164	2139600	2139600	0	0	128	13046	207927	66020
SRR1661296	PRJNA266539	2001476	470257536	52.6	35	234	251	251	33	33.6	206x	SRR1661296_megahit.fa	673	2044458	2044458	0	0	503	3037	31799	4922	SRR1661296_spades.fa	167	2159688	2159688	0	0	128	12932	207917	80506
SRR1661297	PRJNA266539	1372806	330730991	52.5	35	240	251	251	33	33.2	145x	SRR1661297_megahit.fa	281	2031701	2031701	0	0	504	7230	53981	14951	SRR1661297_spades.fa	235	2115362	2115361	1	0	128	9001	255825	56935
SRR1661298	PRJNA266539	1803828	423476092	52.7	35	234	251	251	33	32.1	186x	SRR1661298_megahit.fa	485	2097645	2097645	0	0	500	4325	34080	7512	SRR1661298_spades.fa	217	2196623	2196622	1	0	128	10122	207820	60868
SRR1661299	PRJNA266539	1298774	304371642	52.6	35	234	251	251	33	31.9	133x	SRR1661299_megahit.fa	321	2105453	2105453	0	0	502	6559	62404	13112	SRR1661299_spades.fa	181	2187984	2187984	0	0	128	12088	209815	65698
SRR1661300	PRJNA266539	799612	189867878	52.3	35	237	251	251	33	31.4	83x	SRR1661300_megahit.fa	228	2050677	2050677	0	0	500	8994	66394	19485	SRR1661300_spades.fa	273	2134893	2134893	0	0	126	7820	255984	55278
SRR1661301	PRJNA266539	1354114	324130564	52	35	239	251	251	33	32.1	142x	SRR1661301_megahit.fa	269	2127091	2127091	0	0	500	7907	66307	19499	SRR1661301_spades.fa	694	2303701	2303687	14	0	128	3319	208024	49225
SRR1661302	PRJNA266539	1426518	341777271	52.2	35	239	251	251	33	32.8	150x	SRR1661302_megahit.fa	316	2100721	2100721	0	0	500	6647	61822	15550	SRR1661302_spades.fa	230	2188649	2188649	0	0	128	9515	207961	66302
SRR1661303	PRJNA266539	1493264	356552152	52.3	35	238	251	251	33	33.5	156x	SRR1661303_megahit.fa	232	2026872	2026872	0	0	504	8736	52786	18240	SRR1661303_spades.fa	318	2132840	2132839	1	0	128	6707	255794	53740
SRR1661304	PRJNA266539	1489426	341993646	52.5	35	229	251	251	33	33.4	150x	SRR1661304_megahit.fa	486	2081760	2081760	0	0	502	4283	43474	7643	SRR1661304_spades.fa	151	2178841	2178841	0	0	128	14429	209915	71054
SRR1661305	PRJNA266539	1235736	285387808	52.4	35	230	251	251	33	33	125x	SRR1661305_megahit.fa	291	2039288	2039288	0	0	501	7007	40861	15456	SRR1661305_spades.fa	256	2124058	2124058	0	0	128	8297	211359	57627
SRR1661306	PRJNA266539	835658	193107280	52.9	35	231	251	251	33	32	84x	SRR1661306_megahit.fa	325	2029770	2029770	0	0	501	6245	40854	13316	SRR1661306_spades.fa	233	2121613	2121613	0	0	128	9105	255821	57424
SRR1661307	PRJNA266539	1259570	309029042	53.5	35	245	251	251	33	31.8	135x	SRR1661307_megahit.fa	684	2036782	2036782	0	0	500	2977	22862	4942	SRR1661307_spades.fa	158	2188589	2188589	0	0	128	13851	207863	67469
SRR1661308	PRJNA266539	2030440	494949137	52.8	35	243	251	251	33	33	217x	SRR1661308_megahit.fa	735	2040128	2040128	0	0	504	2775	25034	4293	SRR1661308_spades.fa	161	2185470	2185470	0	0	128	13574	207860	67737
SRR1661309	PRJNA266539	1800678	434422666	53.1	35	241	251	251	33	32.5	191x	SRR1661309_megahit.fa	892	2001025	2001025	0	0	500	2243	27130	3154	SRR1661309_spades.fa	161	2186953	2186953	0	0	128	13583	233509	78621
SRR1661310	PRJNA266539	1225910	302337940	53.6	35	246	251	251	33	31.8	132x	SRR1661310_megahit.fa	690	2036471	2036471	0	0	500	2951	37238	5847	SRR1661310_spades.fa	182	2185007	2185007	0	0	128	12005	207819	84847
SRR1661311	PRJNA266539	1085910	268685655	52.5	35	247	251	251	33	31.1	118x	SRR1661311_megahit.fa	911	2030705	2030705	0	0	500	2229	23368	3932	SRR1661311_spades.fa	290	2202067	2202067	0	0	128	7593	154782	60610
SRR1661312	PRJNA266539	1252252	307050016	53.9	35	245	251	251	33	32	135x	SRR1661312_megahit.fa	849	1993128	1993128	0	0	500	2347	20852	3680	SRR1661312_spades.fa	203	2171783	2171783	0	0	128	10698	207612	78215
SRR1661313	PRJNA266539	2502114	597457125	54	35	238	251	251	33	33.3	262x	SRR1661313_megahit.fa	1120	1726523	1726523	0	0	500	1541	15341	2035	SRR1661313_spades.fa	173	2135720	2135720	0	0	128	12345	208160	69670
SRR1661314	PRJNA266539	1932750	453547053	52.9	35	234	251	251	33	32.6	199x	SRR1661314_megahit.fa	597	2055449	2055449	0	0	500	3442	39039	5657	SRR1661314_spades.fa	150	2211889	2211889	0	0	128	14745	207828	60162
SRR1661315	PRJNA266539	1088842	264562597	53.1	35	242	251	251	33	30.1	116x	SRR1661315_megahit.fa	290	2109823	2109823	0	0	502	7275	41167	16593	SRR1661315_spades.fa	189	2196132	2196132	0	0	128	11619	208021	80311
SRR1661316	PRJNA266539	808106	194632645	54.9	35	240	251	251	33	29.8	85x	SRR1661316_megahit.fa	326	2050641	2050641	0	0	502	6290	51542	12458	SRR1661316_spades.fa	197	2144341	2144341	0	0	128	10884	219704	55247
SRR1661317	PRJNA266539	1223084	367715943	52.6	35	300	301	301	33	33.7	161x	SRR1661317_megahit.fa	204	2035500	2035500	0	0	502	9977	66248	21154	SRR1661317_spades.fa	173	2122607	2122607	0	0	128	12269	257229	60658
SRR1661318	PRJNA266539	1163444	349775870	52.4	49	300	301	301	33	33.4	153x	SRR1661318_megahit.fa	202	2101805	2101805	0	0	502	10404	64460	25254	SRR1661318_spades.fa	160	2186931	2186931	0	0	128	13668	294026	85032
SRR1661319	PRJNA266539	1095460	329354328	52.4	54	300	301	301	33	33.5	144x	SRR1661319_megahit.fa	226	2094776	2094776	0	0	500	9268	53237	21153	SRR1661319_spades.fa	144	2179588	2179588	0	0	128	15136	211771	71855
SRR1661320	PRJNA266539	1135074	341250564	52.4	37	300	301	301	33	33.5	150x	SRR1661320_megahit.fa	204	2100985	2100985	0	0	501	10298	134400	20940	SRR1661320_spades.fa	170	2178677	2178677	0	0	128	12815	256631	66452
SRR1661321	PRJNA266539	1161688	349256102	52.4	35	300	301	301	33	33.7	153x	SRR1661321_megahit.fa	216	2101287	2101287	0	0	500	9728	84770	21849	SRR1661321_spades.fa	165	2183894	2183894	0	0	128	13235	207750	70388
SRR1661322	PRJNA266539	1002232	301303790	53	35	300	301	301	33	31.1	132x	SRR1661322_megahit.fa	248	2039638	2039638	0	0	506	8224	40729	18201	SRR1661322_spades.fa	395	2258992	2258992	0	0	128	5718	227088	63474
SRR1661323	PRJNA266539	1062932	319568851	52.4	49	300	301	301	33	33.8	140x	SRR1661323_megahit.fa	205	2063707	2063707	0	0	502	10066	64450	21264	SRR1661323_spades.fa	167	2139739	2139739	0	0	128	12812	110093	57887
SRR1661324	PRJNA266539	1038480	312223359	52.2	35	300	301	301	33	33.5	137x	SRR1661324_megahit.fa	211	2140634	2140634	0	0	500	10145	64282	20176	SRR1661324_spades.fa	186	2236265	2236265	0	0	128	12022	205339	69739
SRR1661325	PRJNA266539	1265920	380601529	52.4	37	300	301	301	33	33.5	167x	SRR1661325_megahit.fa	207	2088886	2088886	0	0	500	10091	95187	23045	SRR1661325_spades.fa	151	2180163	2180163	0	0	128	14438	218311	77025
SRR1661326	PRJNA266539	1507178	453125729	52.4	44	300	301	301	33	34.1	199x	SRR1661326_megahit.fa	238	2037718	2037718	0	0	500	8561	67522	18751	SRR1661326_spades.fa	173	2138584	2138584	0	0	128	12361	249289	68017
SRR1661327	PRJNA266539	1424480	428275275	52.2	36	300	301	301	33	33.5	188x	SRR1661327_megahit.fa	260	2072204	2072204	0	0	502	7970	97923	18621	SRR1661327_spades.fa	185	2176605	2176605	0	0	128	11765	170277	57777
SRR1661328	PRJNA266539	1141286	343117998	51.8	35	300	301	301	33	33.7	150x	SRR1661328_megahit.fa	203	2097106	2097106	0	0	505	10330	64610	25132	SRR1661328_spades.fa	150	2186357	2186357	0	0	128	14575	207674	71797
SRR1661329	PRJNA266539	1165844	350497087	51.3	35	300	301	301	33	34	154x	SRR1661329_megahit.fa	210	2096326	2096326	0	0	500	9982	63361	22858	SRR1661329_spades.fa	164	2182411	2182411	0	0	128	13307	207984	57261
SRR1661330	PRJNA266539	1184292	356044300	50.7	63	300	301	301	33	33.7	156x	SRR1661330_megahit.fa	206	2093993	2093993	0	0	511	10165	67047	23347	SRR1661330_spades.fa	147	2177465	2177465	0	0	128	14812	212029	66884
SRR1661331	PRJNA266539	918690	276206451	52.7	53	300	301	301	33	33.6	121x	SRR1661331_megahit.fa	193	2061183	2061183	0	0	509	10679	95052	30250	SRR1661331_spades.fa	163	2136637	2136637	0	0	128	13108	207969	64086
SRR2736093	PRJNA298332	1344534	404257378	51.9	44	300	301	301	33	31	177x	SRR2736093_megahit.fa	235	2101074	2101074	0	0	500	8940	89854	21188	SRR2736093_spades.fa	98	2156409	2156403	6	0	128	22004	207542	72678
SRR2736094	PRJNA298332	1104120	331954164	51.9	35	300	301	301	33	31.3	146x	SRR2736094_megahit.fa	208	2102017	2102017	0	0	506	10105	67264	26132	SRR2736094_spades.fa	107	2159663	2159662	1	0	128	20183	208100	70223
SRR2736095	PRJNA298332	1312238	394544238	51.9	47	300	301	301	33	31.3	173x	SRR2736095_megahit.fa	226	2096087	2096087	0	0	500	9274	64538	20872	SRR2736095_spades.fa	90	2158425	2158418	7	0	128	23982	207800	76588
SRR2736096	PRJNA298332	1097698	330033013	51.8	42	300	301	301	33	31.3	145x	SRR2736096_megahit.fa	211	2106098	2106098	0	0	505	9981	62924	20909	SRR2736096_spades.fa	116	2171000	2171000	0	0	128	18715	208030	66127
SRR2736097	PRJNA298332	1068594	321292357	52	49	300	301	301	33	31.1	141x	SRR2736097_megahit.fa	221	2106797	2106797	0	0	503	9533	66207	23014	SRR2736097_spades.fa	99	2162006	2162004	2	0	128	21838	207655	72478
SRR2736098	PRJNA298332	1263574	379912483	52	35	300	301	301	33	30.6	167x	SRR2736098_megahit.fa	234	2096058	2096058	0	0	500	8957	75611	22173	SRR2736098_spades.fa	102	2162785	2162777	8	0	128	21203	207896	76061
SRR2736099	PRJNA298332	1052148	316338047	52	44	300	301	301	33	31	139x	SRR2736099_megahit.fa	217	2100758	2100758	0	0	502	9680	66157	22801	SRR2736099_spades.fa	114	2164463	2164463	0	0	128	18986	207694	66888
SRR2736100	PRJNA298332	757042	227610333	51.9	51	300	301	301	33	31.3	100x	SRR2736100_megahit.fa	192	2110927	2110927	0	0	504	10994	67275	25396	SRR2736100_spades.fa	98	2159754	2159731	23	0	128	22038	208179	72478
SRR2736101	PRJNA298332	912632	274399852	52	37	300	301	301	33	31.2	120x	SRR2736101_megahit.fa	199	2103871	2103871	0	0	500	10572	105251	27235	SRR2736101_spades.fa	96	2160853	2160841	12	0	128	22508	229079	88928
SRR2736102	PRJNA298332	1143668	343851340	52	55	300	301	301	33	30.5	151x	SRR2736102_megahit.fa	224	2111672	2111672	0	0	500	9427	64475	27060	SRR2736102_spades.fa	107	2160338	2160337	1	0	128	20190	229039	83897
SRR2736103	PRJNA298332	1071128	322058623	52	59	300	301	301	33	30.8	141x	SRR2736103_megahit.fa	222	2096125	2096125	0	0	500	9442	64219	22183	SRR2736103_spades.fa	107	2163028	2163003	25	0	128	20215	231178	76532
SRR2736104	PRJNA298332	1286000	386649903	51.9	38	300	301	301	33	31	170x	SRR2736104_megahit.fa	222	2090276	2090276	0	0	500	9415	67021	21076	SRR2736104_spades.fa	96	2157275	2157265	10	0	128	22471	207821	70226
SRR2736105	PRJNA298332	1248376	375355021	51.9	35	300	301	301	33	30.6	165x	SRR2736105_megahit.fa	238	2106522	2106522	0	0	500	8850	93123	20768	SRR2736105_spades.fa	105	2166281	2166271	10	0	128	20631	207849	78713
SRR2736106	PRJNA298332	1334994	401393849	52	44	300	301	301	33	31	176x	SRR2736106_megahit.fa	242	2101941	2101941	0	0	502	8685	64544	21192	SRR2736106_spades.fa	95	2154679	2154677	2	0	128	22680	222496	87401
SRR2736107	PRJNA298332	1191650	358290616	52	47	300	301	301	33	31.1	157x	SRR2736107_megahit.fa	212	2100370	2100370	0	0	500	9907	67262	21522	SRR2736107_spades.fa	102	2156615	2156615	0	0	128	21143	207821	60584
SRR2736108	PRJNA298332	1125400	338372499	52.1	71	300	301	301	33	30.9	148x	SRR2736108_megahit.fa	232	2108633	2108633	0	0	500	9088	59963	21054	SRR2736108_spades.fa	98	2155614	2155611	3	0	128	21996	207559	67495
SRR2736109	PRJNA298332	796814	239583174	52.1	56	300	301	301	33	30.6	105x	SRR2736109_megahit.fa	194	2111461	2111461	0	0	502	10883	80552	27183	SRR2736109_spades.fa	97	2158068	2158068	0	0	128	22248	207760	72478
SRR2736110	PRJNA298332	1204998	362310945	52.1	57	300	301	301	33	30.4	159x	SRR2736110_megahit.fa	236	2067712	2067712	0	0	500	8761	64430	21846	SRR2736110_spades.fa	110	2107812	2107810	2	0	128	19161	207896	73688
SRR2736111	PRJNA298332	1066586	320691882	52	53	300	301	301	33	30.6	141x	SRR2736111_megahit.fa	217	2062558	2062558	0	0	503	9504	95095	23990	SRR2736111_spades.fa	125	2110382	2110377	5	0	128	16883	207896	57179
SRR2736112	PRJNA298332	731956	220071291	52.1	47	300	301	301	33	31	96x	SRR2736112_megahit.fa	185	2110026	2110026	0	0	502	11405	66157	31034	SRR2736112_spades.fa	112	2157753	2157753	0	0	128	19265	207559	66888
SRR2736113	PRJNA298332	857982	257966986	52	35	300	301	301	33	31.2	113x	SRR2736113_megahit.fa	185	2103967	2103967	0	0	500	11372	123993	23045	SRR2736113_spades.fa	102	2156622	2156611	11	0	128	21143	207759	70224
SRR2736114	PRJNA298332	845522	254220005	52	35	300	301	301	33	30.7	111x	SRR2736114_megahit.fa	204	2122855	2122855	0	0	500	10406	105244	25144	SRR2736114_spades.fa	115	2168001	2168000	1	0	128	18852	208678	70398
SRR2736115	PRJNA298332	1123772	337882078	52	52	300	301	301	33	31.1	148x	SRR2736115_megahit.fa	227	2105096	2105096	0	0	500	9273	82989	23197	SRR2736115_spades.fa	117	2158760	2158744	16	0	128	18450	207730	70448
SRR2736116	PRJNA298332	1198356	360294275	51.8	35	300	301	301	33	31.2	158x	SRR2736116_megahit.fa	209	2103427	2103427	0	0	503	10064	75781	23249	SRR2736116_spades.fa	117	2154331	2154329	2	0	128	18413	207547	67537
SRR2736117	PRJNA298332	1199054	360504854	52	53	300	301	301	33	30.6	158x	SRR2736117_megahit.fa	215	2099863	2099863	0	0	500	9766	67201	23211	SRR2736117_spades.fa	97	2155422	2155417	5	0	128	22220	231195	75937
SRR2736118	PRJNA298332	1111452	334171493	52.1	48	300	301	301	33	30.9	146x	SRR2736118_megahit.fa	208	2048934	2048934	0	0	505	9850	66271	23143	SRR2736118_spades.fa	106	2106384	2106384	0	0	128	19871	211750	63648
SRR2736119	PRJNA298332	1131902	340325138	52	46	300	301	301	33	30.8	149x	SRR2736119_megahit.fa	218	2100768	2100768	0	0	502	9636	64548	23006	SRR2736119_spades.fa	103	2160037	2160037	0	0	128	20971	231195	70223
SRR2736120	PRJNA298332	1034900	311146007	52.1	53	300	301	301	33	30.9	136x	SRR2736120_megahit.fa	208	2101183	2101183	0	0	502	10101	67235	22182	SRR2736120_spades.fa	99	2158037	2158036	1	0	128	21798	207635	75937
SRR2736121	PRJNA298332	878630	264173568	52.1	35	300	301	301	33	30.8	116x	SRR2736121_megahit.fa	186	2105624	2105624	0	0	501	11320	105187	27317	SRR2736121_spades.fa	98	2156097	2156085	12	0	128	22000	207635	75937
SRR2736122	PRJNA298332	877880	263948914	52.1	38	300	301	301	33	30.2	116x	SRR2736122_megahit.fa	198	2105497	2105497	0	0	500	10633	67243	29533	SRR2736122_spades.fa	118	2160409	2160386	23	0	128	18308	207635	60601
SRR2736123	PRJNA298332	929310	279403181	52	58	300	301	301	33	30.9	122x	SRR2736123_megahit.fa	207	2104416	2104416	0	0	500	10166	66207	27037	SRR2736123_spades.fa	114	2158585	2158581	4	0	128	18934	207761	66787
SRR2736124	PRJNA298332	755584	227170022	52	58	300	301	301	33	31	99x	SRR2736124_megahit.fa	189	2111046	2111046	0	0	500	11169	139659	30662	SRR2736124_spades.fa	86	2156522	2156501	21	0	128	25075	224732	84136
SRR2736125	PRJNA298332	1297552	217605147	51.8	35	167	251	251	33	29.4	95x	SRR2736125_megahit.fa	198	2102321	2102321	0	0	506	10617	158918	22894	SRR2736125_spades.fa	194	2162593	2162592	1	0	128	11147	207829	43559
SRR2736126	PRJNA298332	2972622	480756677	51.7	35	161	251	251	33	27.6	211x	SRR2736126_megahit.fa	334	2087591	2087591	0	0	500	6250	43981	11091	SRR2736126_spades.fa	141	2158368	2158365	3	0	128	15307	207815	52869
SRR2736127	PRJNA298332	1187468	203700734	51.6	35	171	251	251	33	30.8	89x	SRR2736127_megahit.fa	220	2095467	2095467	0	0	501	9524	87596	22903	SRR2736127_spades.fa	159	2149074	2149073	1	0	128	13516	208357	50098
SRR2736128	PRJNA298332	712264	130639770	52.1	35	183	251	251	33	30.3	57x	SRR2736128_megahit.fa	197	2109275	2109275	0	0	501	10706	105337	20784	SRR2736128_spades.fa	161	2164005	2164005	0	0	128	13441	152730	47029
SRR2736129	PRJNA298332	996844	177592594	52	35	178	251	251	33	30.2	78x	SRR2736129_megahit.fa	189	2108436	2108436	0	0	507	11155	172523	23264	SRR2736129_spades.fa	158	2156578	2156574	4	0	128	13649	231090	53701
SRR2736130	PRJNA298332	426812	84001209	52.2	35	196	251	251	33	29.7	36x	SRR2736130_megahit.fa	237	2097914	2097914	0	0	506	8851	53071	18531	SRR2736130_spades.fa	215	2160028	2160027	1	0	128	10046	176585	41254
SRR2736131	PRJNA298332	1056474	317636336	52.7	35	300	301	301	33	30	139x	SRR2736131_megahit.fa	234	2053160	2053160	0	0	500	8774	41683	20667	SRR2736131_spades.fa	92	2113027	2113027	0	0	128	22967	264832	81624
SRR2736132	PRJNA298332	594084	112689042	52.2	35	189	251	251	33	30.2	49x	SRR2736132_megahit.fa	198	2108569	2108569	0	0	511	10649	67305	20914	SRR2736132_spades.fa	181	2162984	2162984	0	0	128	11950	199490	47510
SRR2736133	PRJNA298332	543616	105607644	52.2	35	194	251	251	33	29.4	46x	SRR2736133_megahit.fa	208	2067095	2067095	0	0	501	9937	62994	20406	SRR2736133_spades.fa	157	2116503	2116502	1	0	128	13480	108646	47286
SRR2736134	PRJNA298332	1996802	331979770	49.8	35	166	251	251	33	30.2	146x	SRR2736134_megahit.fa	234	2084347	2084347	0	0	506	8907	66373	19966	SRR2736134_spades.fa	165	2166176	2166175	1	0	128	13128	174266	53019
SRR2736135	PRJNA298332	1026828	308730633	52.5	35	300	301	301	33	30.4	135x	SRR2736135_megahit.fa	219	2102979	2102979	0	0	501	9602	105238	22936	SRR2736135_spades.fa	79	2162066	2162066	0	0	128	27367	246443	86229
SRR2736136	PRJNA298332	1025008	308167820	52.7	35	300	301	301	33	30.5	135x	SRR2736136_megahit.fa	207	2047405	2047405	0	0	501	9890	66173	23210	SRR2736136_spades.fa	91	2113766	2113766	0	0	128	23228	260016	81204
SRR2736137	PRJNA298332	1018546	306242510	52.7	35	300	301	301	33	30.4	134x	SRR2736137_megahit.fa	227	2057355	2057355	0	0	500	9063	95095	19823	SRR2736137_spades.fa	93	2113778	2113778	0	0	128	22728	260006	95180
SRR2736138	PRJNA298332	504764	100545290	52.1	35	199	251	251	33	28.1	44x	SRR2736138_megahit.fa	212	2120242	2120242	0	0	507	10001	105253	19290	SRR2736138_spades.fa	137	2159248	2159244	4	0	128	15760	176332	49738
SRR2736139	PRJNA298332	921200	155238093	51.9	35	168	251	251	33	29.9	68x	SRR2736139_megahit.fa	188	2119067	2119067	0	0	504	11271	87880	24833	SRR2736139_spades.fa	155	2166964	2166964	0	0	128	13980	172777	45996
SRR2736140	PRJNA298332	851240	255925973	52.7	35	300	301	301	33	29.7	112x	SRR2736140_megahit.fa	211	2062848	2062848	0	0	501	9776	67148	23048	SRR2736140_spades.fa	102	2116525	2116525	0	0	128	20750	207957	73687
SRR2736141	PRJNA298332	1813800	343566449	51.7	35	189	251	251	33	33.2	151x	SRR2736141_megahit.fa	282	2099171	2099171	0	0	501	7443	53656	14368	SRR2736141_spades.fa	159	2168990	2168990	0	0	128	13641	207904	49072
SRR2736142	PRJNA298332	722802	217315125	52.4	38	300	301	301	33	30.1	95x	SRR2736142_megahit.fa	210	2165873	2165873	0	0	501	10313	66367	23292	SRR2736142_spades.fa	82	2221133	2221133	0	0	128	27086	211092	95490
SRR2736143	PRJNA298332	844896	254016847	52.5	35	300	301	301	33	30.4	111x	SRR2736143_megahit.fa	205	2110176	2110176	0	0	500	10293	67479	21260	SRR2736143_spades.fa	132	2171300	2171300	0	0	128	16449	207896	59817
SRR2736144	PRJNA298332	1597400	480218207	52.6	43	300	301	301	33	32.3	211x	SRR2736144_megahit.fa	490	2036174	2036174	0	0	501	4155	31916	7869	SRR2736144_spades.fa	112	2114141	2114141	0	0	128	18876	207944	81217
SRR2736145	PRJNA298332	1829244	549963044	52.7	48	300	301	301	33	31.6	241x	SRR2736145_megahit.fa	736	2037933	2037933	0	0	500	2768	26107	5321	SRR2736145_spades.fa	100	2112197	2112197	0	0	128	21121	363326	85676
SRR2736146	PRJNA298332	1500096	450972405	52.5	35	300	301	301	33	32.3	198x	SRR2736146_megahit.fa	355	2088832	2088832	0	0	500	5884	49037	11750	SRR2736146_spades.fa	125	2164105	2164105	0	0	128	17312	207848	59146
SRR2736147	PRJNA298332	1487706	447241232	52.6	61	300	301	301	33	32.3	196x	SRR2736147_megahit.fa	402	2107164	2107164	0	0	500	5241	40814	11108	SRR2736147_spades.fa	77	2166591	2166591	0	0	128	28137	246406	128217
SRR2736148	PRJNA298332	1570904	472186389	45.7	35	300	301	301	33	32.7	207x	SRR2736148_megahit.fa	353	4081325	4081325	0	0	503	11561	98670	27067	SRR2736148_spades.fa	474	4323043	4323043	0	0	128	9120	211317	72670
SRR2736149	PRJNA298332	919176	276313525	51.9	45	300	301	301	33	32.1	121x	SRR2736149_megahit.fa	229	2097825	2097825	0	0	501	9160	67453	21998	SRR2736149_spades.fa	127	2169457	2169457	0	0	128	17082	193209	80793
SRR2736150	PRJNA298332	1384718	416297657	52.3	41	300	301	301	33	32.5	183x	SRR2736150_megahit.fa	306	2105179	2105179	0	0	500	6879	43002	13793	SRR2736150_spades.fa	88	2166480	2166480	0	0	128	24619	246020	88988

SRR2736151	PRJNA298332	1519096	456687123	52.5	61	300	301	301	33	31.6	200x	SRR2736151_megahit.fa	493	2121330	2121330	0	0	500	4302	36584	9611	SRR2736151_spades.fa	78	2164015	2164015	0	0	128	27743	207676	78678
SRR2736152	PRJNA298332	1467564	441217415	52.7	35	300	301	301	33	31.7	194x	SRR2736152_megahit.fa	387	2038607	2038607	0	0	500	5267	36305	10582	SRR2736152_spades.fa	113	2115452	2115452	0	0	128	18720	240017	81271
SRR2736153	PRJNA298332	1227512	369061823	52.7	51	300	301	301	33	32	162x	SRR2736153_megahit.fa	295	2047981	2047981	0	0	500	6942	46747	16146	SRR2736153_spades.fa	109	2113736	2113736	0	0	128	19392	245677	81750
SRR2736154	PRJNA298332	1645476	494706056	52.7	44	300	301	301	33	31.9	217x	SRR2736154_megahit.fa	529	2102439	2102439	0	0	500	3974	37473	7916	SRR2736154_spades.fa	96	2172083	2172083	0	0	128	22625	241912	106649
SRR2736155	PRJNA298332	1640698	409916186	48.9	35	249	251	251	33	34.5	180x	SRR2736155_megahit.fa	416	4044606	4044606	0	0	502	9722	147648	20792	SRR2736155_spades.fa	240	4155074	4155074	0	0	128	17312	211199	68692
SRR2736156	PRJNA298332	1656754	498109519	52.6	57	300	301	301	33	31.5	219x	SRR2736156_megahit.fa	588	2137606	2137606	0	0	500	3635	35737	7545	SRR2736156_spades.fa	109	2199990	2199990	0	0	128	20183	211453	76711
SRR2736157	PRJNA298332	1096514	212722997	51.8	35	193	251	251	33	28.7	93x	SRR2736157_megahit.fa	212	2113995	2113995	0	0	506	9971	64515	20941	SRR2736157_spades.fa	160	2168588	2168588	0	0	128	13553	208517	50264
SRR2736158	PRJNA298332	416754	81342086	52	35	195	251	251	33	29.4	35x	SRR2736158_megahit.fa	252	2117540	2117540	0	0	506	8402	49514	17699	SRR2736158_spades.fa	100	2129272	2129266	6	0	128	21292	172772	44243
SRR2736159	PRJNA298332	1197966	217567849	52.3	35	181	251	251	33	29.8	95x	SRR2736159_megahit.fa	219	2063442	2063442	0	0	507	9422	67172	20879	SRR2736159_spades.fa	150	2114896	2114896	0	0	128	14099	208097	55455
SRR2736160	PRJNA298332	1288356	321064144	51.8	35	249	251	251	33	33.8	141x	SRR2736160_megahit.fa	328	2099249	2099249	0	0	503	6400	61796	12167	SRR2736160_spades.fa	123	2162512	2162512	0	0	128	17581	152889	65940
SRR2736161	PRJNA298332	1721250	429866698	52.2	35	249	251	251	33	33.2	189x	SRR2736161_megahit.fa	597	2074118	2074118	0	0	500	3474	38647	6262	SRR2736161_spades.fa	129	2162105	2162105	0	0	128	16760	207901	66461
SRR2736162	PRJNA298332	1837954	552567398	52.6	49	300	301	301	33	31.3	243x	SRR2736162_megahit.fa	672	2113754	2113754	0	0	500	3145	35575	6235	SRR2736162_spades.fa	101	2158182	2158182	0	0	128	21368	207853	70869
SRR2736163	PRJNA298332	1789984	538142331	52.3	50	300	301	301	33	31.9	236x	SRR2736163_megahit.fa	519	2097362	2097362	0	0	500	4041	30029	7290	SRR2736163_spades.fa	97	2157318	2157318	0	0	128	22240	207872	75004
SRR2736164	PRJNA298332	2071284	387729408	51.6	35	187	251	251	33	33.5	170x	SRR2736164_megahit.fa	266	2097918	2097918	0	0	504	7886	66366	16080	SRR2736164_spades.fa	173	2168277	2168277	0	0	128	12533	207881	49437
SRR2736165	PRJNA298332	479912	89429472	52.2	35	186	251	251	33	30.2	39x	SRR2736165_megahit.fa	211	2116736	2116736	0	0	501	10031	66248	19499	SRR2736165_spades.fa	158	2162558	2162554	4	0	128	13687	99562	47072
SRR2736166	PRJNA298332	533426	101554321	52.2	35	190	251	251	33	29.8	44x	SRR2736166_megahit.fa	212	2118794	2118794	0	0	508	9994	67544	20492	SRR2736166_spades.fa	165	2169417	2169416	1	0	128	13147	152584	47018
SRR2736167	PRJNA298332	1620152	487074471	52.4	35	300	301	301	33	32	214x	SRR2736167_megahit.fa	525	2101308	2101308	0	0	500	4002	43965	7619	SRR2736167_spades.fa	104	2170378	2170378	0	0	128	20869	207856	67805
SRR2736168	PRJNA298332	708436	126233576	51.6	35	178	251	251	33	30.2	55x	SRR2736168_megahit.fa	220	2117156	2117156	0	0	525	9623	72853	20476	SRR2736168_spades.fa	199	2167966	2167964	2	0	128	10894	172703	42278
SRR2736169	PRJNA298332	732086	182470072	51.3	35	249	251	251	33	33.6	80x	SRR2736169_megahit.fa	245	2150073	2150073	0	0	503	8775	65929	18536	SRR2736169_spades.fa	179	2209558	2209558	0	0	128	12343	210760	67307
SRR2736170	PRJNA298332	831262	152080867	51.8	35	182	251	251	33	30.2	66x	SRR2736170_megahit.fa	213	2149316	2149316	0	0	510	10090	65929	21697	SRR2736170_spades.fa	807	2314634	2314634	0	0	128	2868	226395	48178
SRR2736171	PRJNA298332	1636138	491892778	52.3	35	300	301	301	33	31.9	216x	SRR2736171_megahit.fa	459	2112950	2112950	0	0	500	4603	37859	10405	SRR2736171_spades.fa	105	2201940	2201940	0	0	128	20970	210659	81612
SRR2736172	PRJNA298332	1011114	303965638	52.6	35	300	301	301	33	31.8	133x	SRR2736172_megahit.fa	235	2141800	2141800	0	0	500	9114	68049	20850	SRR2736172_spades.fa	116	2203240	2203240	0	0	128	18993	210850	81031
SRR2736173	PRJNA298332	1288212	387289711	52.5	56	300	301	301	33	31.4	170x	SRR2736173_megahit.fa	331	2129001	2129001	0	0	500	6432	83448	17181	SRR2736173_spades.fa	109	2202127	2202127	0	0	128	20203	210616	77908
SRR2736174	PRJNA298332	1405406	234414921	51.8	35	166	251	251	33	29.5	103x	SRR2736174_megahit.fa	223	2142339	2142339	0	0	502	9606	84749	22638	SRR2736174_spades.fa	162	2203442	2203442	0	0	128	13601	207731	53641
SRR2736175	PRJNA298332	1317740	396132873	52.3	58	300	301	301	33	31.6	174x	SRR2736175_megahit.fa	640	2072783	2072783	0	0	500	3238	25110	5654	SRR2736175_spades.fa	108	2171309	2171309	0	0	128	20104	249137	79019
SRR2736176	PRJNA298332	1012594	304412267	52.6	53	300	301	301	33	31.8	133x	SRR2736176_megahit.fa	273	2142854	2142854	0	0	500	7849	49531	18673	SRR2736176_spades.fa	100	2203829	2203829	0	0	128	22038	210878	95224
SRR2736177	PRJNA298332	620934	121639302	50.8	35	195	251	251	33	29	53x	SRR2736177_megahit.fa	310	2203093	2203093	0	0	505	7106	63570	18622	SRR2736177_spades.fa	571	2427932	2427932	0	0	128	4252	162332	41420
SRR2736178	PRJNA298332	1514336	455298985	52.3	40	300	301	301	33	31.5	200x	SRR2736178_megahit.fa	376	2108154	2108154	0	0	500	5606	61644	14153	SRR2736178_spades.fa	112	2166821	2166819	2	0	128	19346	207886	60498
SRR2736179	PRJNA298332	800364	240596298	52.1	35	300	301	301	33	32.1	105x	SRR2736179_megahit.fa	208	2147783	2147783	0	0	504	10325	84706	24768	SRR2736179_spades.fa	109	2202007	2202006	1	0	128	20201	210868	82959
SRR2736180	PRJNA298332	1476640	443957835	52.1	35	300	301	301	33	31.9	195x	SRR2736180_megahit.fa	282	2114658	2114658	0	0	500	7498	84688	19474	SRR2736180_spades.fa	110	2200846	2200846	0	0	128	20007	210659	84645
SRR2736181	PRJNA298332	1373588	261331656	53.4	35	190	251	251	33	33.1	114x	SRR2736181_megahit.fa	354	2045168	2045168	0	0	510	5777	43983	12114	SRR2736181_spades.fa	152	2116550	2116550	0	0	128	13924	208125	55424
SRR2736182	PRJNA298332	1450742	436164253	52.2	35	300	301	301	33	32.2	191x	SRR2736182_megahit.fa	295	2136132	2136132	0	0	500	7241	41283	16926	SRR2736182_spades.fa	105	2203337	2203337	0	0	128	20984	210808	86752
SRR2736183	PRJNA298332	1284812	218376373	51.7	35	169	251	251	33	30.3	96x	SRR2736183_megahit.fa	213	2146450	2146450	0	0	505	10077	97914	20560	SRR2736183_spades.fa	149	2204219	2204216	3	0	128	14793	207680	53479
SRR2736184	PRJNA298332	715326	138530021	51.8	35	193	251	251	33	29.6	60x	SRR2736184_megahit.fa	213	2146453	2146453	0	0	511	10077	108087	20996	SRR2736184_spades.fa	185	2204728	2204728	0	0	82	11917	151864	46851
SRR2736185	PRJNA298332	1017328	230324263	52.8	35	226	251	251	33	34.3	101x	SRR2736185_megahit.fa	285	2111664	2111664	0	0	500	7409	41892	15053	SRR2736185_spades.fa	241	2180253	2180253	0	0	128	9046	180760	46976
SRR2736186	PRJNA298332	501592	92763373	51.6	35	184	251	251	33	28.6	40x	SRR2736186_megahit.fa	332	2129917	2129917	0	0	505	6415	38629	11585	SRR2736186_spades.fa	189	2166902	2166893	9	0	128	11465	105391	39905
SRR2736187	PRJNA298332	555658	108572250	51.9	35	195	251	251	33	29.7	47x	SRR2736187_megahit.fa	219	2064258	2064258	0	0	511	9425	67050	19499	SRR2736187_spades.fa	169	2118255	2118254	1	0	128	12534	211701	49660
SRR2736188	PRJNA298332	1123372	210678015	52	35	187	251	251	33	30.2	92x	SRR2736188_megahit.fa	205	2059832	2059832	0	0	501	10047	59874	22413	SRR2736188_spades.fa	149	2114401	2114397	4	0	128	14190	210891	60459
SRR2736189	PRJNA298332	1666552	501056911	52.3	39	300	301	301	33	31.5	220x	SRR2736189_megahit.fa	362	2077663	2077663	0	0	500	5739	44663	13072	SRR2736189_spades.fa	85	2108592	2108592	0	0	128	24806	208123	88427
SRR2736190	PRJNA298332	1397106	420030168	52.4	35	300	301	301	33	31.8	184x	SRR2736190_megahit.fa	293	2054923	2054923	0	0	500	7013	40435	16720	SRR2736190_spades.fa	101	2113977	2113977	0	0	128	20930	208123	84736
SRR2736191	PRJNA298332	1514606	455374186	52.3	35	300	301	301	33	31.8	200x	SRR2736191_megahit.fa	318	2074398	2074398	0	0	500	6523	52270	15158	SRR2736191_spades.fa	87	2108891	2108891	0	0	128	24240	208123	86273
SRR2736192	PRJNA298332	1443028	433855520	52.4	39	300	301	301	33	31.7	190x	SRR2736192_megahit.fa	328	2064552	2064552	0	0	500	6294	46945	15881	SRR2736192_spades.fa	100	2112436	2112432	4	0	128	21124	210596	81596
SRR2736193	PRJNA298332	2153804	408161943	51.9	35	189	251	251	33	33.5	179x	SRR2736193_megahit.fa	370	2092038	2092038	0	0	504	5654	37695	9897	SRR2736193_spades.fa	136	2167157	2167157	0	0	128	15934	207998	57137
SRR2736194	PRJNA298332	1005342	302239242	52.4	35	300	301	301	33	32	132x	SRR2736194_megahit.fa	214	2051436	2051436	0	0	500	9586	64487	20412	SRR2736194_spades.fa	98	2113274	2113262	12	0	128	21564	208123	76035
SRR2736195	PRJNA298332	1035168	311202251	52.3	36	300	301	301	33	31.4	136x	SRR2736195_megahit.fa	248	2065726	2065726	0	0	500	8329	105254	20603	SRR2736195_spades.fa	94	2112983	2112983	0	0	128	22478	208123	86241
SRR2736196	PRJNA298332	1190180	357815700	52.3	39	300	301	301	33	31.7	157x	SRR2736196_megahit.fa	276	2052369	2052369	0	0	502	7436	62886	17668	SRR2736196_spades.fa	104	2114904	2114904	0	0	128	20335	208123	76035
SRR2736197	PRJNA298332	920432	276719414	52.5	35	300	301	301	33	31.9	121x	SRR2736197_megahit.fa	207	2059595	2059595	0	0	501	9949	95095	23316	SRR2736197_spades.fa	97	2110527	2110527	0	0	128	21758	208123	83852
SRR2736198	PRJNA298332	996388	299547939	52.3	35	300	301	301	33	32.5	131x	SRR2736198_megahit.fa	213	2112450	2112450	0	0	501	9917	52293	22811	SRR2736198_spades.fa	122	2167731	2167731	0	0	128	17768	207841	70498
SRR2736199	PRJNA298332	413536	83174956	51.5	35	201	251	251	33	29	36x	SRR2736199_megahit.fa	279	2121616	2121616	0	0	515	7604	64515	16654	SRR2736199_spades.fa	377	2195551	2195537	14	0	128	5823	158893	47106
SRR2736200	PRJNA298332	917522	158718981	51.4	35	172	251	251	33	29	69x	SRR2736200_megahit.fa	212	2147244	2147244	0	0	513	10128	68587	21792	SRR2736200_spades.fa	221	2212026	2212024	2	0	128	10009	151864	44387
SRR2736201	PRJNA298332	1467476	242332914	51.7	35	165	251	251	33	29.3	106x	SRR2736201_megahit.fa	221	2141279	2141279	0	0	503	9689	65929	19728	SRR2736201_spades.fa	158	2201326	2201326	0	0	128	13932	207681	47834
SRR2736202	PRJNA298332	1168058	221078692	52.3	35	189	251	251	33	29.9	97x	SRR2736202_megahit.fa	212	2064524	2064524	0	0	514	9738	63487	22498	SRR2736202_spades.fa	142	2109936	2109934	2	0	128	14858	208025	55568
SRR2736203	PRJNA298332	600970	108170853	49.1	35	179	251	251	33	29.9	47x	SRR2736203_megahit.fa	304	2112065	2112065	0	0	500	6947	45264	13468	SRR2736203_spades.fa	373	2189790	2189790	0	0	128	5870	90652	35531
SRR2736204	PRJNA298332	889224	159243322	53.3	35	179	251	251	33	30.1	70x	SRR2736204_megahit.fa	200	2119380	2119380	0	0	500	10596	105267	23483	SRR2736204_spades.fa	583	2239529	2239528	1	0	128	3841	105365	48996
SRR2736205	PRJNA298332	821584	148333984	52.3	35	180	251	251	33	30.6	65x	SRR2736205_megahit.fa	192	2115676	2115676	0	0	541	11019	66248	21650	SRR2736205_spades.fa	990	2365637	2365619	18	0	128	2389	172853	41269
SRR2736206	PRJNA298332	1175004	353248002	52.4	44	300	301	301	33	31.9	155x	SRR2736206_megahit.fa	258	2063488	2063488	0	0	500	7998	66929	20194	SRR2736206_spades.fa	86	2113251	2113251	0	0	128	24572	208131	91244
SRR2736207	PRJNA298332	821056	246795475	52.3	35	300	301	301	33	32.3	108x	SRR2736207_megahit.fa	208	2063459	2063459	0	0	501	9920	105311	25087	SRR2736207_spades.fa	96	2113514	2113514	0	0	128	22015	245616	86342
SRR2736208	PRJNA298332	982334	295264956	52.2	35	300	301	301	33	32.6	129x	SRR2736208_megahit.fa	225	2061208	2061208	0	0	502	9160	82069	22113	SRR2736208_spades.fa	99	2115007	2115007	0	0	128	21363	208136	84736
SRR2736209	PRJNA298332	1103318	331689583	52.2	53	300	301	301	33	31.6	145x	SRR2736209_megahit.fa	233	2106465	2106465	0	0	503	9040	54085	20027	SRR2736209_spades.fa	96	2162728	2162725	3	0	128	22528	269752	88988
SRR2736210	PRJNA298332	892780	268364456	52.3	35	300	301	301	33	32.3	118x	SRR2736210_megahit.fa	218	2061736	2061736	0	0	500	9457	105230	21259	SRR2736210_spades.fa	101	2114390	2114390	0	0	128	20934	254264	81299
SRR2736211	PRJNA298332	1378880	336684043	54.1	35	244	251	251	33	32	148x	SRR2736211_megahit.fa	749	1952809	1952809	0	0	500	2607	29341	4497	SRR2736211_spades.fa	113	2117006	2117006	0	0	128	18734	207935	78459
SRR2736212	PRJNA298332	880962	264835681	51.9	50	300	301	301	33	32	116x	SRR2736212_megahit.fa	206	2062246	2062246	0	0	501	10010	95092	23376	SRR2736212_spades.fa	100	2108160	2108160	0	0	128	21081	169099	84614
SRR2736213	PRJNA298332	1072724	322495875	52.3	35	300	301	301	33	32.1	141x	SRR2736213_megahit.fa	221	2096356	2096356	0	0	501	9485	60877	19651	SRR2736213_spades.fa	118	2165959	2165959	0	0	128	18355	161971	76518
SRR2736214	PRJNA298332	1214432	291129909	52.1	35	239	251	251	33	33.8	128x	SRR2736214_megahit.fa	270	2108439	2108439	0	0	502	7809	43111	16590	SRR2736214_spades.fa	229	2183679	2183678	1	0	128	9535	211227	66899
SRR2736215	PRJNA298332	1620082	366032390	52.8	35	225	251	251	33	32.7	160x	SRR2736215_megahit.fa	293	2038797	2038797	0	0	500	6958	63895	13985	SRR2736215_spades.fa	207	2103563	2103563	0	0	128	10162	255869	55158
SRR2736216	PRJNA298332	1513178	344813340	53.2	35	227	251	251	33	32.3	151x	SRR2736216_megahit.fa	350	2028057	2028057	0	0	502	5794	54332	12080	SRR2736216_spades.fa	178	2100340	2100340	0	0	128	11799	207716	53325
SRR2736217	PRJNA298332	1965478	435853291	53.8	35	221	251	251	33	32.6	191x	SRR2736217_megahit.fa	702	1998666	1998666	0	0	500	2847	23459	4514	SRR2736217_spades.fa	132	2112274	2112274	0	0	128	16002	208118	67518
SRR2736218	PRJNA298332	913990	274775656	52.3	63	300	301	301	33	32	120x	SRR2736218_megahit.fa	415	2072240	2072240	0	0	501	4993	41671	13152	SRR2736218_spades.fa	116	2115274	2115274	0	0	128	18235	127505	76130
SRR2736219	PRJNA298332	952026	286188925	52.3	35	300	301	301	33	31.8	125x	SRR2736219_megahit.fa	211	2061172	2061172	0	0	501	9768	66980	22200	SRR2736219_spades.fa	96	2110424	2110424	0	0	128	21983	207910	75653
SRR2736220	PRJNA298332	1043656	313768066	52.4	42	300	301	301	33	31.4	138x	SRR2736220_megahit.fa	233	2103525	2103525	0	0	500	9028	64436	20744	SRR2736220_spades.fa	100	2155603	2155599	4	0	128	21556	207904	69097
SRR2736221	PRJNA298332	1358168	408326186	52.4	39	300	301	301	33	31.5	179x	SRR2736221_megahit.fa	267	2059450	2059450	0	0	500	7713	95095	19575	SRR2736221_spades.fa	94	2113515	2113515	0	0	128	22484	208108	91249
SRR2736222	PRJNA298332	887536	266804937	52.1	35	300	301	301	33	31.8	117x	SRR2736222_megahit.fa	200	2104776	2104776	0	0	503	10523	105246	23048	SRR2736222_spades.fa	97	2162892	2162892	0	0	128	22297	208054	85767
SRR2736223	PRJNA298332	671348	201813191	52.2	39	300	301	301	33	32	88x	SRR2736223_megahit.fa	198	2115770	2115770	0	0	501	10685	105271	26728	SRR2736223_spades.fa	104	2162916	2162916	0	0	128	20797	208101	67463
SRR2736224	PRJNA298332	801310	240901196	52.2	35	300	301	301	33	32	105x	SRR2736224_megahit.fa	211	2113667	2113667	0	0	500	10017	67224	23339	SRR2736224_spades.fa	95	2162170	2162170	0	0	128	22759	207961	67589
SRR2736225	PRJNA298332	1230138	369803622	52.6	51	300	301	301	33	33.1	162x	SRR2736225_megahit.fa	244	2053348	2053348	0	0	502	8415	66499	20252	SRR2736225_spades.fa	109	2113619	2113619	0	0	128	19391	308556	81515
SRR2736226	PRJNA298332	1361452	409304988	52.2	43	300	301	301	33	32.1	180x	SRR2736226_megahit.fa	272	2092213	2092213	0	0	500	7691	40507	18477	SRR2736226_spades.fa	103	2163477	2163477	0	0	128	21004	363720	71270
SRR2736227	PRJNA298332	1403926	422081882	52.2	38	300	301	301	33	31.6	185x	SRR2736227_megahit.fa	288	2109067	2109067	0	0	500	7323	63183	19184	SRR2736227_spades.fa	101	2163667	2163667	0	0	128	21422	207968	66302
SRR2736228	PRJNA298332	1406004	422683005	52.4	38	300	301	301	33	33	185x	SRR2736228_megahit.fa	282	2101119	2101119	0	0	500	7450	61929	18394	SRR2736228_spades.fa	94	2165245	2165245	0	0	128	23034	207968	81638
SRR2736229	PRJNA298332	1267598	381091135	52.2	50	300	301	301	33	31.9	167x	SRR2736229_megahit.fa	268	2107732	2107732	0	0	500	7864	56861	20370	SRR2736229_spades.fa	92	2164055	2164055	0	0	128	23522	245722	85445
SRR2736230	PRJNA298332	1099918	330679648	52.3	43	300	301	301	33	31.6	145x	SRR2736230_megahit.fa	225	2105329	2105329	0	0	500	9357	66262	23008	SRR2736230_spades.fa	100	2162478	2162477	1	0	128	21624	209873	66302
SRR2736231	PRJNA298332	1018908	306326164	52.3	35	300	301	301	33	32.1	134x	SRR2736231_megahit.fa	222	2105750	2105750	0	0	501	9485	66269	21291	SRR2736231_spades.fa	100	2161679	2161678	1	0	128	21616	246416	112905
SRR2736232	PRJNA298332	1003946	301830870	52.2	52	300	301	301	33	31.4	132x	SRR2736232_megahit.fa	209	2105172	2105172	0	0	500	10072	105168	25086	SRR2736232_spades.fa	92	2160748	2160748	0	0	128	23486	207965	67725
SRR2736233	PRJNA298332	839080	252250690	52.4	35	300	301	301	33	31.6	110x	SRR2736233_megahit.fa	203	2064428	2064428	0	0	503	10169	105278	25334	SRR2736233_spades.fa	107	2110962	2110962	0	0	128	19728	207922	75653
SRR2736234	PRJNA298332	927310	278767807	52.3	35	300	301	301	33	31.9	122x	SRR2736234_megahit.fa	224	2067615	2067615	0	0	501	9230	95095	21262	SRR2736234_spades.fa	99	2111412	2111412	0	0	128	21327	246113	80887
SRR2736235	PRJNA298332	1106958	332776434	52.3	44	300	301	301	33	32.1	146x	SRR2736235_megahit.fa	223	2062011	2062011	0	0	501	9246	105286	23037	SRR2736235_spades.fa	101	2112026	2112026	0	0	128	20911	208122	75714
SRR2736236	PRJNA298332	1031950	310227674	52.4	35	300	301	301	33	32.1	136x	SRR2736236_megahit.fa	224	2058575	2058575	0	0	501	9190	66957	22192	SRR2736236_spades.fa	209	2165688	2165688	0	0	128	10362	208117	70364
SRR2736237	PRJNA298332	1177506	353996078	52.3	35	300	301	301	33	31.7	155x	SRR2736237_megahit.fa	254	2068351	2068351	0	0	500	8143	63454	23325	SRR2736237_spades.fa	277	2193324	2193324	0	0	128	7918	207922	70364
SRR2736238	PRJNA298332	871994	262146838	52.4	35	300	301	301	33	31.6	115x	SRR2736238_megahit.fa	212	2064207	2064207	0	0	503	9736	95095	25265	SRR2736238_spades.fa	210	2160001	2160001	0	0	128	10285	208122	70364
SRR2736239	PRJNA298332	827600	248803577	52.4	42	300	301	301	33	31.8	109x	SRR2736239_megahit.fa	194	2064316	2064316	0	0	500	10640	105283	25087	SRR2736239_spades.fa	179	2151257	2151257	0	0	128	12018	208122	91121
SRR2736240	PRJNA298332	1120626	336901776	52.3	35	300	301	301	33	31.9	148x	SRR2736240_megahit.fa	238	2065779	2065779	0	0	502	8679	67533	22233	SRR2736240_spades.fa	215	2169853	2169853	0	0	128	10092	336465	75529
SRR2736241	PRJNA298332	839948	252495796	52.2	53	300	301	301	33	32	111x	SRR2736241_megahit.fa	206	2111841	2111841	0	0	501	10251	84779	22832	SRR2736241_spades.fa	187	2206429	2206429	0	0	128	11799	245734	89318
SRR2736242	PRJNA298332	750784	225710941	52.2	43	300	301	301	33	31.9	99x	SRR2736242_megahit.fa	211	2072076	2072076	0	0	500	9820	105299	30236	SRR2736242_spades.fa	197	2146247	2146247	0	0	128	10894	208095	70364
SRR2736243	PRJNA298332	930400	279691365	52.2	35	300	301	301	33	31.5	123x	SRR2736243_megahit.fa	217	2116844	2116844	0	0	500	9755	64423	25359	SRR2736243_spades.fa	207	2218413	2218413	0	0	128	10716	207627	131446
SRR2736244	PRJNA298332	933756	280700479	52.1	35	300	301	301	33	31.7	123x	SRR2736244_megahit.fa	212	2113947	2113947	0	0	500	9971	67208	23048	SRR2736244_spades.fa	231	2224513	2224513	0	0	128	9629	207975	60619
SRR2736245	PRJNA298332	581556	174822207	52.1	35	300	301	301	33	31.8	76x	SRR2736245_megahit.fa	196	2116004	2116004	0	0	504	10795	84779	22855	SRR2736245_spades.fa	322	2278457	2278457	0	0	128	7075	207787	63321
SRR2736246	PRJNA298332	1100696	330930290	52	41	300	301	301	33	33.1	145x	SRR2736246_megahit.fa	212	2106368	2106368	0	0	500	9935	64459	22824	SRR2736246_spades.fa	239	2231831	2231831	0	0	128	9338	207975	67324
SRR2736247	PRJNA298332	1148644	345310271	52.2	52	300	301	301	33	33.3	151x	SRR2736247_megahit.fa	217	2100201	2100201	0	0	502	9678	84779	23048	SRR2736247_spades.fa	199	2214037	2214037	0	0	128	11125	278161	71272
SRR2736248	PRJNA298332	1392000	418479798	52.3	35	300	301	301	33	32.7	184x	SRR2736248_megahit.fa	252	2097721	2097721	0	0	500	8324	67144	18531	SRR2736248_spades.fa	294	2260081	2260081	0	0	128	7687	207975	71272
SRR2736249	PRJNA298332	1147628	345016210	52.4	38	300	301	301	33	33.1	151x	SRR2736249_megahit.fa	221	2053803	2053803	0	0	502	9293	67000	23251	SRR2736249_spades.fa	221	2158071	2158071	0	0	128	9765	308528	84615
SRR2736250	PRJNA298332	2381670	716083490	52.1	46	300	301	301	33	33	314x	SRR2736250_megahit.fa	1125	1994785	1994785	0	0	500	1773	18048	2629	SRR2736250_spades.fa	524	2383678	2383678	0	0	128	4549	278075	91243
SRR2736251	PRJNA298332	592404	178073573	52.6	35	300	301	301	33	32.7	78x	SRR2736251_megahit.fa	198	2064540	2064540	0	0	500	10426	105258	23268	SRR2736251_spades.fa	168	2145140	2145140	0	0	128	12768	207892	75940
SRR2736252	PRJNA298332	759592	228349981	52.6	43	300	301	301	33	33.1	100x	SRR2736252_megahit.fa	208	2068534	2068534	0	0	500	9944	95095	25386	SRR2736252_spades.fa	182	2146786	2146786	0	0	128	11795	207892	81301
SRR2736253	PRJNA298332	643082	193322980	52.6	35	300	301	301	33	32.5	85x	SRR2736253_megahit.fa	213	2071314	2071314	0	0	500	9724	95095	25386	SRR2736253_spades.fa	209	2169881	2169881	0	0	128	10382	193043	86343
SRR2736254	PRJNA298332	890282	267654230	52.4	59	300	301	301	33	33.5	117x	SRR2736254_megahit.fa	188	2106952	2106952	0	0	508	11207	67220	25344	SRR2736254_spades.fa	155	2185419	2185419	0	0	128	14099	207970	58669
SRR2736255	PRJNA298332	1165022	350280580	53.3	40	300	301	301	33	32.8	154x	SRR2736255_megahit.fa	260	2047602	2047602	0	0	503	7875	63955	18250	SRR2736255_spades.fa	265	2178073	2178073	0	0	128	8219	305661	86326
SRR2736256	PRJNA298332	1084448	325960735	52.6	48	300	301	301	33	32.7	143x	SRR2736256_megahit.fa	223	2050064	2050064	0	0	500	9193	95095	22165	SRR2736256_spades.fa	277	2184766	2184766	0	0	128	7887	207868	84615

SRR2736257	PRJNA298332	1111292	334105555	52.4	53	300	301	301	33	32.9	146x	SRR2736257_megahit.fa	221	2098313	2098313	0	0	500	9494	66313	22849	SRR2736257_spades.fa	248	2228545	2228545	0	0	128	8986	210677	78518
SRR2736258	PRJNA298332	916298	275443896	52.3	35	300	301	301	33	32.9	121x	SRR2736258_megahit.fa	216	2110157	2110157	0	0	501	9769	149180	22661	SRR2736258_spades.fa	161	2194355	2194355	0	0	128	13629	207791	65449
SRR2736259	PRJNA298332	1016758	305627516	52.4	35	300	301	301	33	32.6	134x	SRR2736259_megahit.fa	226	2110688	2110688	0	0	507	9339	66298	22853	SRR2736259_spades.fa	199	2209797	2209797	0	0	128	11104	207794	106386
SRR2736260	PRJNA298332	1125876	338387171	52.4	35	300	301	301	33	32.9	148x	SRR2736260_megahit.fa	212	2099407	2099407	0	0	500	9902	64491	20431	SRR2736260_spades.fa	229	2218715	2218715	0	0	128	9688	208019	71000
SRR2736261	PRJNA298332	994602	298909185	52.5	35	300	301	301	33	32.8	131x	SRR2736261_megahit.fa	210	2107606	2107606	0	0	503	10036	67306	23134	SRR2736261_spades.fa	177	2199407	2199407	0	0	128	12426	261982	88407
SRR2736262	PRJNA298332	937606	281842962	52.6	35	300	301	301	33	32.9	123x	SRR2736262_megahit.fa	202	2102766	2102766	0	0	508	10409	67307	25251	SRR2736262_spades.fa	197	2209396	2209396	0	0	128	11215	213120	95613
SRR2736263	PRJNA298332	2418370	727124099	52.3	35	300	301	301	33	32.5	319x	SRR2736263_megahit.fa	1016	2062520	2062520	0	0	500	2030	20649	3295	SRR2736263_spades.fa	603	2395931	2395931	0	0	128	3973	278163	71272
SRR2736264	PRJNA298332	902098	271154518	52.4	59	300	301	301	33	32.9	119x	SRR2736264_megahit.fa	214	2111458	2111458	0	0	500	9866	66298	23048	SRR2736264_spades.fa	196	2211800	2211800	0	0	128	11284	278135	70735
SRR2736265	PRJNA298332	515020	154737508	52.4	35	300	301	301	33	32.7	68x	SRR2736265_megahit.fa	181	2112380	2112380	0	0	500	11670	149183	25386	SRR2736265_spades.fa	126	2178900	2178900	0	0	128	17292	246184	66358
SRR2736266	PRJNA298332	1045908	314460777	52.9	38	300	301	301	33	33.3	138x	SRR2736266_megahit.fa	268	2102562	2102562	0	0	502	7845	75949	17144	SRR2736266_spades.fa	192	2211632	2211632	0	0	128	11518	246013	66841
SRR2736267	PRJNA298332	1348666	405488779	52.7	39	300	301	301	33	33	178x	SRR2736267_megahit.fa	273	2105817	2105817	0	0	500	7713	64329	17714	SRR2736267_spades.fa	275	2256829	2256829	0	0	128	8206	322792	103133
SRR2736268	PRJNA298332	1297568	390124718	52.3	46	300	301	301	33	33.4	171x	SRR2736268_megahit.fa	250	2113773	2113773	0	0	500	8455	113795	18559	SRR2736268_spades.fa	231	2231461	2231461	0	0	128	9660	254143	88402
SRR2736269	PRJNA298332	1351050	406200135	52.9	39	300	301	301	33	32.9	178x	SRR2736269_megahit.fa	310	2050555	2050555	0	0	502	6614	77374	16765	SRR2736269_spades.fa	296	2204628	2204628	0	0	128	7448	208097	70364
SRR2736270	PRJNA298332	1049138	315390068	52.4	48	300	301	301	33	33.2	138x	SRR2736270_megahit.fa	226	2112711	2112711	0	0	500	9348	64511	21164	SRR2736270_spades.fa	251	2233142	2233142	0	0	128	8896	207925	67221
SRR2736271	PRJNA298332	1598952	480752426	52.9	37	300	301	301	33	33	211x	SRR2736271_megahit.fa	388	2052243	2052243	0	0	500	5289	39031	11756	SRR2736271_spades.fa	320	2220122	2220122	0	0	128	6937	220507	78106
SRR2736272	PRJNA298332	940048	282549690	52.6	48	300	301	301	33	33	124x	SRR2736272_megahit.fa	192	2060042	2060042	0	0	509	10729	95092	24729	SRR2736272_spades.fa	222	2168667	2168667	0	0	128	9768	208098	70364
SRR2736273	PRJNA298332	1417536	426204214	52.6	42	300	301	301	33	32.6	187x	SRR2736273_megahit.fa	279	2099360	2099360	0	0	500	7524	71953	18845	SRR2736273_spades.fa	273	2239479	2239479	0	0	128	8203	207635	76620
SRR2736274	PRJNA298332	1550976	466322239	52.6	56	300	301	301	33	32.6	205x	SRR2736274_megahit.fa	352	2108070	2108070	0	0	500	5988	47627	14140	SRR2736274_spades.fa	334	2271728	2271728	0	0	128	6801	207663	70141
SRR2736275	PRJNA298332	851934	256145395	52.8	50	300	301	301	33	33.1	112x	SRR2736275_megahit.fa	210	2054464	2054464	0	0	503	9783	105286	23244	SRR2736275_spades.fa	219	2156763	2156763	0	0	128	9848	207868	84615
SRR2736276	PRJNA298332	904612	271950475	52.5	35	300	301	301	33	33	119x	SRR2736276_megahit.fa	211	2113553	2113553	0	0	500	10016	66262	20780	SRR2736276_spades.fa	189	2211850	2211850	0	0	128	11702	228356	77149
SRR2736277	PRJNA298332	1248082	375241966	52.3	48	300	301	301	33	32.9	165x	SRR2736277_megahit.fa	265	2109334	2109334	0	0	500	7959	53573	18567	SRR2736277_spades.fa	245	2234274	2234274	0	0	128	9119	246194	71272
SRR2736278	PRJNA298332	904156	271752013	52.5	42	300	301	301	33	32.7	119x	SRR2736278_megahit.fa	202	2120693	2120693	0	0	504	10498	113676	25386	SRR2736278_spades.fa	193	2214474	2214474	0	0	128	11473	426987	81125
SRR2736279	PRJNA298332	1511340	454392896	52.7	35	300	301	301	33	33.1	199x	SRR2736279_megahit.fa	510	2105651	2105651	0	0	500	4128	29026	8410	SRR2736279_spades.fa	261	2235342	2235342	0	0	128	8564	207982	66302
SRR2736280	PRJNA298332	1430684	430139855	52.5	39	300	301	301	33	32.5	189x	SRR2736280_megahit.fa	303	2089805	2089805	0	0	500	6897	60033	17455	SRR2736280_spades.fa	293	2251617	2251617	0	0	128	7684	277693	85537
SRR2736281	PRJNA298332	1445448	434584018	52.5	45	300	301	301	33	33	191x	SRR2736281_megahit.fa	330	2144725	2144725	0	0	500	6499	48407	16621	SRR2736281_spades.fa	279	2290868	2290868	0	0	128	8210	210499	77910
SRR2736282	PRJNA298332	1033570	310703829	52.4	49	300	301	301	33	32.9	136x	SRR2736282_megahit.fa	217	2113579	2113579	0	0	505	9739	113676	23341	SRR2736282_spades.fa	206	2219729	2219729	0	0	128	10775	248740	81211
SRR2736283	PRJNA298332	1317318	396069582	53	41	300	301	301	33	32.6	174x	SRR2736283_megahit.fa	311	2118454	2118454	0	0	500	6811	47628	17033	SRR2736283_spades.fa	278	2255496	2255496	0	0	128	8113	207934	76402
SRR2736284	PRJNA298332	836112	251391308	53.1	40	300	301	301	33	32.7	110x	SRR2736284_megahit.fa	238	2121517	2121517	0	0	503	8913	66215	23049	SRR2736284_spades.fa	175	2202058	2202058	0	0	128	12583	228289	112221
SRR2736285	PRJNA298332	1411376	424362416	52.9	35	300	301	301	33	32.5	186x	SRR2736285_megahit.fa	326	2116146	2116146	0	0	500	6491	47595	16653	SRR2736285_spades.fa	307	2264968	2264968	0	0	128	7377	246074	74290
SRR2736286	PRJNA298332	1338114	402318345	52.3	41	300	301	301	33	32.6	176x	SRR2736286_megahit.fa	269	2113994	2113994	0	0	500	7858	65701	19521	SRR2736286_spades.fa	307	2264124	2264124	0	0	128	7374	222136	74227
SRR2736287	PRJNA298332	466568	140248852	52.4	35	300	301	301	33	33	61x	SRR2736287_megahit.fa	178	2125580	2125580	0	0	511	11941	113676	34723	SRR2736287_spades.fa	134	2182671	2182671	0	0	128	16288	322257	66964
SRR2736288	PRJNA298332	1143912	343899740	52.4	39	300	301	301	33	32.3	151x	SRR2736288_megahit.fa	240	2112684	2112684	0	0	500	8802	113676	20155	SRR2736288_spades.fa	216	2223921	2223921	0	0	128	10295	207952	67282
SRR2736289	PRJNA298332	1480224	445010879	52.5	39	300	301	301	33	32.6	195x	SRR2736289_megahit.fa	336	2050669	2050669	0	0	500	6103	40815	13634	SRR2736289_spades.fa	317	2219000	2219000	0	0	128	7000	207941	85530
SRR2736290	PRJNA298332	1519978	456958191	52.6	39	300	301	301	33	32.6	200x	SRR2736290_megahit.fa	361	2046959	2046959	0	0	500	5670	48805	13691	SRR2736290_spades.fa	305	2204243	2204243	0	0	128	7227	308541	86946
SRR2736291	PRJNA298332	1375190	413389028	52.3	45	300	301	301	33	33	181x	SRR2736291_megahit.fa	274	2105574	2105574	0	0	503	7684	66215	18291	SRR2736291_spades.fa	232	2240892	2240892	0	0	128	9659	322607	77934
SRR2736292	PRJNA298332	1628120	489453275	52.3	35	300	301	301	33	32.4	215x	SRR2736292_megahit.fa	375	2106266	2106266	0	0	503	5616	47503	13165	SRR2736292_spades.fa	318	2275905	2275905	0	0	128	7156	254204	103787
SRR2736293	PRJNA298332	1470228	441991026	52.5	39	300	301	301	33	32.8	194x	SRR2736293_megahit.fa	302	2050934	2050934	0	0	501	6791	64436	16725	SRR2736293_spades.fa	296	2199762	2199762	0	0	128	7431	308779	64579
SRR2736294	PRJNA298332	1296934	389886496	52.5	35	300	301	301	33	32.9	171x	SRR2736294_megahit.fa	271	2061567	2061567	0	0	501	7607	95092	18531	SRR2736294_spades.fa	237	2178190	2178190	0	0	128	9190	208107	67063
SRR2736295	PRJNA298332	1079290	324452897	52.4	44	300	301	301	33	32.7	142x	SRR2736295_megahit.fa	219	2109020	2109020	0	0	501	9630	113676	23250	SRR2736295_spades.fa	215	2221702	2221702	0	0	128	10333	207962	85348
SRR2736296	PRJNA298332	1370400	411977638	52.4	39	300	301	301	33	32.2	181x	SRR2736296_megahit.fa	286	2109617	2109617	0	0	502	7376	58469	18531	SRR2736296_spades.fa	294	2258123	2258123	0	0	128	7680	236827	85891
SRR2736297	PRJNA298332	908864	273220252	52.4	39	300	301	301	33	32.6	120x	SRR2736297_megahit.fa	214	2121390	2121390	0	0	501	9913	105121	23049	SRR2736297_spades.fa	216	2221650	2221650	0	0	128	10285	209633	81221
SRR2736298	PRJNA298332	1152696	346564580	52.4	57	300	301	301	33	32.8	152x	SRR2736298_megahit.fa	235	2054530	2054530	0	0	500	8742	86595	18601	SRR2736298_spades.fa	220	2171147	2171147	0	0	128	9868	308765	84736
SRR2736299	PRJNA298332	1620694	487266147	52.1	56	300	301	301	33	32.7	214x	SRR2736299_megahit.fa	356	2105809	2105809	0	0	500	5915	54831	12193	SRR2736299_spades.fa	290	2260260	2260260	0	0	128	7794	211108	81298
SRR2736300	PRJNA298332	1628702	489671248	52.3	35	300	301	301	33	32.9	215x	SRR2736300_megahit.fa	332	2101856	2101856	0	0	500	6330	66462	14068	SRR2736300_spades.fa	272	2253196	2253196	0	0	128	8283	278076	83687
SRR2736301	PRJNA298332	1465182	440507051	51.8	64	300	301	301	33	33	193x	SRR2736301_megahit.fa	282	2123935	2123935	0	0	500	7531	42536	18397	SRR2736301_spades.fa	199	2240708	2240708	0	0	128	11259	210801	80077
SRR2736302	PRJNA298332	1706488	513034575	52	35	300	301	301	33	32.7	225x	SRR2736302_megahit.fa	388	2112321	2112321	0	0	500	5444	51948	10685	SRR2736302_spades.fa	348	2280992	2280992	0	0	128	6554	207934	89471
SRR2736303	PRJNA298332	1502706	451774087	52.1	36	300	301	301	33	32.9	198x	SRR2736303_megahit.fa	281	2108431	2108431	0	0	500	7503	63454	16842	SRR2736303_spades.fa	245	2242786	2242786	0	0	128	9154	254161	81226
SRR2736304	PRJNA298332	1683256	506072382	52.4	35	300	301	301	33	32.9	222x	SRR2736304_megahit.fa	375	2050059	2050059	0	0	501	5466	36799	11518	SRR2736304_spades.fa	292	2202659	2202659	0	0	128	7543	308822	67063
SRR2736305	PRJNA298332	1499358	450783603	52.2	51	300	301	301	33	32.6	198x	SRR2736305_megahit.fa	300	2052888	2052888	0	0	500	6842	47234	15523	SRR2736305_spades.fa	278	2195536	2195536	0	0	128	7897	308768	76726
SRR2736306	PRJNA298332	1394886	419375371	52.2	79	300	301	301	33	32.7	184x	SRR2736306_megahit.fa	281	2103138	2103138	0	0	503	7484	49104	17942	SRR2736306_spades.fa	248	2236943	2236943	0	0	128	9019	278072	88368
ERR1426708	PRJEB14168	1257330	186669064	52	35	148	151	151	33	32.6	82x	AUSMDU00004452_megahit.fa	214	2102350	2102350	0	0	502	9824	67313	21734	AUSMDU00004452_spades.fa	142	2159215	2159215	0	0	128	15205	175686	55643
ERR1426709	PRJEB14168	1428856	211743221	51.8	35	148	151	151	33	32.5	93x	AUSMDU00004453_megahit.fa	181	2104301	2104301	0	0	514	11625	105417	25158	AUSMDU00004453_spades.fa	140	2164091	2164091	0	0	128	15457	207734	53407
ERR1426710	PRJEB14168	1349060	200393465	52	35	148	151	151	33	32.5	88x	AUSMDU00004454_megahit.fa	226	2100163	2100163	0	0	500	9292	148986	19287	AUSMDU00004454_spades.fa	144	2156827	2156827	0	0	128	14977	175823	44163
ERR1426711	PRJEB14168	1420934	210699102	52.1	35	148	151	151	33	32.5	92x	AUSMDU00004455_megahit.fa	191	2140078	2140078	0	0	505	11204	105146	23038	AUSMDU00004455_spades.fa	157	2194830	2194830	0	0	128	13979	205218	55196
ERR1426712	PRJEB14168	1198244	276530607	50.9	32	230	301	301	33	31.4	121x	AUSMDU00004456_megahit.fa	224	2102492	2102492	0	0	501	9386	67193	21302	AUSMDU00004456_spades.fa	160	2174505	2174505	0	0	128	13590	208056	55724
ERR1426713	PRJEB14168	1621074	360421917	51	32	222	301	301	33	32.1	158x	AUSMDU00004457_megahit.fa	227	2100040	2100040	0	0	503	9251	67226	20193	AUSMDU00004457_spades.fa	164	2179952	2179952	0	0	128	13292	208056	63307
ERR1426714	PRJEB14168	1609380	355049002	50.9	32	220	301	301	33	31.8	156x	AUSMDU00004458_megahit.fa	230	2107796	2107796	0	0	503	9164	64485	21302	AUSMDU00004458_spades.fa	171	2179833	2179833	0	0	128	12747	208044	55218
ERR1426715	PRJEB14168	1388116	206254961	52.3	35	148	151	151	33	32.5	90x	AUSMDU00004459_megahit.fa	200	2086980	2086980	0	0	501	10434	64653	23053	AUSMDU00004459_spades.fa	136	2136459	2136459	0	0	128	15709	205100	48782
ERR1426716	PRJEB14168	1260336	187282994	52.1	35	148	151	151	33	32.6	82x	AUSMDU00004460_megahit.fa	206	2153148	2153148	0	0	502	10452	105296	23024	AUSMDU00004460_spades.fa	150	2206876	2206876	0	0	128	14712	154371	42005
ERR1426717	PRJEB14168	1367736	201380954	51.4	35	147	151	151	33	32.7	88x	AUSMDU00004461_megahit.fa	191	2093060	2093060	0	0	501	10958	62826	25111	AUSMDU00004461_spades.fa	135	2153071	2153071	0	0	128	15948	207758	42359
ERR1426718	PRJEB14168	1182168	175524570	52.4	35	148	151	151	33	32.5	77x	AUSMDU00004462_megahit.fa	200	2063142	2063142	0	0	502	10315	105262	22905	AUSMDU00004462_spades.fa	157	2122678	2122678	0	0	128	13520	119802	48911
ERR1426719	PRJEB14168	1184376	270952078	51.2	32	228	301	301	33	30.9	119x	AUSMDU00004463_megahit.fa	222	2107097	2107097	0	0	500	9491	67383	21323	AUSMDU00004463_spades.fa	131	2162453	2162453	0	0	128	16507	208086	60651
ERR1426720	PRJEB14168	1266684	188191631	52.2	35	148	151	151	33	32.5	82x	AUSMDU00004464_megahit.fa	206	2112643	2112643	0	0	500	10255	66307	23340	AUSMDU00004464_spades.fa	119	2160967	2160967	0	0	128	18159	207959	56314
ERR1426721	PRJEB14168	1467890	218194569	52.5	35	148	151	151	33	32.3	95x	AUSMDU00004465_megahit.fa	190	2064994	2064994	0	0	507	10868	105253	22766	AUSMDU00004465_spades.fa	130	2115511	2115511	0	0	128	16273	140348	47389
ERR1426722	PRJEB14168	1488960	290675032	52.7	35	195	301	301	33	33.5	127x	AUSMDU00004466_megahit.fa	213	2064335	2064335	0	0	500	9691	62958	23257	AUSMDU00004466_spades.fa	133	2111215	2111215	0	0	128	15873	208118	59409
ERR1426723	PRJEB14168	1359712	201965470	52	35	148	151	151	33	32.3	88x	AUSMDU00004467_megahit.fa	209	2157473	2157473	0	0	505	10322	149068	21699	AUSMDU00004467_spades.fa	141	2208364	2208364	0	0	128	15662	173925	40518
ERR1426724	PRJEB14168	1301070	193742029	51.5	35	148	151	151	33	32.1	85x	AUSMDU00004468_megahit.fa	203	2154294	2154294	0	0	507	10612	149103	21052	AUSMDU00004468_spades.fa	178	2222089	2222089	0	0	128	12483	174081	42218
ERR1426725	PRJEB14168	1356380	201039299	52.2	35	148	151	151	33	32.6	88x	AUSMDU00004469_megahit.fa	206	2145757	2145757	0	0	501	10416	64074	21039	AUSMDU00004469_spades.fa	153	2199939	2199939	0	0	128	14378	172935	47175
ERR1426726	PRJEB14168	1386374	204840729	52.4	35	147	151	151	33	32.7	90x	AUSMDU00004470_megahit.fa	189	2056255	2056255	0	0	507	10879	66176	21735	AUSMDU00004470_spades.fa	142	2107383	2107383	0	0	128	14840	121782	43997
ERR1426727	PRJEB14168	1267900	187938483	52.5	35	148	151	151	33	32.4	82x	AUSMDU00004471_megahit.fa	200	2065516	2065516	0	0	519	10327	105144	23048	AUSMDU00004471_spades.fa	143	2116718	2116718	0	0	128	14802	207934	47503
ERR1426728	PRJEB14168	1340582	199494387	52.6	35	148	151	151	33	32.3	87x	AUSMDU00004472_megahit.fa	208	2092526	2092526	0	0	502	10060	67345	21035	AUSMDU00004472_spades.fa	131	2151003	2151003	0	0	128	16419	106892	47048
ERR1426729	PRJEB14168	1438780	213668192	52.2	35	148	151	151	33	32.4	93x	AUSMDU00004473_megahit.fa	193	2123181	2123181	0	0	500	11000	105245	24728	AUSMDU00004473_spades.fa	143	2172113	2172113	0	0	128	15189	152847	49469
ERR1426730	PRJEB14168	1453724	215932568	52.5	35	148	151	151	33	32.5	94x	AUSMDU00004474_megahit.fa	215	2111655	2111655	0	0	500	9821	66307	20723	AUSMDU00004474_spades.fa	142	2164311	2164311	0	0	128	15241	207904	46648
ERR1426731	PRJEB14168	1384554	205727690	52.5	35	148	151	151	33	32.6	90x	AUSMDU00004475_megahit.fa	190	2103488	2103488	0	0	506	11070	105287	22009	AUSMDU00004475_spades.fa	158	2160843	2160843	0	0	128	13676	116625	45452
ERR1426732	PRJEB14168	1310216	192998080	52	35	147	151	151	33	32.4	84x	AUSMDU00004476_megahit.fa	183	2054562	2054562	0	0	500	11227	105061	22079	AUSMDU00004476_spades.fa	138	2106648	2106648	0	0	128	15265	170092	55526
ERR1426733	PRJEB14168	1478220	218150603	52.3	35	147	151	151	33	32.5	95x	AUSMDU00004477_megahit.fa	201	2114846	2114846	0	0	510	10521	64508	20437	AUSMDU00004477_spades.fa	121	2162140	2162140	0	0	128	17868	208064	52921
ERR1426734	PRJEB14168	1305072	193376730	51.9	35	148	151	151	33	32.4	85x	AUSMDU00004478_megahit.fa	189	2145702	2145702	0	0	509	11352	149187	23584	AUSMDU00004478_spades.fa	145	2204954	2204954	0	0	128	15206	173521	41449
ERR1426735	PRJEB14168	1322104	196270877	52.4	35	148	151	151	33	32.4	86x	AUSMDU00004479_megahit.fa	189	2091570	2091570	0	0	518	11066	162166	24606	AUSMDU00004479_spades.fa	150	2142314	2142314	0	0	128	14282	169972	44268
ERR1426736	PRJEB14168	1414280	210009027	52.6	35	148	151	151	33	32.5	92x	AUSMDU00004480_megahit.fa	194	2055970	2055970	0	0	519	10597	105552	21036	AUSMDU00004480_spades.fa	123	2105065	2105065	0	0	128	17114	205267	45681
ERR1426737	PRJEB14168	1412420	209801154	52.4	35	148	151	151	33	32.4	92x	AUSMDU00004481_megahit.fa	193	2112925	2112925	0	0	541	10947	105236	22896	AUSMDU00004481_spades.fa	146	2167331	2167331	0	0	128	14844	207871	48814
ERR1426738	PRJEB14168	1504090	218481403	53.2	35	145	151	151	33	33.4	96x	AUSMDU00004482_megahit.fa	258	2092391	2092391	0	0	503	8110	68176	16768	AUSMDU00004482_spades.fa	191	2148529	2148529	0	0	128	11248	136826	27954
ERR1426739	PRJEB14168	1641468	237204922	53.1	35	144	151	151	33	33.5	104x	AUSMDU00004483_megahit.fa	225	2059254	2059254	0	0	508	9152	105276	18487	AUSMDU00004483_spades.fa	155	2110496	2110496	0	0	128	13616	114448	39796
ERR1426740	PRJEB14168	1324296	181698021	52.6	35	137	151	151	33	33.7	79x	AUSMDU00004484_megahit.fa	202	2114561	2114561	0	0	500	10468	67473	20348	AUSMDU00004484_spades.fa	194	2173226	2173226	0	0	128	11202	174739	40499
ERR1426741	PRJEB14168	1365576	194012451	52.8	35	142	151	151	33	33.5	85x	AUSMDU00004485_megahit.fa	196	2090381	2090381	0	0	512	10665	105272	20425	AUSMDU00004485_spades.fa	178	2146733	2146733	0	0	128	12060	174719	39248
ERR1426742	PRJEB14168	1423784	205606072	52.9	35	144	151	151	33	33.5	90x	AUSMDU00004486_megahit.fa	212	2081393	2081393	0	0	503	9817	64291	19494	AUSMDU00004486_spades.fa	171	2132179	2132179	0	0	128	12468	172623	39268
ERR1426743	PRJEB14168	1460672	210452155	52.8	35	144	151	151	33	33.4	92x	AUSMDU00004487_megahit.fa	269	2141765	2141765	0	0	506	7961	46763	15563	AUSMDU00004487_spades.fa	231	2193639	2193639	0	0	128	9496	78358	23618
ERR1426744	PRJEB14168	1578596	226325822	52.7	35	143	151	151	33	33.4	99x	AUSMDU00004488_megahit.fa	252	2149822	2149822	0	0	515	8531	78218	17569	AUSMDU00004488_spades.fa	183	2204201	2204201	0	0	128	12044	101774	40677
ERR1426745	PRJEB14168	733252	107818240	52.6	35	147	151	151	33	33.2	47x	AUSMDU00004489_megahit.fa	304	2135951	2135951	0	0	505	7026	47089	14377	AUSMDU00004489_spades.fa	228	2199433	2199433	0	0	128	9646	102943	23958
ERR1426746	PRJEB14168	1524966	222290928	53	35	145	151	151	33	33.2	97x	AUSMDU00004490_megahit.fa	238	2101471	2101471	0	0	508	8829	62822	17132	AUSMDU00004490_spades.fa	184	2151980	2151980	0	0	128	11695	76466	32714
ERR1426747	PRJEB14168	1431392	200742341	52.8	35	140	151	151	33	33.6	88x	AUSMDU00004491_megahit.fa	232	2150563	2150563	0	0	500	9269	58237	18060	AUSMDU00004491_spades.fa	189	2207238	2207238	0	0	128	11678	85839	31799
ERR1426748	PRJEB14168	1778494	258523361	52.9	35	145	151	151	33	33.3	113x	AUSMDU00004492_megahit.fa	227	2082818	2082818	0	0	514	9175	101807	18150	AUSMDU00004492_spades.fa	144	2136336	2136336	0	0	128	14835	114023	41223
ERR1426749	PRJEB14168	1596402	230425633	53.1	35	144	151	151	33	33.4	101x	AUSMDU00004493_megahit.fa	217	2097638	2097638	0	0	513	9666	101815	18131	AUSMDU00004493_spades.fa	173	2147647	2147647	0	0	128	12414	101899	35911
ERR1426750	PRJEB14168	1807424	262129638	52.8	35	145	151	151	33	33.3	115x	AUSMDU00004494_megahit.fa	214	2105463	2105463	0	0	541	9838	64336	20251	AUSMDU00004494_spades.fa	170	2160618	2160618	0	0	128	12709	152700	40636
ERR1426751	PRJEB14168	2148598	317155004	53	35	147	151	151	33	33	139x	AUSMDU00004495_megahit.fa	195	2060092	2060092	0	0	505	10564	95095	23258	AUSMDU00004495_spades.fa	123	2107242	2107242	0	0	128	17132	207951	52558
ERR1426752	PRJEB14168	1602896	235335261	52.3	35	146	151	151	33	32.6	103x	AUSMDU00004496_megahit.fa	212	2182593	2182593	0	0	523	10295	149120	21036	AUSMDU00004496_spades.fa	128	2225824	2225824	0	0	134	17389	202729	49389
ERR1426753	PRJEB14168	1738728	256233344	52.5	35	147	151	151	33	32.6	112x	AUSMDU00004497_megahit.fa	189	2156250	2156250	0	0	515	11408	93133	23046	AUSMDU00004497_spades.fa	121	2208171	2208171	0	0	128	18249	207874	50821
ERR1426754	PRJEB14168	1780738	262279149	52.1	35	147	151	151	33	32.9	115x	AUSMDU00004498_megahit.fa	184	2153508	2153508	0	0	514	11703	108566	25315	AUSMDU00004498_spades.fa	104	2204464	2204464	0	0	128	21196	207850	59586
ERR1426755	PRJEB14168	2139626	315200781	52.7	35	147	151	151	33	32.9	138x	AUSMDU00004499_megahit.fa	192	2061828	2061828	0	0	506	10738	95092	23259	AUSMDU00004499_spades.fa	113	2112159	2112159	0	0	128	18691	207944	59012
ERR1426756	PRJEB14168	2125750	315087837	52.7	35	148	151	151	33	33	138x	AUSMDU00004500_megahit.fa	201	2105980	2105980	0	0	508	10477	66963	23051	AUSMDU00004500_spades.fa	147	2163019	2163019	0	0	128	14714	203224	57496
ERR1426757	PRJEB14168	3493288	512966416	52.9	35	146	151	151	33	33.2	225x	AUSMDU00004501_megahit.fa	223	2079262	2079262	0	0	512	9324	66180	20872	AUSMDU00004501_spades.fa	136	2146413	2146413	0	0	128	15782	208048	59331
